# Development of Naphthalene-Derivative Bis-QACs as Potent Antimicrobials: Unraveling Structure–Activity Relationship and Microbiological Properties

**DOI:** 10.3390/molecules29235526

**Published:** 2024-11-22

**Authors:** Nikita A. Frolov, Mary A. Seferyan, Elena V. Detusheva, Elizabeth Son, Ilya G. Kolmakov, Alena S. Kartseva, Victoria V. Firstova, Anatoly N. Vereshchagin, Michail N. Elinson

**Affiliations:** 1 N. D. Zelinsky Institute of Organic Chemistry, Russian Academy of Sciences, Leninsky Prospect 47, 119991 Moscow, Russia; nfrolov@ioc.ac.ru (N.A.F.); marysev@ioc.ac.ru (M.A.S.); ikolvilya@outlook.com (I.G.K.); 2 State Research Center for Applied Microbiology and Biotechnology, Obolensk, 142279 Serpukhov, Russia; klub@bk.ru (E.V.D.); e_swon2001@mail.ru (E.S.); firstova@obolensk.org (V.V.F.); 3 Faculty of Chemistry, Lomonosov Moscow State University, Leninskie Gory, 1-3, 119991 Moscow, Russia

**Keywords:** quaternary ammonium compounds, antibacterials, antibiofilm agents, SAR, antiseptics, disinfectant, multi-resistant bacteria, ESKAPE

## Abstract

While the pandemic is behind us, the world community faces a global threat of bacterial resistance outbreak. One of the key ways to combat the spread of multi-resistant bacteria is infection prevention and control tactics using modern antiseptic and disinfectant compositions. Herein, we continue the path to unravel the structure–activity relationship (SAR) of potent pyridine-derived biocide class bis-quaternary ammonium compounds (QACs). In this study, twenty dihydroxynaphthalene-derivative bis-QACs were subjected to extensive microbiological analysis on planktonic cells and biofilms of the ESKAPE microorganisms. Among them, hit compounds were superior in their bacteriostatic and bactericidal action to commercial mono-QACs and were comparable to the best bis-QAC antiseptic on the market. SAR analysis indicated that the linker conformation does not significantly affect the activity, though structure symmetry and especially lipophilicity had an influence on antibacterial performance. Furthermore, we delve deeper in investigation of the antimicrobial potential of bis-QACs and conducted a variety of assays, including time–kill kinetics, bacterial resistance formation, cell morphology, and cytotoxicity. Studies showed promising results for compounds 5d and 6d, indicating 2 to 3-fold less cytotoxicity and hemotoxicity compared to commercial QACs. Moreover, SEM imaging revealed that bis-QACs can cause severe membrane damage to *S. aureus* and *P. aeruginosa* strains, confirming great potential of novel compounds as antiseptic and disinfectant.

## 1. Introduction

In 2023, the World Health Organization declared an end to COVID-19 emergence response, marking the decline of the four-year-long pandemic [1,2]. However, the aftermath of COVID-19 has had a significant impact on people’s physical and mental health [3], the technological and economic landscape [4], health law [5], etc. However, one of the most serious consequences is the rapidly hastened uprising of global antimicrobial resistance (AMR) [6,7,8,9,10,11]. A particularly high increase in the AMR rate was reported for Gram-negative pathogens from the ESKAPE group: *Escherichia coli*, *Acinetobacter baumannii*, and *Klebsiella pneumoniae* [12]. Combating AMR requires a multifaceted approach across different fields to reduce the spread of resistance and spur innovation in new antimicrobial treatments and disease prevention tactics [13].

Infection prevention and control practices are critical to tackle the spread of AMR, including implementation and adherence to disinfection and cleanliness measures [14]. Unfortunately, the market for disinfectants and antiseptics is currently lacking significant changes or innovations, while the focus of the scientific and pharmaceutical communities is more often shifting towards the development of new antibiotics. Thus, high-quality surface disinfection will require increasingly higher concentrations of active substances. This problem is especially critical for the fight against pathogens of hospital-acquired multidrug-resistant infections, which rapidly spiked during the COVID-19 pandemic [15,16]. Therefore, new research in the development of antiseptic and disinfectant compounds (or biocides) is crucial for controlling AMR.

**Figure 1:**
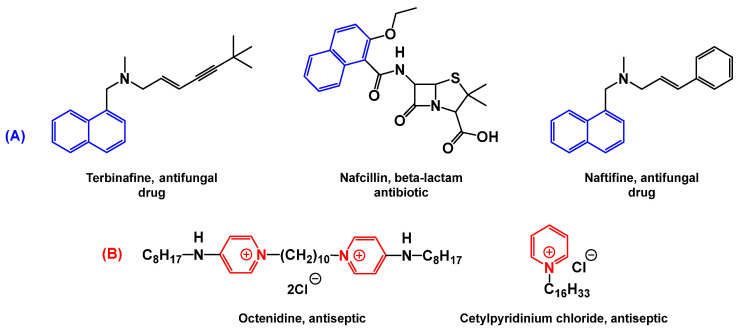
Commercially available naphthalene- (**A**) and pyridine-derived (**B**) antimicrobial agents.

Naphthalene is a versatile platform in medicinal chemistry [17], exhibiting a wide range of biological activities including anticancer [18,19,20], antimicrobial [21,22,23], anti-inflammatory [24,25], antioxidant [26,27], antitubercular [28], anti-neurodegenerative [29], etc. The activity of naphthalene is often associated with its metabolites, which can covalently interact with cellular proteins, influencing various biochemical pathways that lead to therapeutic effects [17]. In addition, naphthalene derivatives are a convenient platform for structural modification [30,31]. Therefore, naphthalene scaffold is extensively explored due to its ability to form various derivatives with diverse pharmacological activities. Several naphthalene-based molecules have been approved by the FDA and are marketed as antimicrobial agents, such as naftifine [32], nafcillin [33], and terbinafine [34] (Figure 1A).

The pyridinium moiety is one of the main structure components of quaternary ammonium compounds (QACs)—widely known surfactants used mainly as antiseptics, disinfectants, preservatives, and various composites and materials [35,36,37,38,39,40,41,42,43]. For example, cetylpyridinium chloride (CPC) is a mono-QAC frequently applied in dental practice, and Octenidine is the only pyridine bis-QAC on the European antiseptic market and is primarily used for skin preparation prior to medical procedures (Figure 1B) [44]. CPC was also found to be effective in viral load reduction and possessed a virucidal effect against SARS-CoV-2 [45]. Moreover, certain types of QACs can prevent growth and disrupt bacterial and fungi biofilms [41]. The pyridinium core in the QAC’s structures serves as a source of positive charge, allowing molecules to enter into electrostatic interaction with negatively charged phospholipid head groups in bacterial membranes, destabilizing the membrane integrity [46,47,48,49].

**Figure 2:**
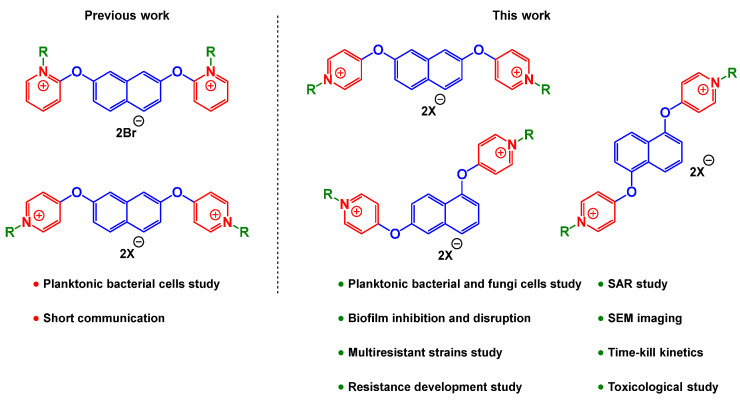
Naphthalene-derivative bis-QACs.

Studies have shown that naphthalene can form efficient hybrid drugs by merging with different biologically active heterocycles, resulting in new molecules with lower toxicity, improved affinity, and better pharmacokinetics [25]. Previously, we conducted a short case study highlighting a possible way to integrate a naphthalene moiety into biologically active pyridinium bis-QACs [50,51]. Herein, we delve deeper into this subject: synthesizing new antibacterials, unraveling structure–activity relationships of para-substituted bis-QACs, and broadening microbiological and toxicological study (Figure 2).

## 2. Results and Discussion

### 2.1. Synthesis

**Figure 3:**
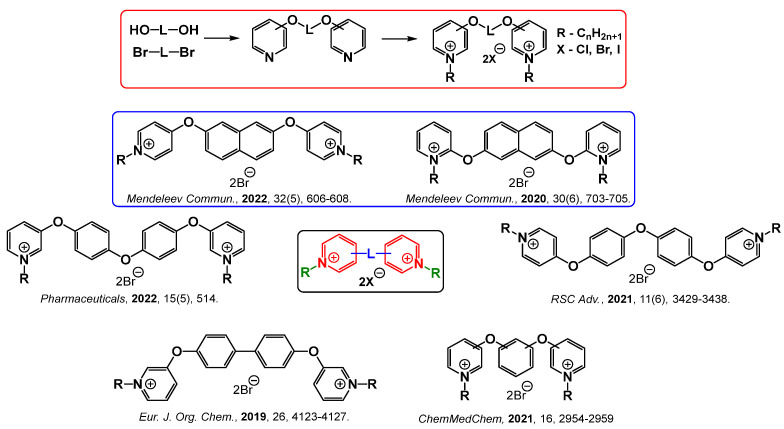
General synthesis strategy towards pyridinium bis-QACs with aromatic linker [50,51,52,53,54,55].

The preparation of bis-QACs was achieved by using the synthesis model previously developed in our laboratory [52,53,54]. This two-step approach involves the assembly of a “core-linker-core” platform via an Ullmann-type coupling reaction between dibromo- or dihydroxy-aromatic linkers and halo- or hydroxypyridines, followed by *N*-alkylation of the resulting bis-pyridine scaffolds with alkyl halides (“tail” fragment) using the Menshutkin reaction (Figure 3). The presented model has proven its effectiveness in many cases and has paved the way for the development of more than 100 antibacterial agents.

**Scheme 1:**
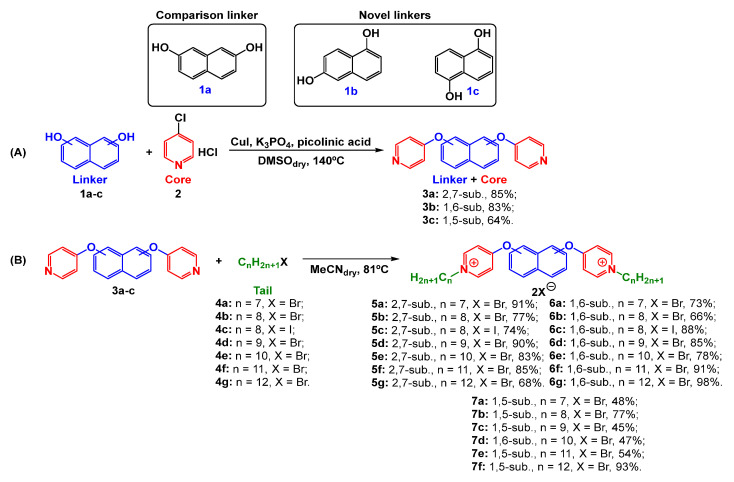
Synthesis of naphthalene-derivative bis-QACs: (**A**) Ullmann-type coupling reaction of dihydroxynaphthalenes and 4-chloropyridine hydrochloride; (**B**) N-alkylation via Menshutkin reaction.

For the current work, dihydroxynaphthalenes were selected as the aromatic linker according to the results of past short case studies [50,51]. In addition to the 2,7-dihydroxynaphthalene used previously, the series was expanded with 1,6- and 1,5-dihydroxynaphthalene (Scheme 1). Therefore, compounds **3a** and **5a**–**g** were reproduced for comparison purposes and to broaden the study of microbiological properties, and compounds **3b**–**c**, **6a**–**g,** and **7a**–**f** are novel.

The first stage was performed with 4-chloropyridine hydrochloride in the presence of copper (I) iodide, potassium phosphate, and picolinic acid in dry DMSO under an argon atmosphere. The reaction showed good yields and repeatability, consistent with our previous results. It is worth noting that any other halopyridines can be used in the reaction (Scheme 1A).

For the second stage, alkyl bromides were mainly used as the most convenient option for the quaternization of pyridine cores. The reaction can take place in polar solvents such as acetonitrile, butanol-1, methyl isobutyl ketone, dimethylformamide, dimethylacetamide, dimethyl sulfoxide, etc., when heated to 50–120 °C. Herein, dry acetonitrile was used as a solvent and alkylation proceeded for 24 h under reflux (Scheme 1B). It is worth noting that ortho-substituted analogue (as pictured in Figure 3) requires much higher energy inputs during *N*-alkylation. Reaction usually proceeds with lower yields and poor repeatability, which makes it less cost effective compared to both para- and meta-substituted pyridine scaffolds.

The chemical structures of novel bis-QACs were identified by NMR, IR, and HRMS spectra. Detailed descriptions of the newly synthesized compounds can be found in experimental protocols and Supporting Materials.

### 2.2. Antimicrobial Activity

The antimicrobial activities of all synthesized QACs were evaluated on ten strains from the ESKAPE group, which consists of highly virulent bacterial pathogens and is one of the leading causes of nosocomial life-threatening infections worldwide [56,57].

**Figure 4:**
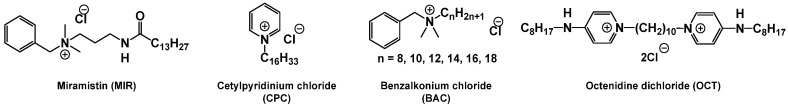
Commercial QACs used as reference compounds in antimicrobial studies.

The pathogens studied were divided into two groups: reference strains and clinical isolates. Reference strains are a standard panel of ATCC cell line microorganisms from the ESKAPE group, which goes as follows: *E. coli* ATCC 25922, *K. pneumoniae* ATCC 70060, *S. aureus* ATCC 43300, *A. baumannii* ATCC 15308, and *P. aeruginosa* ATCC 27853. Clinical isolates are multidrug-resistant bacterial strains that were isolated in cases of infection in hospitalized patients, which includes *E. coli* B-3421/19, *K. pneumoniae* B-2523/18, *S. aureus* B-8648, *A. baumannii* B-2926/18, and *P. aeruginosa* B-2099/18. Commercial mono-QACs miramistin (MIR), CPC, benzalkonium chloride (BAC), and gemini-QAC octenidine dichloride (OCT) were applied as the reference compounds (Figure 4). Activity was assessed by minimum inhibitory concentration (MIC) and minimum bactericidal concentration (MBC).

The microbiological assay showed that the synthesized bis-QACs have a wide spectrum of antibacterial activity (Table 1 and Table S1). The leading compounds were superior in their bacteriostatic and bactericidal action to commercial mono-QACs and were comparable to the best bis-QAC on the market. Further, we will review some emerging structure–activity relationship (SAR) trends for naphthalene-derived compounds so far.

The MICs and MBCs of bis-QACs on Gram-positive *S. aureus* were maintained on a relatively high level within one to two dilutions in the range from C7 to C10 alkyl for the 2,7 and 1,5-substituted linker (compounds **5a**–**e** and **7a**–**d**) and from C7 to C9 for the 1,6-substituted linker (compounds **6a**–**d**). As the alkyl tail increased to C11, activity decreased and then dropped sharply when lengthening to C12. This effect is called the «cut-off» and was described previously [58,59,60]. A similar pattern was observed in clinical isolates of *S. aureus*. In the case of *E. coli*, the optimal range of alkyl tails narrowed to C8–C10, followed by a sharp activity drop. For clinical strains, this gap was even shorter, and the activity decreased for bis-QACs with C10 alkyl (compounds **5e** and **6e**) with the exception of series **7**, where compound **7d** retained antibacterial potential.

For *K. pneumoniae*, *P. aeruginosa,* and *A. baumannii* strains, the minimum concentrations of substances to exhibit activity increased at least 4-fold with *P. aeruginosa* being the most resilient, which is consistent with previously observed tendencies. It is worth noting that, unlike other pathogens, the best result of bactericidal activity against *A. baumannii* ATCC 15308 begins to appear only in C10 alkyl. Moreover, its level remained high for series **5** and **6,** up to C12 alkyl (compounds **5e**–**g** and **6e**–**g**).

Certain deviations were noted when studying the antibacterial action of obtained bis-QACs on *P. aeruginosa*. Series **6** showed a nonlinear relationship with activity peaks at C7, C9, and C11 alkyl (compounds **6a**, **6d,** and **6f**, respectively) against the reference strain. Compound **5a** unexpectedly showed the best results on *P. aeruginosa* B-2099/18, while for other series the optimum activity was achieved only at C9-C10 tail length. Thus, in series **5**, a gradual regression of the antibacterial effect on this pathogen was observed with an increase in the tail fragment.

The counterion did not significantly affect the activity when comparing compounds **5b**/**5c** and **6b**/**6c**. MIC and MBC levels were within the one dilution range, with a couple exceptions on individual pathogens. The potencies of iodides **5c** and **6c** were four times higher than the corresponding bromides against *A. baumannii* B-2926/18. However, this was not the case for the corresponding reference strain *A. baumannii* ATCC 15308. Thus, the data obtained are insufficient for an unambiguous conclusion about the influence of iodide ions on the overall activity of QACs.

### 2.3. Antibiofilm Activity

The antibiofilm properties of all synthesized QACs were evaluated on the same ESKAPE pathogens. Activity was assessed by minimum biofilm inhibition concentration (MBIC) and minimum biofilm eradication concentration (MBEC) (Table 2).

Usually, much higher concentrations of compounds are required to inhibit or eradicate biofilms. This can be explained by the unique mechanisms of adaptation and protection of bacterial cells in a self-produced matrix (protective barrier function, altered metabolism, gene expression, protein production, synergistic bacterial interactions, etc.), which significantly complicate antibacterial therapy [61,62].

However, in the case of the obtained bis-QACs, non-typical patterns were observed for the *S. aureus* and *E. coli* strains. Compounds **5e** and **5f** demonstrated 4 to 16-fold greater performance against reference and clinical *S. aureus* biofilms compared to planktonic cells. Despite the fact that *S. aureus* is usually found more susceptible to antibacterial effects in correlation with Gram-negative strains, the observed MBIC and MBEC spike is not standard and is encountered in our practice for the first time. Moreover, bis-QAC **6e** showed more prominent biofilm eradication properties on the *E. coli* isolate. Depending on the chosen methodology for microbiological analysis of biofilms, the results of antimicrobial activity may vary [63]. Additionally, the results can be influenced by factors such as the stage of biofilm development, cultivation method, mechanism of antibacterial action, etc., which makes biofilm research more complex and not always consistent. Thus, this phenomenon requires further study on a larger QAC panel and different biofilm cultures. The antibiofilm activities of compounds **5**–**7** against *K. pneumoniae*, *P. aeruginosa,* and *A. baumannii* were much lower but within the range of one or two dilutions from the best of the reference compounds. These observations support the theory that the best strategy for combating Gram-negative biofilms is to prevent their formation on surfaces, rather than eradicate existing ones, which is achieved either by preliminary treatment of surfaces with antibacterial formulations or creating antibacterial coatings and materials, including QAC-embedded materials [41].

While the counterion effect against planktonic cells was inconsistent and mostly insignificant, iodides possessed greater activity on both strains of *E. coli* biofilms compared to corresponding bromides. Moreover, compound **5c** showed the lowest MBIC and MBEC against this pathogen among the entire panel, including commercial QACs, and was also more active against *S. aureus* when set side by side with **5b**. Unfortunately, there are very few studies concerning the influence of counterions on the antibiofilm action of QACs, which makes it difficult to draw a clear conclusion. The most accepted current opinion is that the type of counterion is not as significant for the antimicrobial activity as the alkyl chain length, which also correlates with our studies [35,64].

### 2.4. Additional Microbiological Assay

Overall, compounds **5d** and **6d** demonstrated broader scope of activity and were chosen for further microbiological assay on an additional five multi-resistant ESKAPE and fungi strains, which acquired their properties during COVID-19 (Table 3). OCT, CPC, and MIR were used as reference compounds.

CPC showed poorer efficacy on planktonic cells and biofilms, with the exception of *S. aureus* MRSA 0576. The remaining results indicated that the QACs’ biological activity held up during the transition to the next generation of resistant microorganisms, with a general retention of minimal effective concentrations. Notably, *P. aeruginosa* C23520/21 biofilms were more susceptible to antibacterial agents compared to *P. aeruginosa* ATCC 27853 and *P. aeruginosa* B-2099/18. Thus, MBEC for **5d** and **6d** decreased from >500 mg/L to 63 mg/L. The bis-QACs **5d** and **6d** also showed good antifungal activity against *C. auris* F-2035, where MFC and MBEC were at a comparable level, and promising inhibition properties against *C. auris* KA9. *C. auris* is an emerging fungus that can cause severe, often multidrug-resistant infections and spread easily among patients, resulting in large hospital outbreaks [65,66].

### 2.5. Lipophilicity–Activity Relationship and Solubility Study

**Figure 5:**
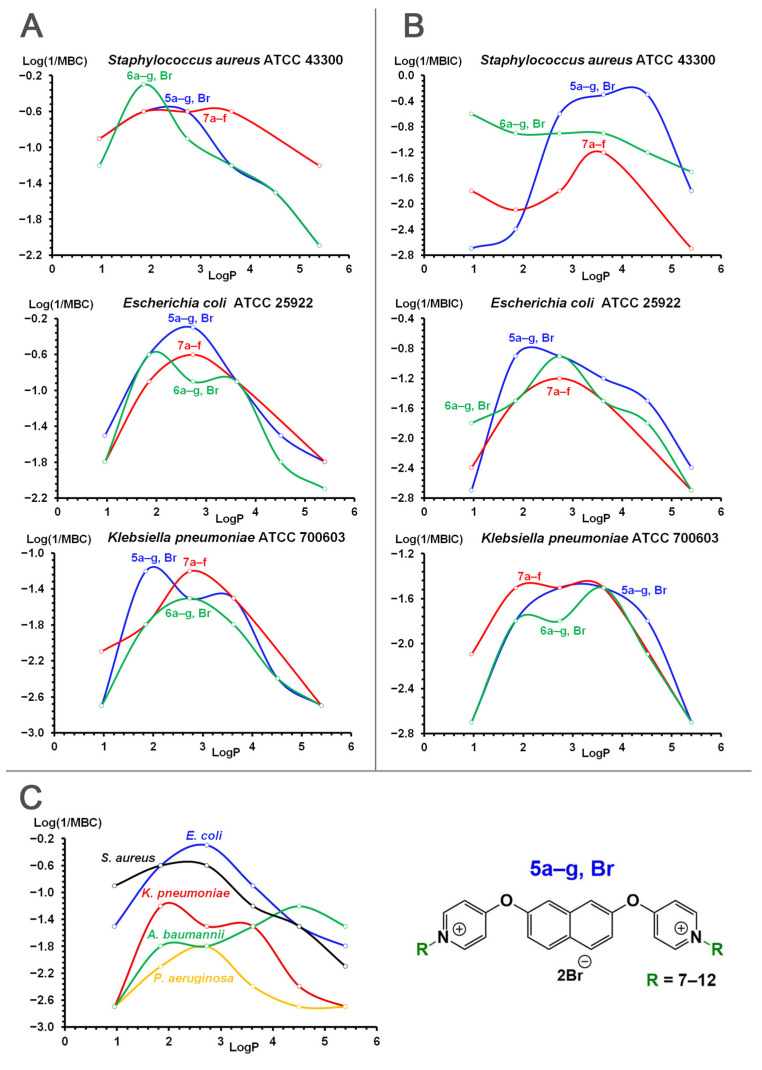
Lipophilicity–activity relationship of QACs. LogP values were calculated using the online platform Chemicalize. (**A**) The relationship between the calculated LogP and the bactericidal activity (LogMBC^−1^) of bis-QACs **5**–**7** against planktonic Gram-positive *S. aureus*, *E. coli,* and *K. pneumoniae*; (**B**) the relationship between the calculated LogP and the biofilm inhibition activity (LogMBIC^−1^) of bis-QACs **5**–**7** against Gram-positive *S. aureus*, *E. coli,* and *K. pneumoniae* biofilms; (**C**) the relationship between the calculated LogP and the bactericidal activity (LogMBC^−1^) of bis-QACs **5a**–**g** against all reference strains.

A clearer picture of the effect of linker conformation on antibacterial activity is shown in lipophilicity–activity relationship plots (Figure 5). Lipophilicity is one of the key physicochemical properties for the microbiological analysis of drugs, including antibacterials. The lipophilic character, quantified by the logarithmic partition coefficient (LogP), determines a compound’s ability to penetrate through the lipid bacterial membrane [67,68,69].

To display the structure/activity correlation, we overlaid the LogP/activity plots of series **5**–**7** for *S. aureus*, *E. coli,* and *K. pneumoniae* reference planktonic cells (Figure 5A) and biofilms (Figure 5B). In light of the final picture, differences in linker conformation do not significantly affect the activity, and it is difficult to identify an overall clear advantage. Therefore, no strong gaps were noticed between the linkers on planktonic cells, with the exception of a slight advantage of series **7** after LogP > 2.7 on *S. aureus* and a larger peak of activity for series **5** at LogP = 2.7 on *E. coli*. For antibiofilm activity bis-QACs **5** were noticeably superior in 2.7–5.2 LogP interval against *S. aureus* and slightly better in 1.4–5.4 LogP interval against *E. coli* (Figure 5B). Despite this, it can be theorized that symmetry has a partial influence on antibacterial performance. For example, symmetrical bis-QACs **5** and **7** have a slight advantage compared to the asymmetrical **6** on planktonic *E. coli* when LogP > 2.4 and on *K. pneumoniae* in the 0.9–5.4 LogP interval. On the other hand, series **6** exhibits better antibiofilm properties compared to the symmetrical **7**, which adds complexity to this theory.

The impaired activity of **7** against biofilms could be due to their poor solubility in water, which is at least 10 times lower compared to the corresponding bis-QACs **5**–**6** (Table 4). Upon reaching the C11 tail, bis-QACs **7** become almost insoluble in standard conditions, which greatly complicates microbiological studies. On the other hand, iodides **5c** and **6c** showed greater antibiofilm potential than bromide counterparts, while being 7-fold less soluble. The literature data concerning the lower solubility/lower activity correlation are also controversial. Some studies found that better water solubility can spike bioavailability and subsequently improve antibacterial and antibiofilm action [59,70,71,72]. In contrast, the results of chitosan and chitooligomers research indicated that water-soluble compounds do not exhibit significant antimicrobial activity, while the water-insoluble forms showed better antibacterial effects [73]. Summarizing, the solubility of compounds in water certainly simplifies both the use of active substances and their microbiological and physicochemical studies. Nonetheless, this factor does not necessarily correlate directly with antibacterial activity, as shown in this and other works. It is possible that there is a certain solubility limit (close to completely insoluble forms), after which the compounds either lose their activity against microorganisms and biofilms or their qualitative determination becomes impossible. However, these studies go beyond the scope of the presented work.

It is worth noting that compounds **5b**–**e** and **6b**–**e**, which belong to the range of the most active bis-QACs in the corresponding series, demonstrated similar or higher water solubility compared to OCT, which gives them an advantage for possible further integration as commercial disinfectants or antiseptics.

### 2.6. Time–Kill Kinetics

**Figure 6:**
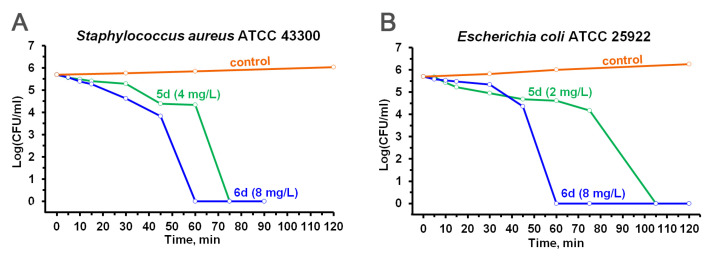
Time–kill kinetics of bis-QACs **5d**, **6d,** and control against: (**A**) *S. aureus* ATCC 43300; (**B**) *E. coli* ATCC 25922.

To further explore the antimicrobial potential of bis-QACs against Gram-positive and Gram-negative bacteria, a time–kill kinetics assay was conducted using *S. aureus* ATCC 43300 and *E. coli* ATCC 25922. The concentration of the substances corresponded to the MBC, and the bacterial mixture was collected at different time points for a colony count assay. The untreated strains were used as the control (Figure 6).

The assay showed that compounds **5d** at MBC 4 mg/L and **6d** at MBC 8 mg/L could completely kill the colony of *S. aureus* ATCC 43300 within 60 min. Gemini-QAC **6d** also eradicated E. *coli* ATCC 25922 in 60 min, and **5d** at MBC 2 mg/L achieved the same results within 105 min. Overall, a gradual decrease in CFU was observed, which is suitable for use as biocidal agents for surface and instrument disinfection.

### 2.7. Bacterial Resistance Formation

Bacterial resistance has been a centuries-old, global problem since the discovery of antibiotics. However, the mechanism of its development still remains a source of debate and speculation among the scientific community, including the resistance to QACs and co-resistance to QACs and antibiotics. Although some studies have found a correlation between QAC tolerance and antibiotic resistance, there is currently insufficient evidence to conclude that the widespread use of QACs has generally contributed to the emergence of antibiotic resistance [74,75,76].

**Figure 7:**
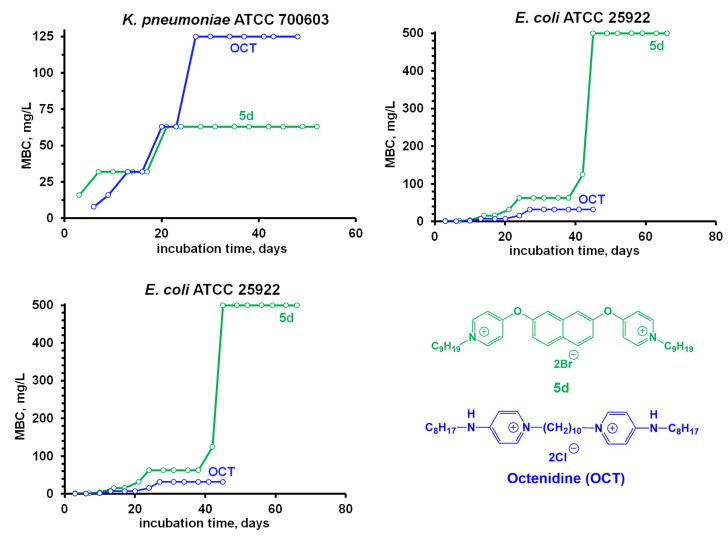
Bacterial resistance study of **5d** and OCT on ESKAPE pathogens.

In a previous study, we noticed that for pyridinium QACs with aromatic linkers, the development of resistance in bacteria was absent or slowed down [55]. Thus, the tendencies of resistance development of aromatic QACs **5d** against ESKAPE pathogens were further carried out. Commercial OCT was used as a reference (Figure 7).

Under conditions of stepwise increasing concentrations of gemini-QAC **5d** (1–500 mg/L) for 44 days (12 passages), it was not possible to obtain a resistant version of the *S. aureus* ATCC 43300. The MBC against the obtained strain did not change and was retained on 4 mg/L. Notably, a similar situation was observed for the *K. pneumoniae* ATCC 70060, where over 66 days, the MBC increased by only one dilution. However, *E. coli* ATCC 25922 acquired an extremely high level of resistance to the **5d**. The MBC against the resulting strain increased 250 times relative to the original and were equal to 500 mg/L. Nevertheless, the results obtained give us hope that the aromatic moiety may indeed reduce the ability of certain microorganisms to acquire resistance, which requires further research.

### 2.8. SEM Imaging

**Figure 8:**
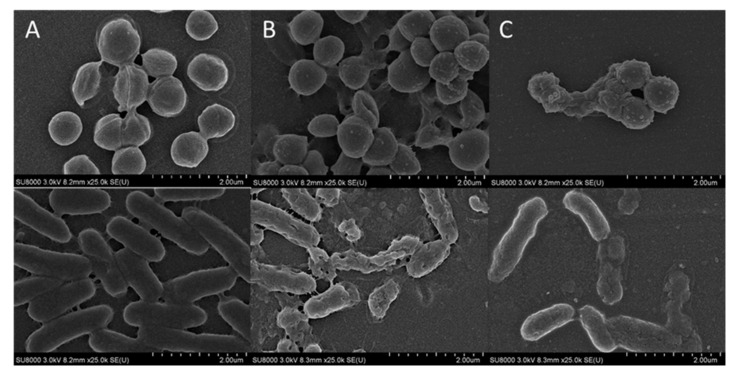
SEM images of *S. aureus* ATCC 43300 (**first row**) and *P. aeruginosa* ATCC 27853 (**second row**) biofilms: (**A**) untreated; (**B**) after exposure to **5d**; (**C**) after exposure to **6d**.

Scanning electron microscopy (SEM) was used to visualize the morphology and deviations in the cellular structure of bacteria after exposure to QACs. SEM is a useful tool to measure key morphological parameters of bacterial cells that can provide insights into bacterial growth phase, nutritional conditions, and adaptations to the external factors, including antibacterials action [77]. Herein, SEM imaging was performed on Gram-positive *S. aureus* ATCC 43300 and Gram-negative *P. aeruginosa* ATCC 27853 biofilms before and after exposure to **5d** and **6d** (Figure 8). We also conducted a study in which we used SEM to analyze changes in bacterial morphology after different exposure times to our bis-QACs (Figure S2).

As shown in the picture, untreated bacteria had a smooth cell surface and an organized, uniform structure (Figure 8A). While after the exposure obvious membrane irregularities, wrinkles, and severe cell deformations were noted (Figure 8B,C). Remarkably, distinguishable holes in the membrane and disrupted cells were observed in the *P. aeruginosa* strain. The presented results support the theory of the membrane-destructive action of novel bis-QACs on Gram-positive and Gram-negative pathogens of the ESKAPE group.

### 2.9. MTT and Hemolysis Assay

Further bis-QACs **5**–**6** were subjected to a toxicological assay on mammalian cells using human embryonic kidney cells 293T (HEK-293T) and human red blood cells (hRBCs). Cytotoxicity and hemolytic activity were assessed by half-maximal concentrations (CC_50_ and HC_50_) and selectivity indices compare to MIC (SI_MIC_) and MBC (SI_MBC_) against reference planktonic strains (Table 5 and Table 6) and other pathogens (Tables S1–S6). SI above 1 represents compounds with antimicrobial activity higher than its toxic effect both towards HEK-293T and hRBCs. Commercial mono-QACs MIR, CPC, BAC, and gemini-QAC OCT were applied as the reference compounds.

**Figure 9:**
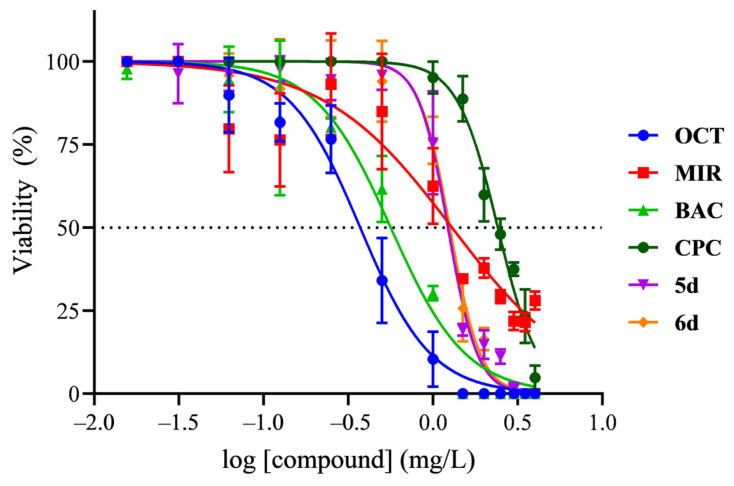
Cell-viability of HEK-293T exposed to QACs.

**Figure 10:**
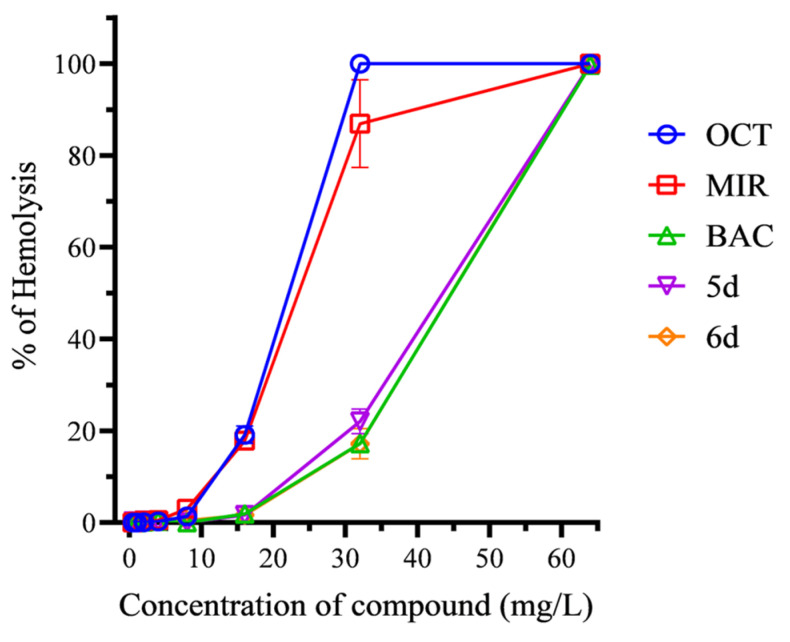
Hemolytic effect of hRBCs exposed to QACs.

Experimental data showed that naphthalene bis-QACs have lower toxicity than widely used antiseptics OCT and BAC and were comparable to MIR and CPC. Linker structure did not affect toxicological properties, thereby CC_50_, HC_50_, cell-viability, and hemolysis plots were almost identical for **5d** and **6d** (Figure 9 and Figure 10).

As the alkyl chain length increased, the toxicity of bis-QACs decreased significantly. Substance **5f** also turned out to be selective towards Gram-negative bacteria. (Table 5 and Table S2). For other pathogens SIs were significantly lower than 1.00, especially for *K. pneumoniae*, *P. aeruginosa,* and *A. baumannii*. As expected, selectivity further decreased when targeting biofilms (Tables S3 and S4). In contrast, selectivity indices improved significantly for all bis-QACs when their hemolytic activity was examined. Thus, compound **5d** and **6d** showed an HC_50_ higher than the corresponding MIC values for five pathogen strains, indicating good selectivity (Table 6).

## 3. Materials and Methods

### 3.1. Chemistry

#### 3.1.1. General Information

All starting reagents were obtained from commercial suppliers and were used without further purification. All solvents used were reagent grade and distilled prior to application. Air sensitive reactions were performed under a high-purity argon atmosphere. Thin-layer chromatography was performed using silica gel 60 F254 aluminum sheets under UV light. IR spectra were recorded with a Bruker ALPHA-T FT-IR spectrometer in KBr pellets. HR-ESI-MS were recorded on a Bruker microTOF II instrument (Bruker Daltonik GmbH, Bremen, Germany); external or internal calibration was performed with electrospray calibrant solution (Fluka). All melting points were measured with a Stuart SMP30 melting point apparatus and are uncorrected. 1H and 13C NMR spectra were recorded with a Bruker AM300 at ambient temperature in Dimethyl sulfoxide-*d*₆ (DMSO-*d*_6_) or Chloroform-d (CDCl_3_) solutions. The NMR, HRMS, and FT-IR spectra of the newly synthesized compounds can be found in the Supporting Information.

#### 3.1.2. Reference Compounds

The compounds **5a**–**g** were reproduced for comparison with the new series of bis-QACs and expansion of microbiological activity scope, including antibiofilm assay, analysis against additional ESKAPE pathogens, antifungal activity, cytotoxicity assay, bacterial resistance, etc. Full experimental and characterization data for **3a** and **5a**–**g** can be found elsewhere [51]. CPC was synthesized from pyridine using the widely known method of N-alkylation by cetylchloride in dry acetonitrile. OCT was synthesized using a previously described method [78]. BAC was purchased from Acros organics and used without further purification. MIR was synthesized using a previously described method [79].

#### 3.1.3. Synthesis Procedure and Characterization of Bis-Pyridine Scaffolds (**3b**–**c**)

The mixture of dihydroxynaphtalene (1.28 g, 8 mmol), 4-chloropyridine hydrochloride (2.4 g, 16 mmol), potassium phosphate (6.79 g, 32 mmol), copper (I) iodide (1.52 g, 8 mmol), and picolinic acid (0.19 g, 1.6 mmol) in freshly distilled DMSO (50 mL) was heated at 140 °C for 48 h in a high-purity argon atmosphere. After solvent evaporation under reduced pressure, ethyl acetate (100 mL) was added to reaction residue, and the mixture was heated to reflux for 1 h. The resulting suspension was allowed to cool to room temperature and then was filtered through a filter paper. The solid remaining on the filter paper was washed three times with 20 mL of ethyl acetate. The reflux–filtration–washing procedure was repeated twice with the remaining precipitate. The solvent from combined organic filtrates was removed under reduced pressure and the crude residue was purified by recrystallization in heptane to provide a solid product.

*1,6-Bis(pyridin-4-yloxy)naphthalene* (**3b**). White solid (2.09 g, 6.64 mmol, 83% yield). C_20_H_14_N_2_O_2_, M = 314.34, M.p. = 92–95 °C; ^1^H NMR (300 MHz, DMSO-*d*_6_) δ 8.5 (br s, 4H, 4CH_Pyr_), 7.97 (d, *J* = 9.1 Hz, 1H, 1CH_Ar_), 7.90 (d, *J* = 7.9 Hz, 1H, 1CH_Ar_), 7.84 (d, *J* = 2.3 Hz, 1H, 1CH_Ar_), 7.63 (t, *J* = 7.9 Hz, 1H, 1CH_Ar_), 7.40 (dd, *J* = 9.1 Hz, 2.3 Hz, 1H, 1CH_Ar_), 7.33 (d, *J* = 7.9 Hz, 1H, 1CH_Ar_), 7.03 (d, *J* = 5.5 Hz, 2H, 2CH_Pyr_), 6.97 (d, *J* = 5.5 Hz, 2H, 2CH_Pyr_); ^13^C NMR (75 MHz, DMSO-*d*_6_) δ 164.8, 164.0, 152.8, 152.1, 149.8, 136.4, 127.9, 125.7, 124.5, 124.4, 121.7, 117.6, 117.1.

*1,5-Bis(pyridin-4-yloxy)naphthalene* (**3c**). Bright yellow solid (1.61 g, 5.12 mmol, 64% yield). C_20_H_14_N_2_O_2_, M = 314.34, M.p. = 211–214 °C; IR (neat/cm^−1^): 3449, 1712, 1585, 1490, 1404, 1265, 1204, 946, 798, 517; ^1^H NMR (300 MHz, DMSO-*d*_6_) δ 8.18 (d, *J* = 7.4 Hz, 4H, 4CH_Pyr_), 7.82 (d, *J* = 7.7 Hz, 2H, 2CH_Ar_), 7.62 (t, *J* = 7.7 Hz, 2H, 2CH_Ar_), 7.43 (d, *J* = 7.7 Hz, 2H, 2CH_Ar_), 6.30 (d, *J* = 7.4 Hz, 4H, 4CH_Pyr_); ^13^C NMR (75 MHz, DMSO-*d*_6_) δ 164.8, 152.1, 150.0, 128.6, 127.7, 119.4, 118.4, 112.5.

#### 3.1.4. General Synthesis Procedure and Characterization of Bis-QACs (**6a**–**g**, **7a**–**f**)

Halogenoalkane (4 mmol) was added to a solution of bis-pyridine scaffold (1 mmol, 0.314 g) in dry acetonitrile (3 mL). The mixture was stirred and heated to reflux for 24 h. After solvent evaporation under reduced pressure, ice cold ethyl acetate (5 mL) was added to the reaction residue and the mixture was vigorously stirred for 3 min. Formed precipitates were quickly filtrated through a glass filter, washed with ice cold ethyl acetate (20 mL), and dried under a vacuum. Bis-QACs were obtained as white (when bromoalkanes were used) or yellow (when iodoalkanes were used) solids.

*4,4′-(Naphthalene-1,6-diylbis(oxy))bis(1-heptylpyridin-1-ium)dibromide* (**6a**). White solid (0.49 g, 0.73 mmol, 73% yield). C_34_H_44_Br_2_N_2_O_2_, M = 672.55, M.p. = 196–199 °C, IR (neat/cm^−1^): 3446, 2930, 2858, 1720, 1643, 1514, 1479, 1300, 1178, 796; ^1^H NMR (300 MHz, DMSO-*d*_6_) δ 8.99 (d, *J* = 6.7 Hz, 4H, 4CH_Pyr_), 8.17 (d, *J* = 1.8 Hz, 1H, 1CH_Ar_), 8.11 (d, *J* = 8.0 Hz, 1H, 1CH_Ar_), 8.08 (d, *J* = 9.0 Hz, 1H, 1CH_Ar_), 7.80 (t, *J* = 8.0 Hz, 1H, 1CH_Ar_), 7.72–7.63 (m, 5H, 4CH_Pyr_,1CH_Ar_), 7.60 (dd, *J* = 9.0 Hz, 1.8 Hz, 1H, 1CH_Ar_), 4.51 (t, *J* = 7.0 Hz, 4H, 2N^+^CH_2_), 2.03–1.74 (m, 4H, 2CH_2_), 1.45–1.12 (m, 16H, 8CH_2_), 0.86 (t, *J* = 6.3 Hz, 6H, 2CH_3_); ^13^C NMR (75 MHz, DMSO-*d*_6_) δ 169.8, 169.4, 151.3, 148.3, 147.6, 147.5, 136.4, 128.4, 127.9, 124.9, 124.6, 122.0, 119.5, 118.8, 115.5, 115.3, 59.6, 31.5, 31.1, 28.6, 25.8, 22.5, 14.4; HRMS (ESI-TOF): [M]^2+^ calcd for C_34_H_44_N_2_O_2_ 256.1696, found 256.1705.

*4,4′-(Naphthalene-1,6-diylbis(oxy))bis(1-octylpyridin-1-ium)dibromide* (**6b**). White solid (0.46 g, 0.66 mmol, 66% yield). C_36_H_48_Br_2_N_2_O_2_, M = 700.6, M.p. = 217–220 °C, IR (neat/cm^−1^): 3446, 2927, 2856, 1643, 1514, 1478, 1300, 1207, 1178, 795; ^1^H NMR (300 MHz, DMSO-*d*_6_) δ 8.98 (d, *J* = 6.8 Hz, 4H, 4CH_Pyr_), 8.17 (d, *J* = 1.8 Hz, 1H, 1CH_Ar_), 8.11 (d, *J* = 8.0 Hz, 1H, 1CH_Ar_), 8.08 (d, *J* = 9.0 Hz, 1H, 1CH_Ar_), 7.80 (t, *J* = 8.0 Hz, 1H, 1CH_Ar_), 7.72–7.63 (m, 5H, 4CH_Pyr_, 1CH_Ar_), 7.60 (dd, *J* = 9.0 Hz, 1.8 Hz 1H, 1CH_Ar_), 4.51 (t, *J* = 7.0 Hz, 4H, 2N^+^CH_2_), 2.03–1.74 (m, 4H, 2CH_2_), 1.45–1.12 (m, 16H, 8CH_2_), 0.86 (t, *J* = 6.3 Hz, 6H, 2CH_3_); ^13^C NMR (75 MHz, DMSO-*d*_6_) δ 169.8, 169.4, 151.3, 148.3, 147.6, 147.5, 136.4, 128.4, 127.9, 124.9, 124.6, 122.0, 119.5, 118.8, 115.5, 115.3, 59.6, 31.5, 31.1, 28.6, 25.8, 22.5, 14.4; HRMS (ESI-TOF): [M]^2+^ calcd for C_36_H_48_N_2_O_2_ 270.1852, found 270.1862.

*4,4′-(Naphthalene-1,6-diylbis(oxy))bis(1-octylpyridin-1-ium) Diiodide* (**6c**). White solid (0.69 g, 0.88 mmol, 88% yield). C_36_H_48_I_2_N_2_O_2_, M = 794.60, M.p. = 208–211 °C, IR (neat/cm^−1^): 3469, 2926, 2855, 1642, 1578, 1511, 1480, 1288, 1206, 1179, 794; ^1^H NMR (300 MHz, DMSO-*d*_6_) δ 8.99 (d, *J* = 6.7 Hz, 4H, 4CH_Pyr_), 8.17 (d, *J* = 1.8 Hz, 1H, 1CH_Ar_), 8.11 (d, *J* = 8.0 Hz, 1H, 1CH_Ar_), 8.08 (d, *J* = 9.0 Hz, 1H, 1CH_Ar_), 7.80 (t, *J* = 8.0 Hz, 1H, 1CH_Ar_), 7.72–7.63 (m, 5H, 4CH_Pyr_,1CH_Ar_), 7.60 (dd, *J* = 9.0 Hz, 1.8 Hz 1H, 1CHAr), 4.51 (t, *J* = 7.0 Hz, 4H, 2N^+^CH_2_), 2.03–1.74 (m, 4H, 2CH_2_), 1.45–1.12 (m, 16H, 8CH_2_), 0.86 (t, *J* = 6.3 Hz, 6H, 2CH_3_); ^13^C NMR (75 MHz, DMSO-*d*_6_) δ 169.8, 169.4, 151.3, 148.3, 147.6, 147.5, 136.4, 128.4, 127.9, 124.9, 124.6, 122.0, 119.5, 118.8, 115.5, 115.3, 59.6, 31.5, 31.1, 28.6, 25.8, 22.5, 14.4; HRMS (ESI-TOF): [M]^2+^ calcd for C_36_H_48_N_2_O_2_ 270.1852, found 270.1858.

*4,4′-(Naphthalene-1,6-diylbis(oxy))bis(1-nonylpyridin-1-ium)dibromide* (**6d**). White solid (0.62 g, 0.85 mmol, 85% yield). C_38_H_52_Br_2_N_2_O_2_, M = 728.65, M.p. = 227–230 °C, IR (neat/cm^−1^): 3446, 2925, 2855, 1643, 1578, 1514, 1479, 1301, 1208, 1178, 795; ^1^H NMR (300 MHz, DMSO-*d*_6_) δ 8.99 (d, *J* = 6.7 Hz, 4H, 4CH_Pyr_), 8.17 (d, *J* = 1.8 Hz, 1H, 1CH_Ar_), 8.11 (d, *J* = 8.0 Hz, 1H, 1CH_Ar_), 8.08 (d, *J* = 9.0 Hz, 1H, 1CH_Ar_), 7.80 (t, *J* = 8.0 Hz, 1H, 1CH_Ar_), 7.72–7.63 (m, 5H, 4CH_Pyr_,1CH_Ar_), 7.60 (dd, *J* = 9.0 Hz, 1.8 Hz 1H, 1CHAr), 4.51 (t, *J* = 7.0 Hz, 4H, 2N^+^CH_2_), 2.03–1.74 (m, 4H, 2CH_2_), 1.45–1.12 (m, 16H, 8CH_2_), 0.86 (t, *J* = 6.3 Hz, 6H, 2CH_3_); ^13^C NMR (75 MHz, DMSO-*d*_6_) δ 169.8, 169.4, 151.3, 148.3, 147.6, 147.5, 136.4, 128.4, 127.9, 124.9, 124.6, 122.0, 119.5, 118.8, 115.5, 115.3, 59.6, 31.5, 31.1, 28.6, 25.8, 22.5, 14.4; HRMS (ESI-TOF): [M]^2+^ calcd for C_38_H_52_N_2_O_2_ 284.2009, found 284.2014.

*4,4′-(Naphthalene-1,6-diylbis(oxy))bis(1-decylpyridin-1-ium)dibromide* (**6e**). White solid (0.59 g, 0.78 mmol, 78% yield). C_40_H_56_Br_2_N_2_O_2_, M = 756.71, M.p. = 221–224 °C, IR (neat/cm^−1^): 3446, 2925, 2855, 1643, 1579, 1514, 1479, 1301, 1209, 1179, 796; ^1^H NMR (300 MHz, DMSO-*d*_6_) δ 8.99 (d, *J* = 6.7 Hz, 4H, 4CH_Pyr_), 8.17 (d, *J* = 1.8 Hz, 1H, 1CH_Ar_), 8.11 (d, *J* = 8.0 Hz, 1H, 1CH_Ar_), 8.08 (d, *J* = 9.0 Hz, 1H, 1CH_Ar_), 7.80 (t, *J* = 8.0 Hz, 1H, 1CH_Ar_), 7.72–7.63 (m, 5H, 4CH_Pyr_,1CH_Ar_), 7.60 (dd, *J* = 9.0 Hz, 1.8 Hz 1H, 1CHAr), 4.51 (t, *J* = 7.0 Hz, 4H, 2N^+^CH_2_), 2.03–1.74 (m, 4H, 2CH_2_), 1.45–1.12 (m, 16H, 8CH_2_), 0.86 (t, *J* = 6.3 Hz, 6H, 2CH_3_); ^13^C NMR (75 MHz, DMSO-*d*_6_) δ 169.8, 169.4, 151.3, 148.3, 147.6, 147.5, 136.4, 128.4, 127.9, 124.9, 124.6, 122.0, 119.5, 118.8, 115.5, 115.3, 59.6, 31.5, 31.1, 28.6, 25.8, 22.5, 14.4; HRMS (ESI-TOF): [M]^2+^ calcd for C_40_H_56_N_2_O_2_ 298.2165, found 298.2171.

*4,4′-(Naphthalene-1,6-diylbis(oxy))bis(1-undecylpyridin-1-ium)dibromide* (**6f**). White solid (0.71 g, 0.91 mmol, 91% yield). C_42_H_60_Br_2_N_2_O_2_, M = 784.76, M.p. = 225–228 °C, IR (neat/cm^−1^): 3445, 2925, 2854, 1643, 1578, 1514, 1479, 1299, 1209, 1179, 796; ^1^H NMR (300 MHz, DMSO-*d*_6_) δ 8.99 (d, *J* = 6.7 Hz, 4H, 4CH_Pyr_), 8.17 (d, *J* = 1.8 Hz, 1H, 1CH_Ar_), 8.11 (d, *J* = 8.0 Hz, 1H, 1CH_Ar_), 8.08 (d, *J* = 9.0 Hz, 1H, 1CH_Ar_), 7.80 (t, *J* = 8.0 Hz, 1H, 1CH_Ar_), 7.72–7.63 (m, 5H, 4CH_Pyr_, 1CH_Ar_), 7.60 (dd, *J* = 9.0 Hz, 1.8 Hz 1H, 1CHAr), 4.51 (t, *J* = 7.0 Hz, 4H, 2N^+^CH_2_), 2.03–1.74 (m, 4H, 2CH_2_), 1.45–1.12 (m, 16H, 8CH_2_), 0.86 (t, *J* = 6.3 Hz, 6H, 2CH_3_); ^13^C NMR (75 MHz, DMSO-*d*_6_) δ 169.8, 169.4, 151.3, 148.3, 147.6, 147.5, 136.4, 128.4, 127.9, 124.9, 124.6, 122.0, 119.5, 118.8, 115.5, 115.3, 59.6, 31.5, 31.1, 28.6, 25.8, 22.5, 14.4; HRMS (ESI-TOF): [M]^2+^ calcd for C_42_H_60_N_2_O_2_ 312.2322, found 312.2324.

*4,4′-(Naphthalene-1,6-diylbis(oxy))bis(1-dodecylpyridin-1-ium)dibromide* (**6g**). White solid (0.79 g, 5.28 mmol, 98% yield). C_44_H_64_Br_2_N_2_O_2_, M = 812.82, M.p. = 230–233 °C, IR (neat/cm^−1^): 3444, 2920, 2853, 1644, 1578, 1512, 1468, 1292, 1209, 1179, 860; ^1^H NMR (300 MHz, DMSO-*d*_6_) δ 8.99 (d, *J* = 6.7 Hz, 4H, 4CH_Pyr_), 8.17 (d, *J* = 1.8 Hz, 1H, 1CH_Ar_), 8.11 (d, *J* = 8.0 Hz, 1H, 1CH_Ar_), 8.08 (d, *J* = 9.0 Hz, 1H, 1CH_Ar_), 7.80 (t, *J* = 8.0 Hz, 1H, 1CH_Ar_), 7.72–7.63 (m, 5H, 4CH_Pyr_, 1CH_Ar_), 7.60 (dd, *J* = 9.0 Hz, 1.8 Hz 1H, 1CHAr), 4.51 (t, *J* = 7.0 Hz, 4H, 2N^+^CH_2_), 2.03–1.74 (m, 4H, 2CH_2_), 1.45–1.12 (m, 16H, 8CH_2_), 0.86 (t, *J* = 6.3 Hz, 6H, 2CH_3_); ^13^C NMR (75 MHz, DMSO-*d*_6_) δ 169.8, 169.4, 151.3, 148.3, 147.6, 147.5, 136.4, 128.4, 127.9, 124.9, 124.6, 122.0, 119.5, 118.8, 115.5, 115.3, 59.6, 31.5, 31.1, 28.6, 25.8, 22.5, 14.4; HRMS (ESI-TOF): [M]^2+^ calcd for C_44_H_64_N_2_O_2_ 326.2478, found 326.2482.

*4,4′-(Naphthalene-1,5-diylbis(oxy))bis(1-heptylpyridin-1-ium)dibromide* (**7a**). White solid (0.32 g, 0.48 mmol, 48% yield). C_34_H_44_Br_2_N_2_O_2_, M = 672.55, M.p. = 273–276 °C, IR (neat/cm^−1^): 3436, 2927, 2854, 1640, 1578, 1501, 1478, 1399, 1284, 1172, 942, 814; ^1^H NMR (300 MHz, DMSO-*d*_6_) δ 8.99 (d, *J* = 7.3 Hz, 4H, 4CH_Pyr_), 7.94 (d, *J* = 7.9 Hz, 2H, 2CH_Ar_), 7.76 (t, *J* = 7.9 Hz, 2H, 2CH_Ar_), 7.72 (d, *J* = 7.9 Hz, 2H, 2CH_Ar_), 7.67 (d, *J* = 7.3 Hz, 4H, 4CH_Pyr_), 4.51 (t, *J* = 7.3 Hz, 4H, 2N^+^CH_2_), 1.94–1.79 (m, 4H, 2CH_2_), 1.37–1.17 (m, 16H, 8CH_2_), 0.86 (t, *J* = 6.7 Hz, 6H, 2CH_3_); ^13^C NMR (75 MHz, DMSO-*d*_6_) δ 169.8, 148.6, 147.6, 128.5, 127.9, 120.7, 119.8, 115.4, 59.7, 31.5, 31.1, 28.5, 25.8, 22.4, 14.4; HRMS (ESI-TOF): [M]^2+^ calcd for C_34_H_44_N_2_O_2_ 256.1696, found 256.1705.

4*,4′-(Naphthalene-1,5-diylbis(oxy))bis(1-octylpyridin-1-ium)dibromide* (**7b**). White solid (0.54 g, 0.77 mmol, 77% yield). C_36_H_48_Br_2_N_2_O_2_, M = 700.6, M.p. = 284–287 °C, IR (neat/cm^−1^): 3436, 2928, 2854, 1640, 1578, 1500, 1477, 1399, 1283, 1170, 942, 812; ^1^H NMR (300 MHz, DMSO-*d*_6_) δ 9.00 (d, *J* = 7.4 Hz, 4H, 4CH_Pyr_), 7.95 (d, *J* = 8.0 Hz, 2H, 2CH_Ar_), 7.77 (t, *J* = 8.0 Hz, 2H, 2CH_Ar_), 7.72 (dd, *J* = 8.0 Hz, 1.4 Hz 2H, 2CH_Ar_), 7.68 (d, *J* = 7.4 Hz, 4H, 4CH_Pyr_), 4.51 (t, *J* = 7.3 Hz, 4H, 2N^+^CH_2_), 1.99–1.76 (m, 4H, 2CH_2_), 1.38–1.17 (m, 20H, 10CH_2_), 0.86 (t, *J* = 6.6 Hz, 6H, 2CH_3_); ^13^C NMR (75 MHz, DMSO-*d*_6_) δ 169.8, 148.6, 147.6, 128.5, 127.9, 120.7, 119.8, 115.4, 59.7, 31.6, 31.1, 28.9, 28.8, 25.8, 22.5, 14.4; HRMS (ESI-TOF): [M]^2+^ calcd for C_36_H_48_N_2_O_2_ 270.1852, found 270.1862.

*4,4′-(Naphthalene-1,5-diylbis(oxy))bis(1-nonylpyridin-1-ium)dibromide* (**7c**). White solid (0.33 g, 0.45 mmol, 45% yield). C_38_H_52_Br_2_N_2_O_2_, M = 728.65, M.p. = 240–243 °C, IR (neat/cm^−1^): 3436, 2924, 2851, 1640, 1578, 1496, 1398, 1285, 1176, 942, 804; ^1^H NMR (300 MHz, DMSO-*d*_6_) δ 8.97 (d, *J* = 7.4 Hz, 4H, 4CH_Pyr_), 7.95 (d, *J* = 7.8 Hz, 2H, 2CH_Ar_), 7.76 (t, *J* = 7.8 Hz, 2H, 2CH_Ar_), 7.71 (dd, *J* = 7.8 Hz, 1.3 Hz 2H, 2CH_Ar_), 7.67 (d, *J* = 7.4 Hz, 4H, 4CH_Pyr_), 4.50 (t, *J* = 7.3 Hz, 4H, 2N^+^CH_2_), 1.97–1.80 (m, 4H, 2CH_2_), 1.40–1.17 (m, 24H, 12CH_2_), 0.86 (t, *J* = 6.6 Hz, 6H, 2CH_3_); ^13^C NMR (75 MHz, DMSO-*d*_6_) δ 169.8, 148.6, 147.5, 128.5, 127.9, 120.7, 119.7, 115.4, 59.8, 31.7, 31.1, 29.2, 29.0, 28.9, 25.8, 22.5, 14.4; HRMS (ESI-TOF): [M]^2+^ calcd for C_38_H_52_N_2_O_2_ 284.2009, found 284.2016.

*4,4′-(Naphthalene-1,5-diylbis(oxy))bis(1-decylpyridin-1-ium)dibromide* (**7d**). White solid (0.36 g, 0.47 mmol, 47% yield). C_40_H_56_Br_2_N_2_O_2_, M = 756.71, M.p. = 287–290 °C, IR (neat/cm^−1^): 3436, 2924, 2850, 1640, 1580, 1496, 1397, 1285, 1176, 943, 804; ^1^H NMR (300 MHz, DMSO-*d*_6_) δ 8.97 (d, *J* = 7.1 Hz, 4H, 4CH_Pyr_), 7.95 (d, *J* = 7.8 Hz, 2H, 2CH_Ar_), 7.76 (t, *J* = 7.7 Hz, 2H, 2CH_Ar_), 7.71 (dd, *J* = 7.7 Hz, 1.2 Hz 2H, 2CH_Ar_), 7.67 (d, *J* = 7.1 Hz, 4H, 4CH_Pyr_), 4.50 (t, *J* = 7.1 Hz, 4H, 2N^+^CH_2_), 1.96–1.79 (m, 4H, 2CH_2_), 1.37–1.17 (m, 28H, 14CH_2_), 0.86 (t, *J* = 6.6 Hz, 6H, 2CH_3_); ^13^C NMR (75 MHz, DMSO-*d*_6_) δ 169.8, 148.6, 147.5, 128.5, 127.9, 120.7, 119.7, 115.4, 59.8, 31.7, 31.0, 29.3, 29.2, 29.0, 28.9, 25.8, 22.5, 14.4; HRMS (ESI-TOF): [M]^2+^ calcd for C_40_H_56_N_2_O_2_ 298.2165, found 298.2175.

*4,4′-(Naphthalene-1,5-diylbis(oxy))bis(1-undecylpyridin-1-ium)dibromide* (**7e**). White solid (0.42 g, 0.54 mmol, 54% yield). C_42_H_60_Br_2_N_2_O_2_, M = 784.76, M.p. = 293–295 °C, IR (neat/cm^−1^): 3437, 2923, 2851, 1642, 1578, 1496, 1398, 1286, 1176, 942, 803; ^1^H NMR (300 MHz, DMSO-*d*_6_) δ 8.97 (d, *J* = 7.2 Hz, 4H, 4CH_Pyr_), 7.95 (d, *J* = 7.6 Hz, 2H, 2CH_Ar_), 7.76 (t, *J* = 7.6 Hz, 2H, 2CH_Ar_), 7.71 (d, *J* = 7.6 Hz, 2H, 2CH_Ar_), 7.67 (d, *J* = 7.2 Hz, 4H, 4CH_Pyr_), 4.50 (t, *J* = 7.3 Hz, 4H, 2N^+^CH_2_), 1.96–1.77 (m, 4H, 2CH_2_), 1.38–1.14 (m, 32H, 16CH_2_), 0.86 (t, *J* = 6.4 Hz, 6H, 2CH_3_); ^13^C NMR (75 MHz, DMSO-*d*_6_) δ 169.8, 148.6, 147.5, 128.5, 127.9, 120.7, 119.7, 115.4, 59.8, 31.7, 31.0, 29.3, 29.2, 29.1, 29.0, 28.9, 25.8, 22.5, 14.4; HRMS (ESI-TOF): [M]^2+^ calcd for C_42_H_60_N_2_O_2_ 312.2322, found 312.2328.

*4,4′-(Naphthalene-1,5-diylbis(oxy))bis(1-dodecylpyridin-1-ium)dibromide* (**7f**). White solid (0.76 g, 0.93 mmol, 93% yield). C_44_H_64_Br_2_N_2_O_2_, M = 812.82, M.p. = 297–300 °C, IR (neat/cm^−1^): 3434, 2921, 2851, 1642, 1581, 1500, 1469, 1402, 1283, 1181, 943, 788; ^1^H NMR (500 MHz, DMSO-*d*_6_) δ 8.99 (d, *J* = 7.3 Hz, 4H, 4CH_Pyr_), 7.95 (d, *J* = 7.9 Hz, 2H, 2CH_Ar_), 7.76 (t, *J* = 7.9 Hz, 2H, 2CH_Ar_), 7.72 (d, *J* = 7.9 Hz, 2H, 2CH_Ar_), 7.68 (d, *J* = 7.3 Hz, 4H, 4CH_Pyr_), 4.52 (t, *J* = 7.3 Hz, 4H, 2N^+^CH_2_), 1.93–1.82 (m, 4H, 2CH_2_), 1.37–1.16 (m, 34H, 18CH_2_), 0.86 (t, *J* = 6.8 Hz, 6H, 2CH_3_); ^13^C NMR (75 MHz, DMSO-*d*_6_) δ 169.8, 148.6, 147.6, 128.5, 127.9, 120.7, 119.8, 115.4, 59.7, 31.8, 31.1, 29.5, 29.4, 29.3, 29.2, 28.9, 25.8, 22.6, 14.4; HRMS (ESI-TOF): [M]^2+^ calcd for C_44_H_64_N_2_O_2_ 326.2478, found 326.2481.

#### 3.1.5. Water Solubility

Bis-QACs **5b**–**g**, **6b**–**g**, and **7a**–**f** (100 mg) were added to 10 mL of distilled water. Bis-QACs **5a** and **6a** (200 mg) were added to 10 mL of distilled water. The mixture was stirred at 25 °C for 24 h. Insoluble residue was filtered through a Schott filter, washed with cold acetone, and then dried at 60 °C for 24 h. Solubility in water of bis-QACs was calculated by the following formula [80]:

Solubility = (m_1_ − m_2_)/10

where m_1_ (mg) is the initial mass of the solids and m_2_ (mg) is the weight of the undissolved and dried solids.

### 3.2. Microbiological Assay

#### 3.2.1. Bacterial and Fungi Strains

Laboratory strains of the microorganisms *E. coli* ATCC 25922, *K. pneumoniae* ATCC 70060, *S. aureus* ATCC 43300, *P. aeruginosa* ATCC 27853, and *A. baumannii* ATCC 15308 were obtained from the State Collection of Pathogenic Microorganisms (Obolensk, Russia). Clinical strains of *E. coli* B-3421/19, *E. coli* C226691/21, *K. pneumoniae* B-2523/18, *K. pneumoniae* C24540/21, *S. aureus* B-8648, *S. aureus* MRSA 0576, *P. aeruginosa* B-2099/18, *P. aeruginosa* C23520/21, *A. baumannii* B-2926/18, *A. baumannii* C23382/21, *C. auris* F-2035, and *C. auris* KA9 were isolated in the Molecular Microbiology department of the Federal Budget Institution of Science State Research Center for Applied Biotechnology and Microbiology (FBSI SRC PMB, Obolensk, Russia) from clinical samples in the investigation of infection cases in 2018, 2019, and 2021.

#### 3.2.2. Identification of Microorganisms

Species identification of microorganisms was carried out on a MALDI-TOF Biotyper mass spectrometer (Bruker, Billerica, MA, USA) according to the standard microflex User Manual. One part of the daily bacterial culture was introduced into a 2 mL tube (Eppendorf, Hamburg, Germany) containing 300 µL of deionized water and triturated until a homogeneous suspension was obtained. Then, 900 µL of 96% ethanol was added and shaken. The tube was then centrifuged at 12,000× *g* for 2 min. The supernatant was discarded, and the precipitate was dried in air. After drying, the precipitate was thoroughly mixed with 30 µL of 70% formic acid, incubated for 10 min at room temperature, and then mixed with 30 µL of acetonitrile (Sigma-Aldrich, St. Louis, MO, USA) and incubated for another 10 min. This mixture was centrifuged at 12,000× *g* for 2 min; then, 1 µL of the supernatant was transferred to an MSP 96 Polished Steel MALDI Target Plate (Bruker Daltonik GmbH, Bremen, Germany), air-dried, and covered with 1 µL of a saturated solution of α-cyano-4-hydroxycinnamic acid (Bruker Daltonik, Bremen, Germany). The MALDI target plate was placed in a MALDI-TOF microflex LRF instrument (Bruker Daltonik, Bremen, Germany). The data acquisition settings were as follows: ion source 1 at 19.50 kV, ion source 2 at 18.22 kV, lens at 7.01 kV, and a mass range of 2000 to 20,000 Da. Spectrum registration was performed automatically using MALDI Biotyper RTC 3.1 software (Bruker Daltonik, Bremen, Germany). The obtained spectra were compared with the reference spectra of the FFL v1.0 database. Identification scores ≥ 2.0 and 1.7–1.99 indicated recognition of the species and genus levels, respectively, while scores < 1.7 indicated a lack of reliable data.

#### 3.2.3. Cultivation of Microorganisms

Microorganisms were cultivated using agar and Mueller–Hinton Broth (MHB). Cultivation of microorganisms was carried out for 20–24 h at a temperature of 37 °C.

#### 3.2.4. Determination of the Biofilms

The efficiency of bacterial biofilm formation was determined using a method based on the ability of the crystal violet dye to bind with cells and biofilm matrix. The growth of biofilms of test strains of microorganisms was carried out in 96-well plates according to the standard method [81]. To obtain biofilms, 96-well, flat-bottomed culture plates were used. In each well, 200 µL of a daily bacterial culture was inoculated at a concentration of 10^6^ CFU/mL and cultivated for 24 h at 37 °C. Then, the medium with planktonic cells was carefully taken from the plate. Wells with biofilms were washed for 2–3 min using sterile PBS buffer (NaCl—8 g, KCl—0.2 g, Na_2_HPO_4_—1.44 g, KH_2_PO_4_—0.24 g per 1 L, pH = 7.4) in the same volume to remove the residual planktonic cells. After washing, PBS was completely removed by pipetting. Then, 200 µL of a filtered 0.1% solution of gentian violet was added to each well, and the biofilms were incubated with the dye for 10–15 min at room temperature. The dyes were completely removed from the well by pipetting. The unbound dye was thoroughly washed off with PBS. The plates were inverted onto filter paper and dried. After complete drying of the surface, a 200 µL mixture of ethanol–isopropanol (1:1) was added to the wells. The dye was washed off from the surface of the wells, taken, and placed in clean flat-bottomed plates. The optical density of the resulting solution was measured at a wavelength of 590 nm using an xMark™ Microplate Absorbance Spectrophotometer (Bio-Rad Laboratories Inc., Hercules, CA, USA). The measurement results were interpreted by comparing the OD_590_ values of the samples with control values (pure solvent, without any dye being added).

The absence of biofilm was recorded at OD_590_ sample ≤ OD_590_ control; weak degree of biofilm production at OD_590_ (control) < OD_590_ (sample) ≤ 2 × OD_590_ (control); average degree of biofilm production at 2 × OD_590_ (control) < OD_590_ (sample) ≤ 4 × OD_590_ (control); high degree of biofilm production at OD_590_ control < 4 × OD_590_ sample [82]. All experiments were carried out in triplicate.

#### 3.2.5. Antimicrobial and Antibiofilm Activity

The synthesized naphthalene bis-QACs and reference compounds were dissolved in the DMSO/water system at a ratio of 1/9 with a final concentration of 1000 mg/L. For better dissolution, the samples were sonicated for 15 to 60 min at 40 °C. The MIC and MBC of the antimicrobials was determined by the microdilution method in culture broth, as indicated by the Clinical and Laboratory Standards Institute of the United States of America [83]. For this, 2-fold dilutions of the tested solutions (500–0.25 mg/L) were prepared in nutrient broth. The nutrient broth with the tested QACs (0.1 mL) was added to 12 wells in the horizontal rows of the culture plate. In separate rows, nutrient broth without QACs was added for control measures. In the case of antibiofilm analysis, nutrient broth with the appropriate concentration of the tested compounds was added in 0.1 mL to 12 wells in horizontal rows of a culture plate with washed biofilms, which were prepared according to the abovementioned method. From single colonies grown on Mueller–Hinton agar (MHA) medium at 37 °C for 18 h, a suspension was prepared with an optical density of 0.5 according to the McFarland standard in sterile saline, which corresponds to approximately 1–2 × 10^8^ CFU/mL. The suspension was then diluted 100-fold by adding 0.2 mL of the suspension to a flask containing 19.8 mL of MHA. The concentration of microorganisms, in this case, was 10^6^ CFU/mL. An amount of 0.1 mL of the initial suspension was added to the wells with the test drug and control wells with broth. The final concentration of the microorganisms in each well was 5 × 10^5^ CFU/mL. The plates were covered with lids and placed in a thermostat (37 °C) for 20 h. The presence of bacterial growth was taken into account visually (according to the presence of turbidity in the well). The MIC or MBIC were taken to be the minimum concentration of the preparation at which bacterial growth was absent after 48 h of incubation. MBC, MFC, or MBEC were determined by the results of inoculation on dense nutrient media. To do this, from all the wells in which there was no visible growth (according to the presence of turbidity), 10 µL was sown on MHA. The results were taken into account by the presence of culture growth at the site of application after 48 h of incubation at 37 °C. If there was no growth in the well, but the growth of the studied culture was observed when seeding from this well on solid nutrient medium, then this concentration was taken as the MIC or MBIC. The lowest concentration was taken as MBC, MFC, or MBEC, at which cell growth was completely suppressed when seeded on a dense nutrient medium. Since the limit of detection for this technique is 10 CFU/mL, the absence of any growth on the MHA plate indicated that the concentration lay below this value. The initial concentration of 10^5^ CFU/mL had thus been reduced to below 10 CFU/mL. Consequently, the MBC, MFC, or MBEC were effectively deemed to be the minimum concentration of antimicrobial capable of inactivating more than 99.99% of the bacteria or fungi present. Three replicates were performed for each strain and antimicrobial compound.

#### 3.2.6. Lipophilicity–Activity Relationship

The LogP values for reference compounds (series **5**) and the synthesized naphthalene bis-QACs (series **6** and **7**) were calculated using the online platform Chemicalize, in June of 2024, https://chemicalize.com/, developed by ChemAxon. Log 1/MBC and 1/MBIC values of QACs were used as an indicator of activity

#### 3.2.7. Bacterial Resistance

The selection of resistant microorganisms was carried out according to the previously proposed method of experimental adaptive evolution by regular culture of the bacterial strains in a nutrient broth containing serial dilutions of the antibacterial drug [84,85,86]. To do this, in 2 mL of MHB, a series of two-fold dilutions of the drug were prepared, starting with a concentration that was half the MIC. During the initial inoculation, 20 µL of 10^7^ CFU/mL of daily culture was added to each tube and incubated at 37 °C. After two to three days of incubation, 50 µL from the last turbid tube with a higher concentration was divided into new tubes with increasing concentrations of the tested antibacterial drug. During subsequent transfers to increasing concentrations, 50 µL was also taken from the last tube, in ascending order, in which visual growth was observed. The selection process was stopped if the MBC had not changed within 6–8 passages.

#### 3.2.8. Scanning Electron Microscopy Imaging

Obtaining bacterial biofilms for SEM imaging was carried out using a static method that simulates a biofilm on the surface of medical instruments. An aluminum plate with a hole pressed out in the center was transferred into a sterile Petri dish, at the bottom of which a glass slide was previously placed. Then, 200 μL of a bacterial suspension prepared in MHB at a concentration of 10^6^ CFU/mL was poured into the hole. Additionally, 2 mL of sterile distilled water was poured into the bottom of the cup to prevent the sample from drying out. Another 200 μL of MHB was poured into the hole of the imprinted plate and incubated under static conditions for 72 h at 37 °C. At the end of incubation, excess broth was removed from the surface of the plate and dried in a laminar air flow.

To prepare the obtained samples for SEM imaging, the biofilms were fixed on an aluminum plate and then the plate was submerged in 20% glutaraldehyde for 1 h. After the designated time, the plate with the biofilm fixed on it was washed three times with deionized water and dried in air. Glutaraldehyde is considered the optimal procedure for preventing structural damage to sessile growing bacteria due to its ability to quickly and irreversibly cross-link proteins, anchoring the biofilm to the surface. Before recording SEM images, samples were fixed on an aluminum table with double-sided conductive carbon tape. Using a Cressingtor 208HR magnetron sputtering device (Cressington Scientific Instruments Ltd, Watford, UK), 10 nm of gold/palladium alloy (60/40) was sputtered onto the surface of the samples. Images were obtained using a secondary electron detector at an accelerating voltage of 3–5 kV. To select the best method for visualizing the biofilm, samples were taken both with and without a coating of a conductive carbon layer. The microstructure of the samples was studied by field emission scanning electron microscopy (FE-SEM) using a Hitachi SU8000 electron microscope (Hitachi High-Tech, Tokyo, Japan). The morphology of the samples was studied taking into account the correction for the surface effects of deposition of a conductive layer.

#### 3.2.9. Time–Kill Kinetics

Determination of the time–kill kinetics was performed on *E. coli* ATCC 25922 and *S. aureus* ATCC 43300 strains in the presence of hit compounds.

The antibacterial activity of the compounds was assessed in an experiment to study the survival of bacterial cells in MHB containing the test compounds at a concentration equal to the MBC at different exposure times.

Mueller–Hinton broth was prepared with appropriate additions of hit compounds.

During this study, different time intervals for exposure to compounds were selected, corresponding to 5 min, 10 min, 15 min, 30 min, 45 min, 60 min, 75 min, 90 min, 105 min, and 120 min for each strain under study. The test microorganism in logarithmic growth phase was added to each tube containing 4 mL of Mueller–Hinton broth and the test compound at the required concentration. The final concentration of microbial cells in the vial was 5 × 10^5^ CFU/mL. The tubes were incubated at 37 °C. At certain cultivation time intervals, aliquots of the suspension (100 μL) were taken, five successive 10-fold dilutions of the selected suspension were made, and 0.1 mL was inoculated onto a solid nutrient medium to determine the concentration of microbial cells in the test tube. All studies were carried out in triplicate, and test tubes without the addition of the compounds under study were used as a negative control. Incubation of bacteria on dishes with a dense nutrient medium was carried out at a temperature of 37 °C for 24 h.

#### 3.2.10. MTT Assay

The synthesized naphthalene bis-QACs and reference compounds were dissolved in the PBS with a final concentration of 250–1000 mg/L. For better dissolution, the samples were sonicated for 15 to 60 min at 40 °C.

The HEK 293T were cultured in the DMEM containing 10% FBS and antibiotic-antimycotic (10,000 units/mL of penicillin, 10,000 μg/mL of streptomycin, and 25 μg/mL of Amphotericin B) in a 5% CO_2_ humidity incubator at 37 °C. Cellular toxicity assays were carried out by the MTT (3-(4,5-dimethyl-2-thiazolyl)-2,5-diphenyl-2*H*-tetrazolium bromide) method on HEK 293T cells. Cells were plated into 96-well plates at a density of 5000 cells per well in 100 mL of medium and grown for overnight. The cells were then exposed to the tested compounds at different concentrations (1−1000 mg/L) for 48 h. A 10 µL MTT solution (5.0 mg/mL) was added to each well. After further incubation for another 4 h, formazan formed from MTT was extracted in 100 μL of DMSO for 15 min of standby. The absorbance at 570 nm was then determined on a microplate reader.

The determined cytotoxicity is expressed as percentage cell viability using the following equation:

%Cell viability = ((OD_sample_ − OD_blank_)/(OD_control_ − OD_blank_)) × 100

In this equation, OD_sample_, OD_blank_, and OD_control_ are the measured absorption of the test, blank, and control sample. The experimental results are expressed as mean ± standard deviation (SD).

The statistical analysis and the representative graphs thereof were carried out using GraphPad Prism 8.0 (GraphPad Software, La Jolla, CA, USA). The cytotoxic concentration (CC50) values were obtained by non-linear regression analysis. The inhibitory curves were built using the log(inhibitor) vs. normalized response—variable slope equation with least squares fit.

#### 3.2.11. Hemolysis

Hemolytic activity was tested through using the human erythrocytes (RBCs) isolated from the fresh whole blood from the healthy donor with centrifugation (three times with 3 volumes of phosphate-buffered saline (PBS)). Stock solutions of the tested compounds were prepared in PBS at a concentration of 128 mg/L and serially diluted by the 2-fold dilution method, and then 100 μL of each dilution was added to 96-well plates. RBCs were resuspended in PBS to 5% (*v*/*v*) and cell suspension (100 μL) was added to 96-well plates, and then the plates were incubated at 37 °C for 1 h. The negative control was an RBC suspension with PBS only, and the positive control was PBS containing 0.1% (*v*/*v*) Triton X-100. After incubation, the mixture was centrifuged (1000 g for 10 min), and then the supernatant (100 μL) was pipetted into a fresh 96-well plate. Finally, hemolysis was determined by measuring the supernatant absorbance at 405 nm (OD405) using a xMark microplate absorbance spectrophotometer (Bio-Rad, USA).

The hemolysis activity was calculated by the following formula:

%Hemolysis = ((OD_sample_ − OD_blank_)/(OD_control_ − OD_blank_)) × 100%

In this equation, OD_sample_, OD_blank_, and OD_control_ are the measured absorption of the test, blank, and control sample. The experimental results are expressed as mean ± standard deviation (SD).

The statistical analysis and the representative graphs thereof were carried out using GraphPad Prism 8.0 (GraphPad Software, La Jolla, CA, USA). The hemolytic concentration (HC50) values were obtained by non-linear regression analysis. The inhibitory curves were built using the log(inhibitor) vs. normalized response—variable slope equation with least squares fit.

## 4. Conclusions

In summary, through a simple and unified synthetic approach, we have expanded the panel of antimicrobial agents of bis-QACs naphthalene derivatives. Microbiological analysis showed that hit compounds have a broad spectrum of antimicrobial and antibiofilm activity, and the structure symmetry and lipophilicity influenced antibacterial effectiveness. Further investigation of bis-QACs **5d** and **6d** indicated the retention of activity during the transition to the next generation of resistant microorganisms and membrane-destructive action against *S. aureus* and *P. aeruiginosa* biofilms according to SEM imaging assay. Moreover, compound **5d** demonstrated good selectivity compared to commercial QACs and a low rate of resistance formation after more than a month of exposure to *S. aureus* and *K. pneumoniae*. However, some of the observed structure–activity correlations were not typical for bis-QACs. For example, the increased activity of **5e**, **5f,** and **6e** against biofilms compared to planktonic cells. Thus, we are encouraged to further research this class of compounds in order to transition the stagnating market of antiseptics and disinfectants into a new, more effective generation of biocidal compositions.

## Figures and Tables

**Table 1:** Antibacterial activity of bis-QACs **5**–**7** compared to commercial QACs.

Compounds	Reference Strains					Clinical Isolates				
	*Sa*	*Ec*	*Kp*	*Ab*	*Pa*	*Sa*	*Ec*	*Kp*	*Ab*	*Pa*
*Planktonic Cells (MIC/MBC [mg/L])*										
**5a**: 2,7-, n = 7, Br	1	8	250	>500	250	2	32	63	250	16
	8	32	500	>500	500	4	63	500	250	16
**5b**: 2,7-, n = 8, Br	0.5	4	8	63	32	0.5	4	16	125	63
	4	4	16	63	125	4	4	32	250	125
**5c**: 2,7-, n = 8, I	0.5	4	16	125	63	1	4	16	32	63
	4	4	16	125	250	4	4	32	63	63
**5d**: 2,7-, n = 9, Br	0.5	2	16	32	32	0.5	8	8	32	63
	4	2	32	63	63	4	8	16	63	63
**5e**: 2,7-, n = 10, Br	4	8	32	16	63	4	32	32	32	63
	16	8	32	32	250	16	125	63	32	125
**5f**: 2,7-, n = 11, Br	16	32	63	16	250	16	63	63	32	125
	32	32	250	16	500	63	>500	125	32	250
**5g**: 2,7-, n = 12, Br	63	32	250	16	500	32	125	500	125	>500
	125	63	500	32	>500	500	>500	500	>500	>500
**6a**: 1,6-, n = 7, Br	2	32	125	500	63	4	32	32	500	500
	16	63	500	500	63	4	32	500	500	500
**6b**: 1,6-, n = 8, Br	0.5	4	8	250	32	0.5	4	8	250	125
	2	4	63	250	250	4	16	32	500	250
**6c**: 1,6-, n = 8, I	0.5	4	125	125	125	0.5	8	32	63	125
	4	8	125	125	250	2	16	125	125	250
**6d**: 1,6-, n = 9, Br	2	8	16	63	32	2	8	32	125	63
	8	8	32	63	63	4	16	63	125	63
**6e**: 1,6-, n = 10, Br	4	8	32	8	125	4	8	32	32	63
	16	8	63	16	250	16	125	32	32	125
**6f**: 1,6-, n = 11, Br	8	32	63	16	16	8	63	63	63	63
	32	63	250	32	32	16	63	250	63	125
**6g**: 1,6-, n = 12, Br	32	125	125	16	32	16	63	125	63	250
	125	125	250	32	32	63	125	500	63	250
**7a**: 1,5-, n = 7, Br	0.5	63	63	250	500	4	63	125	250	500
	8	63	125	500	500	16	125	250	500	500
**7b**: 1,5-, n = 8, Br	1	4	32	125	63	1	8	32	63	125
	4	8	63	250	125	4	8	63	125	125
**7c**: 1,5-, n = 9, Br	1	4	16	63	63	2	8	63	63	63
	4	4	16	250	250	16	8	125	63	63
**7d**: 1,5-, n = 10, Br	2	8	16	32	8	4	8	63	32	8
	4	8	32	32	16	8	16	63	32	16
**7f**: 1,5-, n = 12, Br	4	63	>500	500	>500	16	125	>500	500	>500
	16	63	>500	500	>500	32	250	>500	500	>500
**MIR**	2	8	32	63	63	4	8	16	63	125
	8	8	32	63	125	16	8	32	250	125
**CPC**	4	8	63	32	500	2	8	8	16	32
	16	8	250	125	>500	16	63	16	32	125
**OCT**	0.5	0.5	4	32	8	0.5	2	2	32	32
	2	0.5	8	125	16	2	4	4	125	63

Note: *Ec*—*E. coli* ATCC 25922 (laboratory strain), *E. coli* B-3421/19 (clinical isolate); *Kp*—*K. pneumoniae* ATCC 70060 (laboratory strain), *K. pneumoniae* B-2523/18 (clinical isolate); *Sa*—*S. aureus* ATCC 43300 (laboratory strain), *S. aureus* B-8648 (clinical isolate); *Pa*—*P. aeruginosa* ATCC 27853 (laboratory strain), *P. aeruginosa* B-2099/18 (clinical isolate); *Ab*—*A. baumannii* ATCC 15308 (laboratory strain), *A. baumannii* B-2926/18 (clinical isolate); MIC—minimum inhibitory concentration; MBC—minimum bactericidal concentration; color indicates the best result in series, color indicates one dilution difference with the best result, color indicates two dilution difference with the best result.

**Table 2:** Antibiofilm activity of bis-QACs **5**–**7** compared to commercial QACs.

Compounds	Reference Strains					Clinical Isolates				
	*Sa*	*Ec*	*Kp*	*Ab*	*Pa*	*Sa*	*Ec*	*Kp*	*Ab*	*Pa*
*Biofilms (MBIC/MBEC [mg/L])*										
**5a**: 2,7-, n = 7, Br	>500	>500	>500	>500	>500	>500	>500	>500	>500	>500
	>500	>500	>500	>500	>500	>500	>500	>500	>500	>500
**5b**: 2,7-, n = 8, Br	250	8	63	250	250	250	16	63	250	125
	250	16	250	500	>500	250	32	500	500	>500
**5c**: 2,7-, n = 8, I	63	4	63	125	250	63	8	63	250	250
	63	4	125	250	>500	125	8	250	250	>500
**5d**: 2,7-, n = 9, Br	4	8	32	125	250	4	16	63	250	250
	32	32	250	500	>500	16	63	250	>500	500
**5e**: 2,7-, n = 10, Br	2	16	32	250	250	4	63	63	>500	125
	4	32	500	500	500	4	63	500	>500	500
**5f**: 2,7-, n = 11, Br	2	32	63	125	>500	2	250	125	>500	>500
	4	63	500	500	>500	4	>500	>500	>500	>500
**5g**: 2,7-, n = 12, Br	63	250	>500	500	>500	32	32	>500	500	>500
	250	>500	>500	>500	>500	125	63	>500	>500	>500
**6a**: 1,6-, n = 7, Br	4	63	>500	>500	>500	4	32	>500	>500	>500
	63	63	>500	>500	>500	8	32	>500	>500	>500
**6b**: 1,6-, n = 8, Br	8	32	63	>500	>500	4	63	125	>500	>500
	63	32	>500	>500	>500	32	63	500	>500	>500
**6c**: 1,6-, n = 8, I	4	8	63	250	250	8	8	63	250	250
	63	16	250	500	>500	63	32	250	500	>500
**6d**: 1,6-, n = 9, Br	8	8	63	63	>500	4	8	63	63	>500
	63	8	500	250	>500	8	16	125	125	>500
**6e**: 1,6-, n = 10, Br	8	32	32	32	>500	8	8	63	63	500
	32	125	250	250	>500	32	16	500	125	>500
**6f**: 1,6-, n = 11, Br	16	63	125	63	250	8	125	125	125	>500
	63	250	>500	500	>500	16	250	>500	500	>500
**6g**: 1,6-, n = 12, Br	32	>500	>500	>500	500	32	>500	>500	>500	500
	250	>500	>500	>500	>500	125	>500	>500	>500	>500
**7a**: 1,5-, n = 7, Br	63	250	125	500	500	125	250	125	500	500
	63	500	500	500	>500	125	250	500	>500	>500
**7b**: 1,5-, n = 8, Br	125	32	32	250	250	125	32	32	250	250
	125	63	250	500	500	250	32	500	>500	500
**7c**: 1,5-, n = 9, Br	63	16	32	125	125	32	16	32	250	250
	63	63	250	500	500	63	125	500	500	500
**7d**: 1,5-, n = 10, Br	16	32	32	>500	250	16	16	63	>500	125
	63	32	250	>500	500	32	16	250	>500	500
**7f** 1,5-, n = 12, Br	>500	>500	>500	>500	>500	>500	>500	>500	>500	>500
	>500	>500	>500	>500	>500	>500	>500	>500	>500	>500
**MIR**	16	32	125	63	500	16	63	63	63	500
	32	32	500	250	>500	32	250	500	250	>500
**CPC**	16	16	63	250	>500	8	125	63	250	>500
	63	63	250	500	>500	32	250	500	500	>500
**OCT**	4	8	16	250	125	4	16	16	32	250
	8	16	63	250	500	8	125	125	250	250

Note: *Ec*—*E. coli* ATCC 25922 (laboratory strain), *E. coli* B-3421/19 (clinical isolate); *Kp*—*K. pneumoniae* ATCC 70060 (laboratory strain), *K. pneumoniae* B-2523/18 (clinical isolate); *Sa*—*S. aureus* ATCC 43300 (laboratory strain), *S. aureus* B-8648 (clinical isolate); *Pa*—*P. aeruginosa* ATCC 27853 (laboratory strain), *P. aeruginosa* B-2099/18 (clinical isolate); *Ab*—*A. baumannii* ATCC 15308 (laboratory strain), *A. baumannii* B-2926/18 (clinical isolate); MBIC—minimum biofilm inhibitory concentration, MBEC—minimum biofilm eradication concentration; color indicates the best result in series, color indicates one dilution difference with the best result, color indicates two dilution difference with the best result.

**Table 3:** The antimicrobial activity of compounds **5d** and **6d** against five additional multi-resistant Gram-positive and Gram-negative bacterial strains, and two fungi strains from clinical samples in the investigation of infection cases during the COVID-19 period in 2021.

Strains	Compounds				
	5d	6d	OCT	CPC	MIR
*Planktonic bacterial cells (MIC/MBC [mg/L])*					
*S. aureus* MRSA 0576	1	1	1	1	4
	8	2	2	1	8
*E. coli* C226691/21	4	4	4	32	32
	8	4	4	>500	250
*K. pneumoniae* C24540/21	32	32	4	32	32
	32	63	32	500	250
*A. baumannii* C23382/21	63	125	4	16	16
	63	250	63	250	16
*P. aeruginosa* C23520/21	32	32	4	250	250
	32	63	125	>500	500
*Planktonic fungi cells (MIC/MFC [mg/L])*					
*C. auris* KA9	2	4	2	4	16
	63	16	4	8	63
*C. auris* F-2035	2	4	1	4	8
	8	8	1	8	32
*Biofilms (MBIC/MBEC [mg/L])*					
*S. aureus* MRSA 0576	8	8	4	4	4
	32	16	63	32	63
*E. coli* C226691/21	16	8	8	63	125
	16	32	8	125	125
*K. pneumoniae* C24540/21	32	32	63	250	250
	250	250	250	250	250
*A. baumannii* C23382/21	250	250	16	32	32
	>500	>500	16	32	63
*P. aeruginosa* C23520/21	32	63	125	250	250
	63	63	250	>500	>500
*C. auris* KA9	8	4	2	16	32
	63	125	16	32	63
*C. auris* F-2035	1	4	0.5	8	32
	4	16	32	32	63

Note: MIC—minimum inhibitory concentration; MBC—minimum bactericidal concentration; MFC—minimum fungicidal concentration; MBIC—minimum biofilm inhibitory concentration, MBEC—minimum biofilm eradication concentration; color indicates the best result in series, color indicates one dilution difference with the best result, color indicates two dilution difference with the best result.

**Table 4:** Solubility in water at 25 °C of naphthalene-derived bis-QACs **5**–**7** compared to OCT.

Compounds	Solubility in Water (mg/mL)	Compounds	Solubility in Water (mg/mL)	Compounds	Solubility in Water (mg/mL)
**5a**: 2,7-, n = 7, Br	17.4	**6a**: 1,6-, n = 7, Br	16.7	**7a**: 1,5-, n = 7, Br	1.6
**5b**: 2,7-, n = 8, Br	7.2	**6b**: 1,6-, n = 8, Br	7.5	**7b**: 1,5-, n = 8, Br	1.1
**5c**: 2,7-, n = 8, I	1.1	**6c**: 1,6-, n = 8, I	0.9	**7c**: 1,5-, n = 9, Br	0.4
**5d**: 2,7-, n = 9, Br	4.0	**6d**: 1,6-, n = 9, Br	4.4	**7d**: 1,5-, n = 10, Br	0.1
**5e**: 2,7-, n = 10, Br	1.7	**6e**: 1,6-, n = 10, Br	1.9	**7e**: 1,5-, n = 11, Br	<<0.1
**5f**: 2,7-, n = 11, Br	0.4	**6f**: 1,6-, n = 11, Br	0.6	**7f**: 1,5-, n = 12, Br	<<0.1
**5g**: 2,7-, n = 12, Br	0.1	**6g**: 1,6-, n = 12, Br	0.1	OCT	2.2

**Table 5:** MTT assay and selectivity indices of **5a**–**g** and **6a**–**g** compared to commercial QACs.

Compounds	CC_50_, mg/L (HEK-293T)	SI_MIC_/SI_MBC_ (Reference Strains)				
		*Sa*	*Ec*	*Kp*	*Ab*	*Pa*
**5a**: 2,7-, n = 7, Br	1.58	1.580	0.198	0.006	0.003	0.006
		0.198	0.049	0.003	0.003	0.003
**5b**: 2,7-, n = 8, Br	1.00	2.000	0.250	0.125	0.016	0.031
		0.250	0.250	0.063	0.016	0.008
**5d**: 2,7-, n = 9, Br	1.225	2.450	0.613	0.077	0.038	0.038
		0.306	0.613	0.038	0.019	0.019
**5e**: 2,7-, n = 10, Br	4.23	1.058	0.529	0.132	0.264	0.067
		0.264	0.529	0.132	0.132	0.017
**5f**: 2,7-, n = 11, Br	31.59	1.974	0.987	0.501	1.974	0.126
		0.987	0.987	0.126	1.974	0.063
**6a**: 1,6-, n = 7, Br	2.67	1.335	0.083	0.021	0.005	0.042
		0.167	0.042	0.005	0.005	0.042
**6b**: 1,6-, n = 8, Br	1.76	3.520	0.440	0.220	0.007	0.055
		0.880	0.440	0.028	0.007	0.007
**6c**: 1,6-, n = 8, I	1.85	3.700	0.463	0.015	0.015	0.015
		0.463	0.231	0.015	0.015	0.007
**6d**: 1,6-, n = 9, Br	1.255	0.628	0.157	0.078	0.020	0.039
		0.157	0.157	0.039	0.020	0.020
**6f**: 1,6-, n = 11, Br	15.98	1.998	0.499	0.254	0.999	0.999
		0.499	0.254	0.064	0.499	0.499
**6g**: 1,6-, n = 12, Br	52.49	1.640	0.420	0.420	3.281	1.640
		0.420	0.420	0.210	1.640	1.640
**BAC**	0.563	0.005	0.141	0.001	0.001	0.001
		0.002	0.070	0.001	0.001	0.001
**MIR**	1.296	0.648	0.162	0.041	0.021	0.021
		0.162	0.162	0.041	0.021	0.010
**CPC**	2.414	0.604	0.302	0.038	0.075	0.005
		0.151	0.302	0.010	0.019	0.005
**OCT**	0.372	0.744	0.744	0.093	0.012	0.047
		0.186	0.744	0.047	0.003	0.023

Note: CC_50_—half-maximal cytotoxicity concentration; HEK-293T—human embryonic kidney cells 293T; *Ec*—*E. coli* ATCC 25922; *Kp*—*K. pneumoniae* ATCC 70060; *Sa*—*S. aureus* ATCC 43300; *Pa*—*P. aeruginosa* ATCC 27853; *Ab*—*A. baumannii* ATCC 15308; SI—selectivity index; MIC—minimum inhibitory concentration; MBC—minimum bactericidal concentration; color indicates the best SIs in series, color indicates SI close to 1.

**Table 6:** Hemolytic activity and selectivity indices of **5d** and **6d** against reference planktonic strains compared to commercial QACs.

Compounds	HC_50_, mg/L (hRBCs)	SI_MIC_/SI_MBC_ (Reference Strains)				
		*Sa*	*Ec*	*Kp*	*Ab*	*Pa*
**5d**: 2,7-, n = 9, Br	36.12	72.240	18.060	2.258	1.129	1.129
		9.030	18.030	1.129	0.573	0.573
**6d**: 1,6-, n = 9, Br	36.45	18.225	4.556	2.278	0.579	1.139
		4.556	4.556	1.139	0.579	0.579
**BAC**	37.33	0.299	9.333	0.075	0.075	0.075
		0.149	4.666	0.075	0.075	0.075
**MIR**	21.78	10.890	2.723	0.681	0.346	0.346
		2.723	2.723	0.681	0.346	0.174
**OCT**	17.97	35.940	35.940	4.493	0.562	2.246
		8.985	35.940	2.246	0.144	1.123

Note: HC_50_—half-maximal hemolytic concentration; hRBCs—human red blood cells; *Ec*—*E. coli* ATCC 25922; *Kp*—*K. pneumoniae* ATCC 70060; *Sa*—*S. aureus* ATCC 43300; *Pa*—*P. aeruginosa* ATCC 27853; *Ab*—*A. baumannii* ATCC 15308; SI—selectivity index; MIC—minimum inhibitory concentration; MBC—minimum bactericidal concentration; color indicates the best SIs in series higher than 10, color indicates SIs in the 5 to 10 range, color indicates SIs in the 1 to 5 range.

## Data Availability

Data are contained within the article and Supplementary Materials.

## Supplementary Materials

The following supporting information can be downloaded at: https://www.mdpi.com/article/10.3390/molecules29235526/s1, NMR spectra of bis-pyridine scaffolds (**3b**–**c**); ^1^H, ^13^C NMR, and IR spectra of bis-QACs **6a**–**g**, **7a**–**f**; HRMS spectra of bis-QACs; Figure S1: Lipophilicity–activity relationship of QACs. (A) The relationship between the calculated logP and the bactericidal activity (LogMIC^−1^) of bis-QACs **5**–**7** against planktonic Gram-positive *S. aureus*, *E. coli,* and *K. pneumoniae*; (B) the relationship between the calculated LogP and the biofilm inhibition activity (LogMBEC^−1^) of bis-QACs **5**–**7** against Gram-positive *S. aureus*, *E. coli* biofilms; (C) the relationship between the calculated LogP and the bactericidal activity (LogMBC^−1^) of bis-QACs **6a**–**g, 7a**–**f** against all reference strains; Figure S2: SEM images of *P. aeruginosa* ATCC 27853 (A) untreated with QAC, after 5 min and 120 min of treatment with **5d** and *S. aureus* ATCC 43300; (B) untreated with QAC, after 60 min and 120 min of treatment with **5d**. Table S1: Antibacterial activity of bis-QACs **6**–**7** compared to commercial QACs and bis-QACs **5a**–**g**; Table S2: Toxicological assay and selectivity indices of **5a**–**g** and **6a**–**g** against clinical isolates, compared to commercial QACs; Table S3: Toxicological assay and selectivity indices of **5a**–**g** and **6a**–**g** against reference biofilms, compared to commercial QACs; Table S4: Toxicological assay and selectivity indices of **5a**–**g** and **6a**–**g** against clinical biofilms, compared to commercial QACs; Table S5: Hemolytic activity and selectivity indices of **5d** and **6d** against clinical isolates, compared to commercial QACs; Table S6: Hemolytic activity and selectivity indices of **5d** and **6d** against reference biofilms, compared to commercial QACs; Table S7: Hemolytic activity and selectivity indices of **5d** and **6d** against clinical biofilms, compared to commercial QACs.
