# Supplementary material for: Development of Naphthalene-Derivative Bis-QACs as Potent Antimicrobials: Unraveling Structure–Activity Relationship and Microbiological Properties
Source: Molecules. 2024 Nov 22;29(23):5526. doi: 10.3390/molecules29235526 (PMC11644004; doi:10.3390/molecules29235526)
Supplement: Supplementary file 1 [file molecules-29-05526-s001.zip › molecules-3266089-supplementary.pdf]

Supporting Materials

Development of Naphthalene-Derivative bis-QACs as Potent Antimicrobials: Unraveling Structure-Activity Relationship and Microbiological Properties

Nikita A. Frolov [1], Mary A. Seferyan [1], Elena V. Detusheva [2], Elizabeth Son [2], Ilya G. Kolmakov [1,3], Alena S. Kartseva [2], Victoria V. Firstova [2], Anatoly N. Vereshchagin [1,*], Michail N. Elinson [1,*]

[1] *N. D. Zelinsky Institute of Organic Chemistry, Russian Academy of Sciences, Leninsky prospect 47, Moscow 119991, Russia; vereshchagin@ioc.ac.ru (A. N. V.); nikitafrolov298@gmail.com (N. A. F.);*
[2] *State Research Center for Applied Microbiology and Biotechnology, Obolensk, 142279 Serpukhov, Moscow Region, Russia;*
[3] *Lomonosov Moscow State University, 119991, Moscow, Leninskie Gory, 1-3.*

Table of Contents

NMR spectra of bis-pyridine scaffolds (3b-c) S2
^{1}H, ^{13}C NMR and IR spectra of bis-QACs (6a-g) S5
^{1}H and ^{13}C NMR spectra of bis-QACs (7a-f) S15
HRMS spectra of bis-QACs. S24
Figures and Tables S38

NMR spectra of bis-pyridine scaffolds (3b-c)

^{1}H NMR spectrum of 4,4'-(naphthalene-1,6-diylbis(oxy))dipyridine (**3b**)

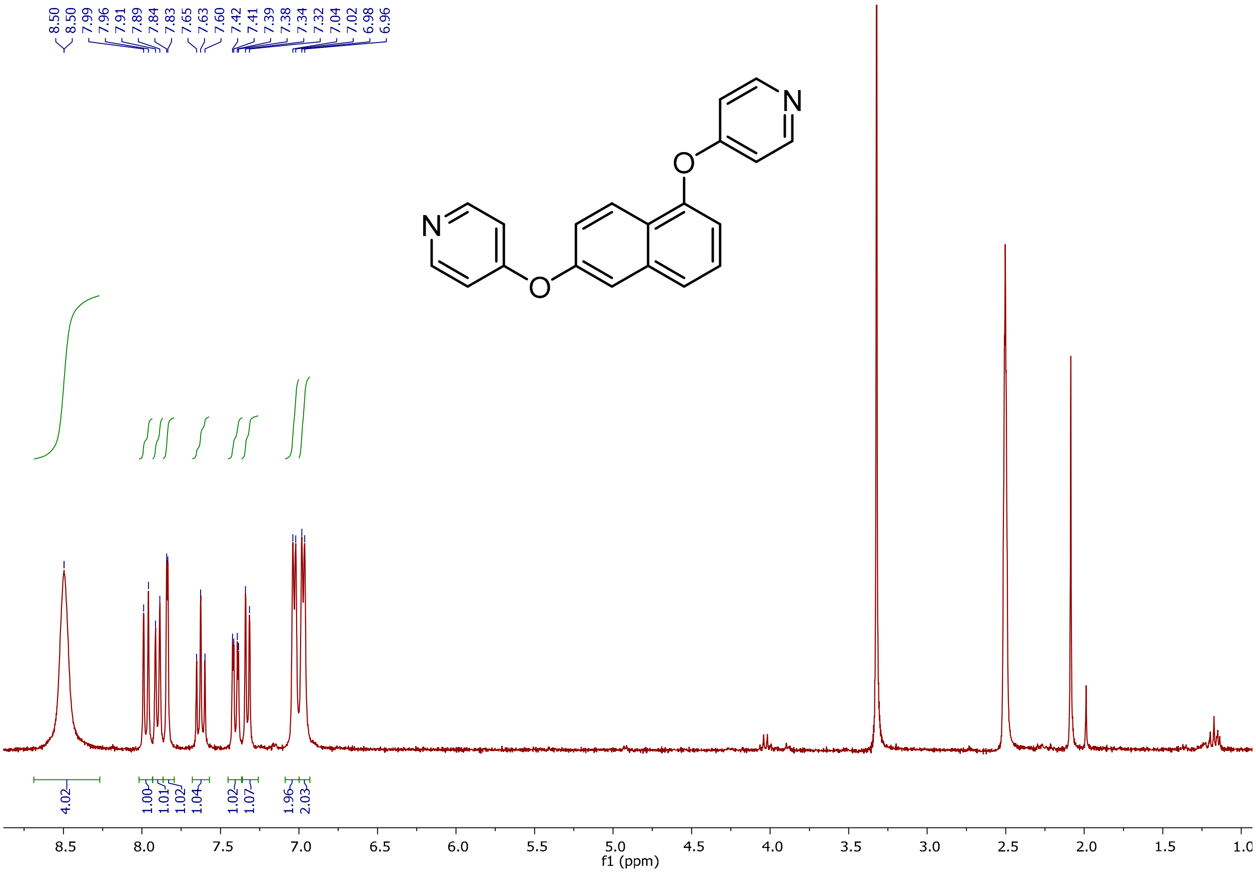

^{13}C NMR spectrum of **3b**

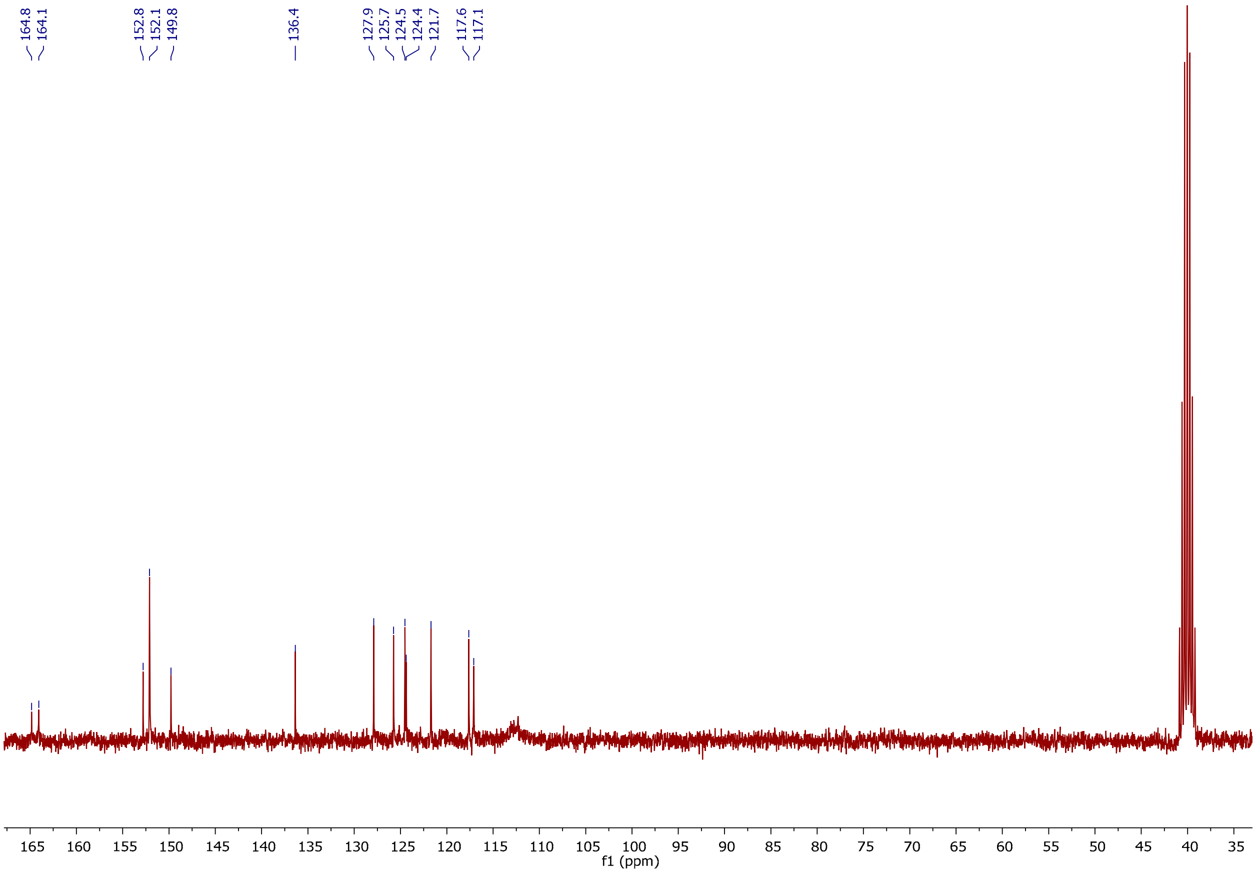

^{1}H NMR spectrum of 1,5-bis(pyridin-4-yloxy)naphthalene (**3c**)

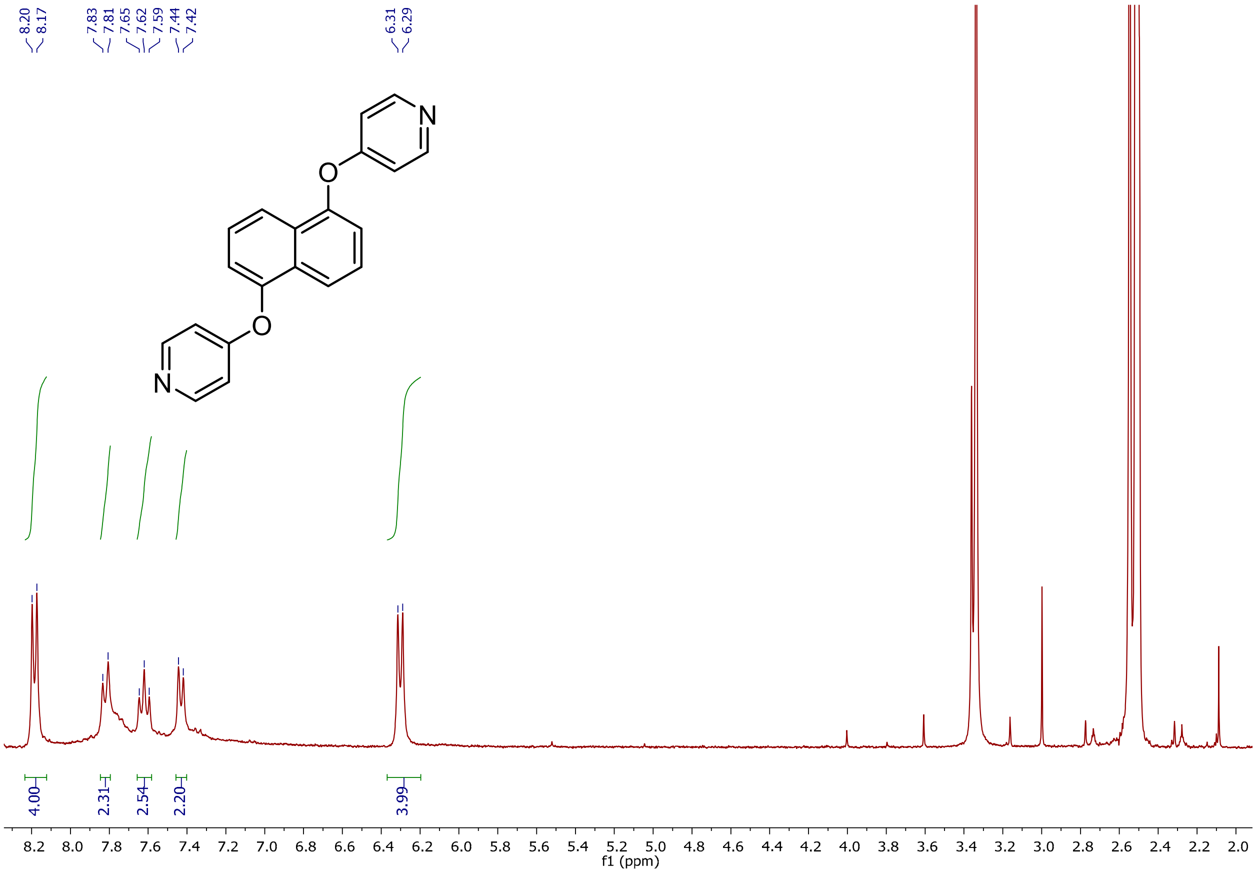

^{13}C **NMR** spectrum of **3c**

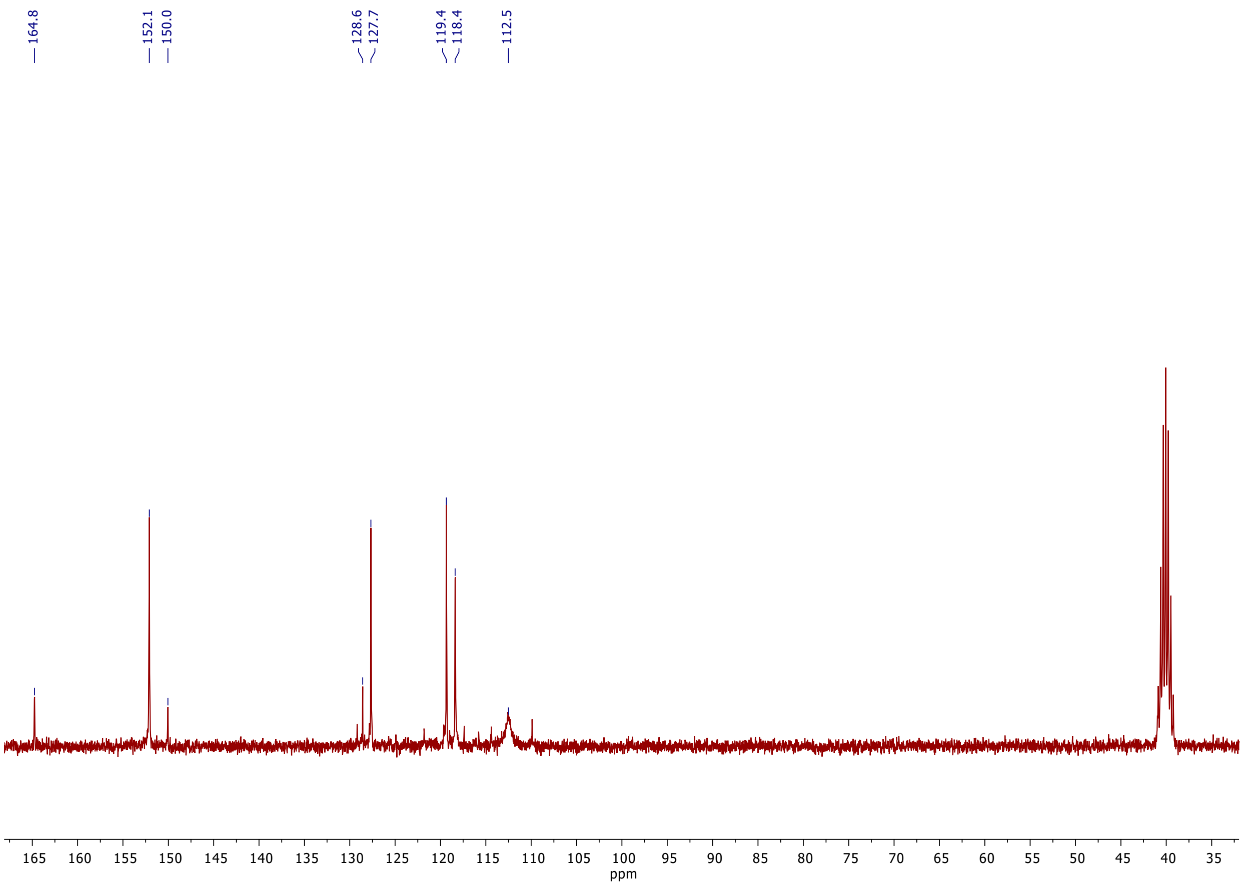

IR spectrum of **3c** (KBr)

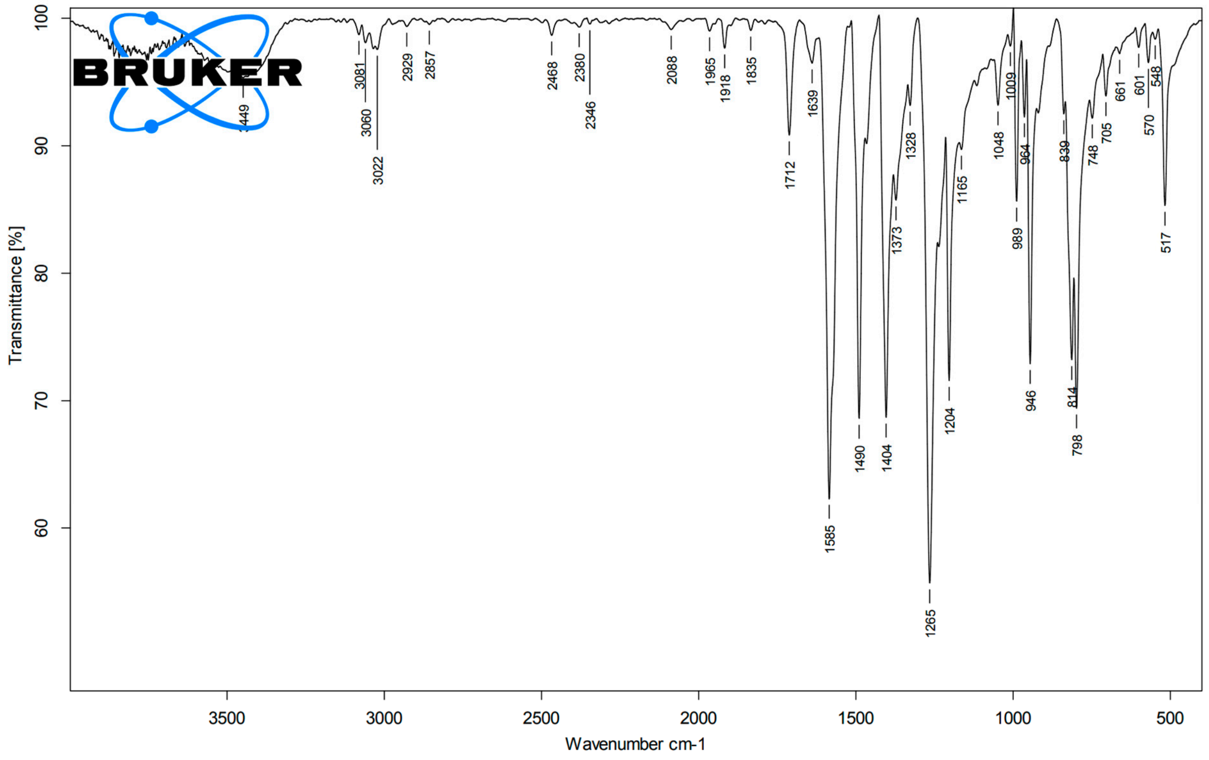

^{1}H, ^{13}C NMR and IR spectra of bis-QACs (6a-g)

^{1}H NMR spectrum of 4,4'-(naphthalene-1,6-diylbis(oxy))bis(1-heptylpyridin-1-ium) dibromide (**6a**)

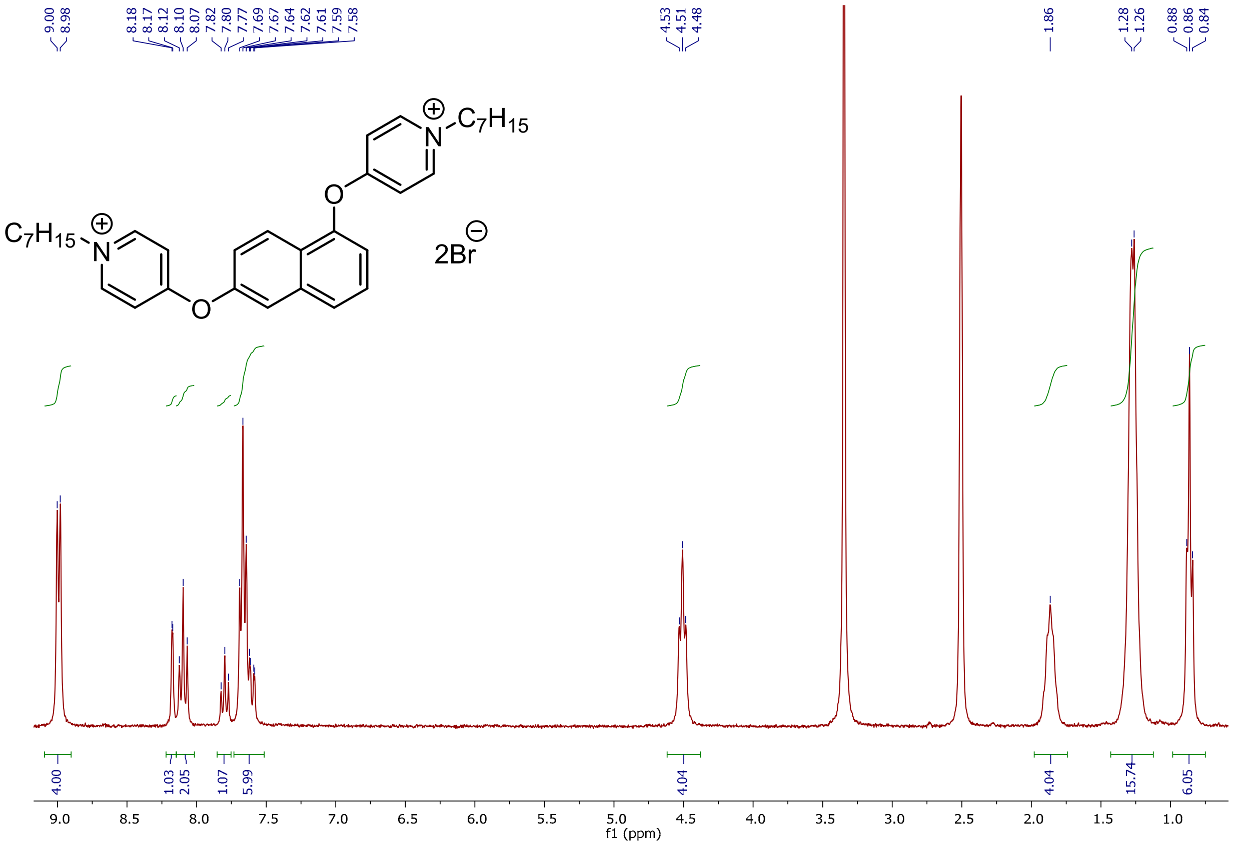

^{13}C NMR spectrum of **6a**

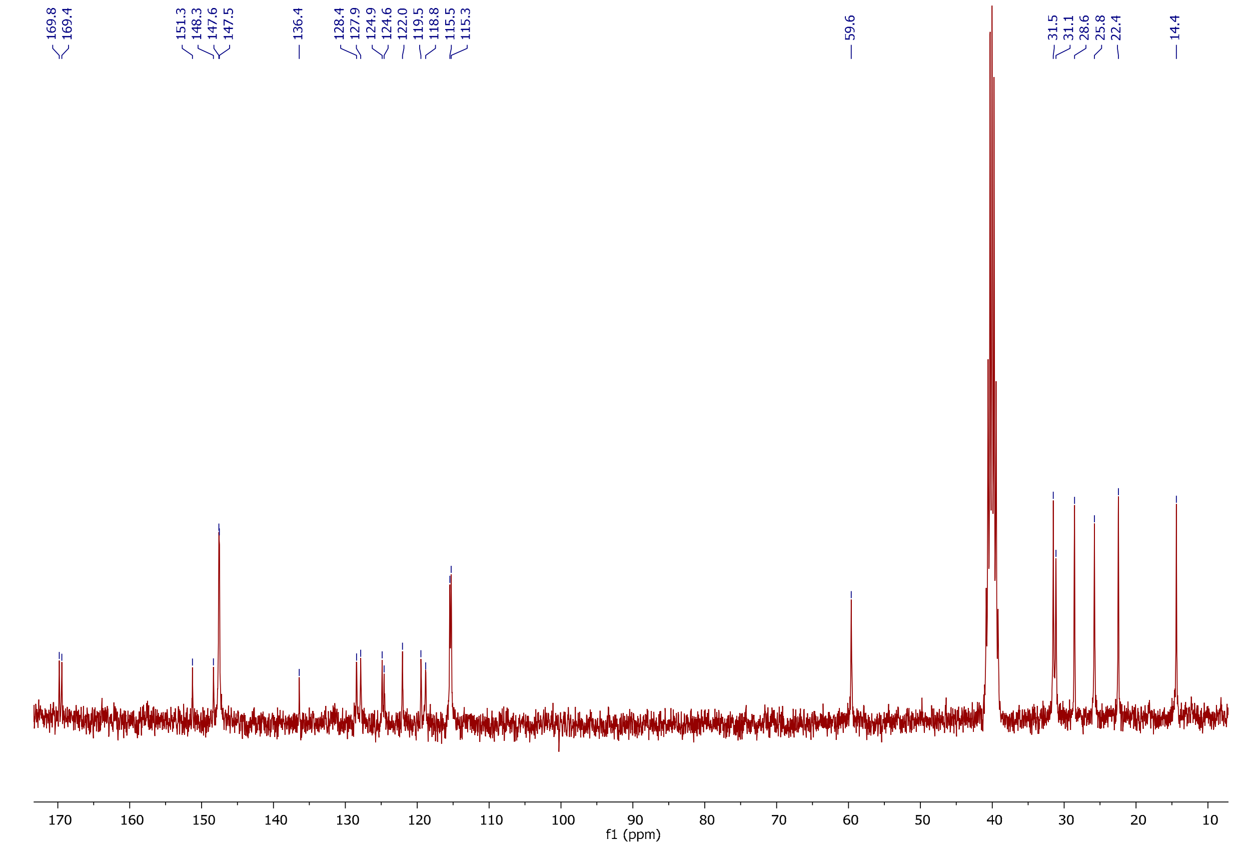

IR spectrum of **6a** (KBr)

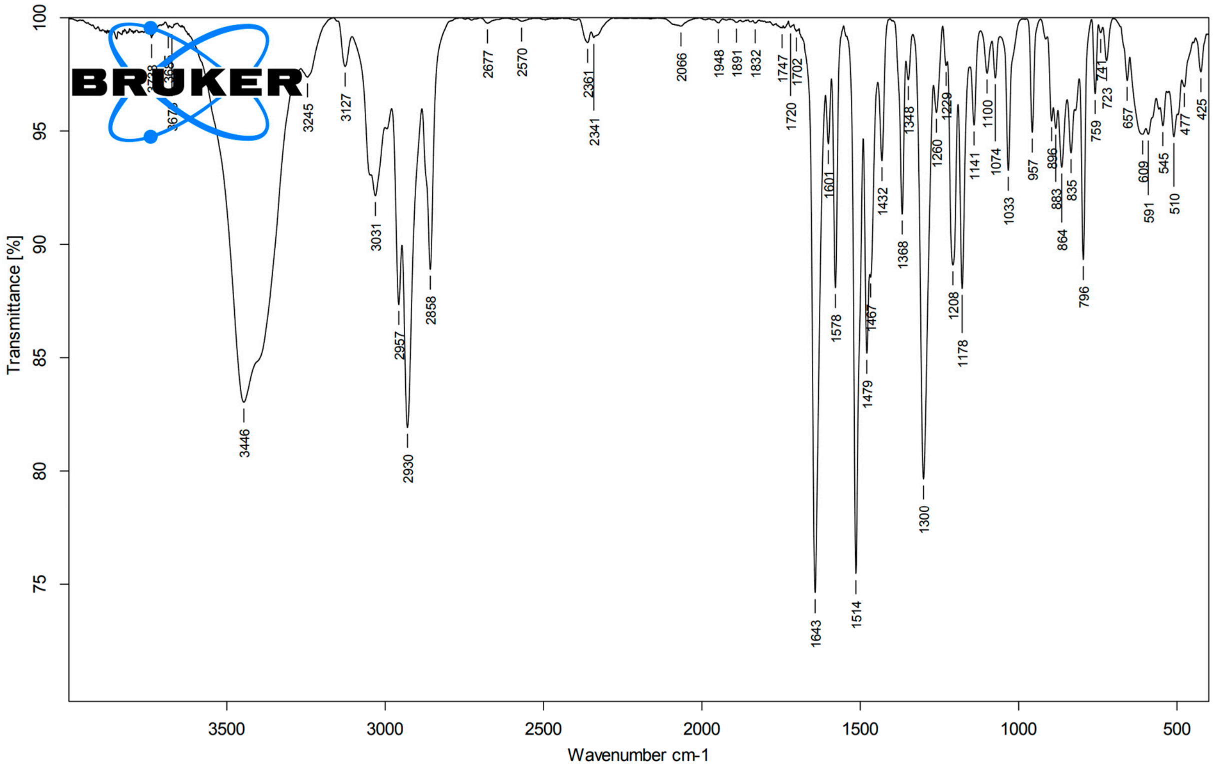

^{1}H NMR spectrum of 4,4'-(naphthalene-1,6-diylbis(oxy))bis(1-octylpyridin-1-ium) dibromide (**6b**)

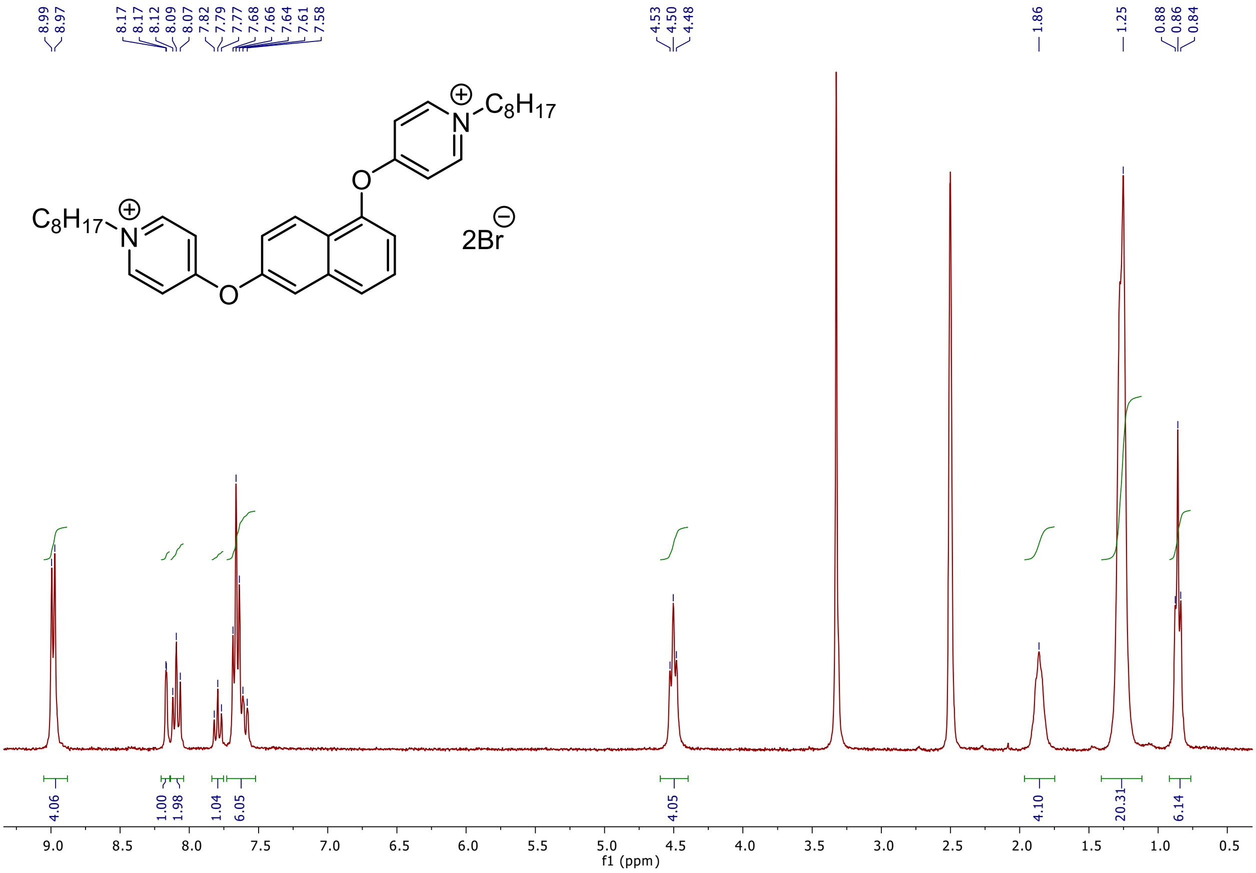

^{13}C NMR spectrum of **6b**

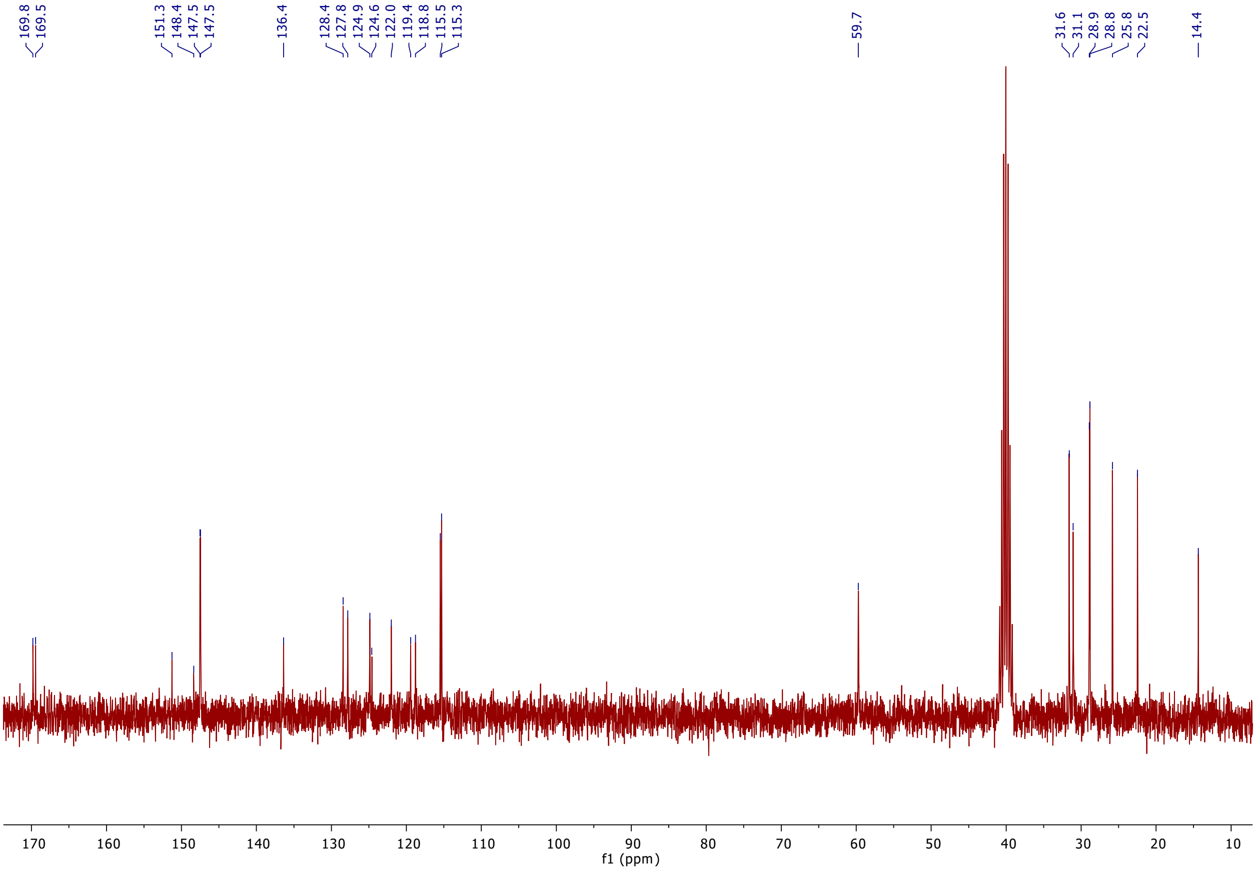

IR spectrum of **6b** (KBr)

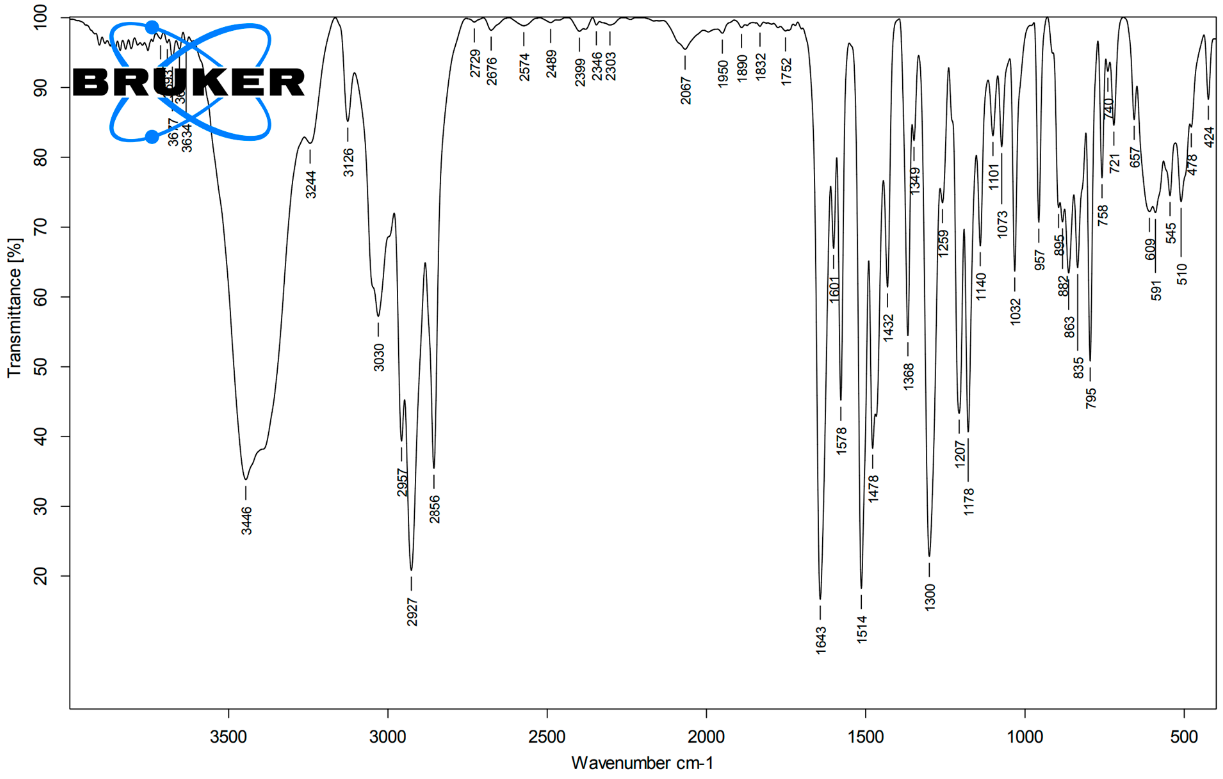

^{1}H NMR spectrum of 4,4'-(naphthalene-1,6-diylbis(oxy))bis(1-decylpyridin-1-ium) diiodide (**6c**)

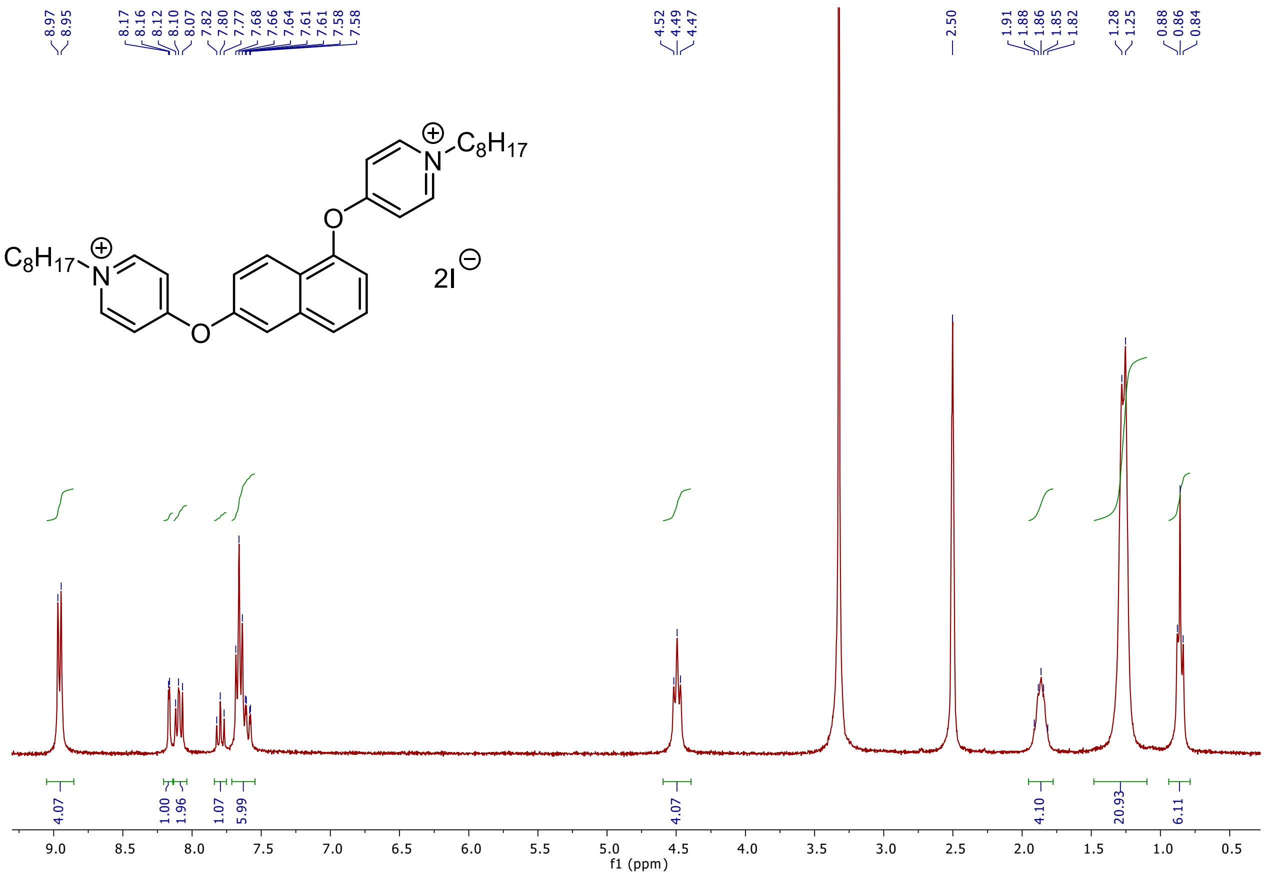

IR spectrum of **6c** (KBr)

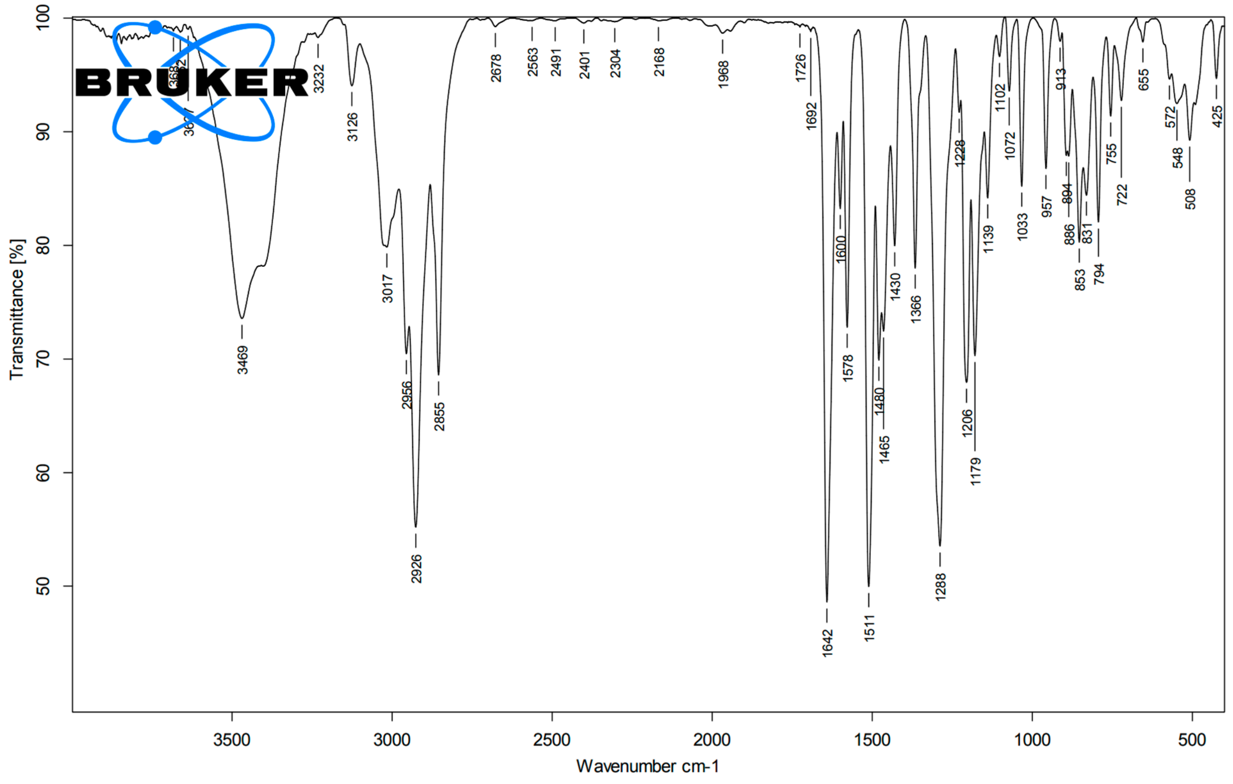

^{1}H NMR spectrum of 4,4'-(naphthalene-1,6-diylbis(oxy))bis(1-nonylpyridin-1-ium) dibromide (**6d**)

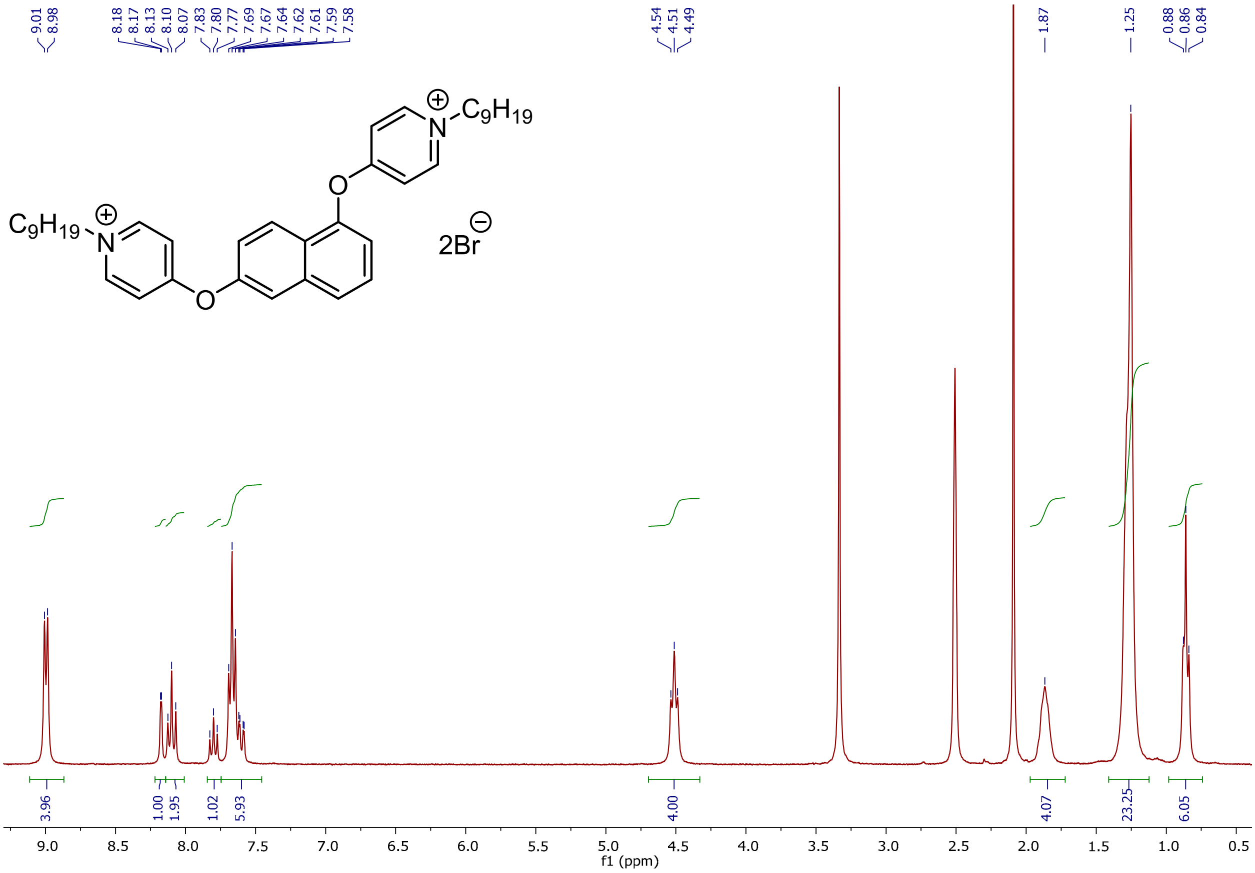

^{13}C NMR spectrum of **6d**

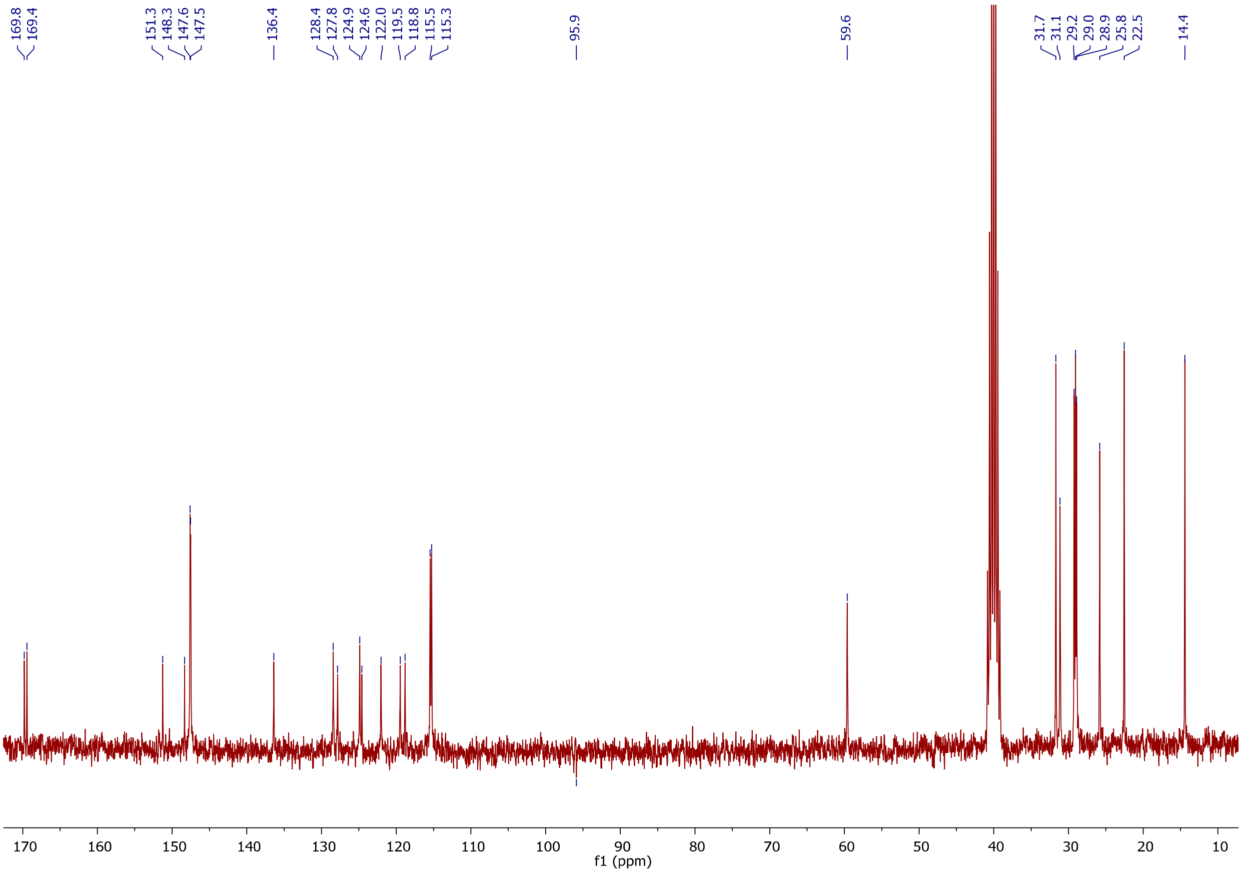

IR spectrum of **6d** (KBr)

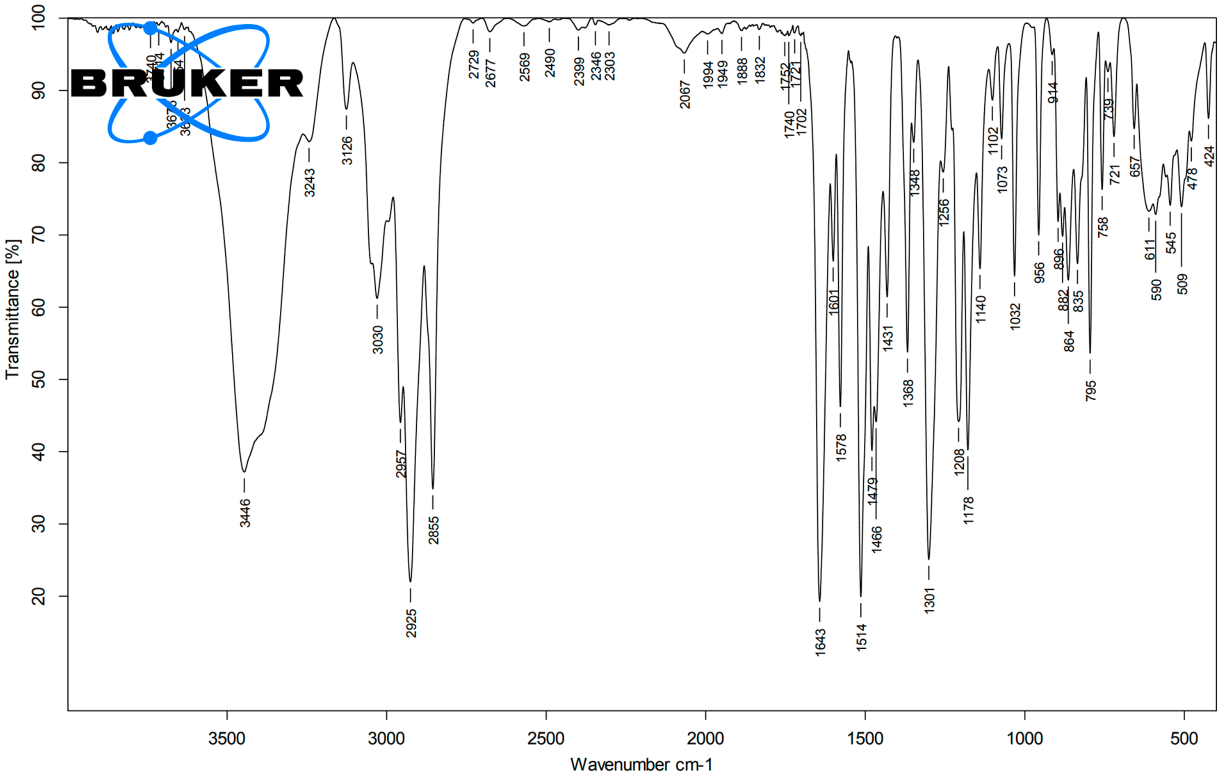

^{1}H NMR spectrum of 4,4'-(naphthalene-1,6-diylbis(oxy))bis(1-decylpyridin-1-ium) dibromide (**6e**)

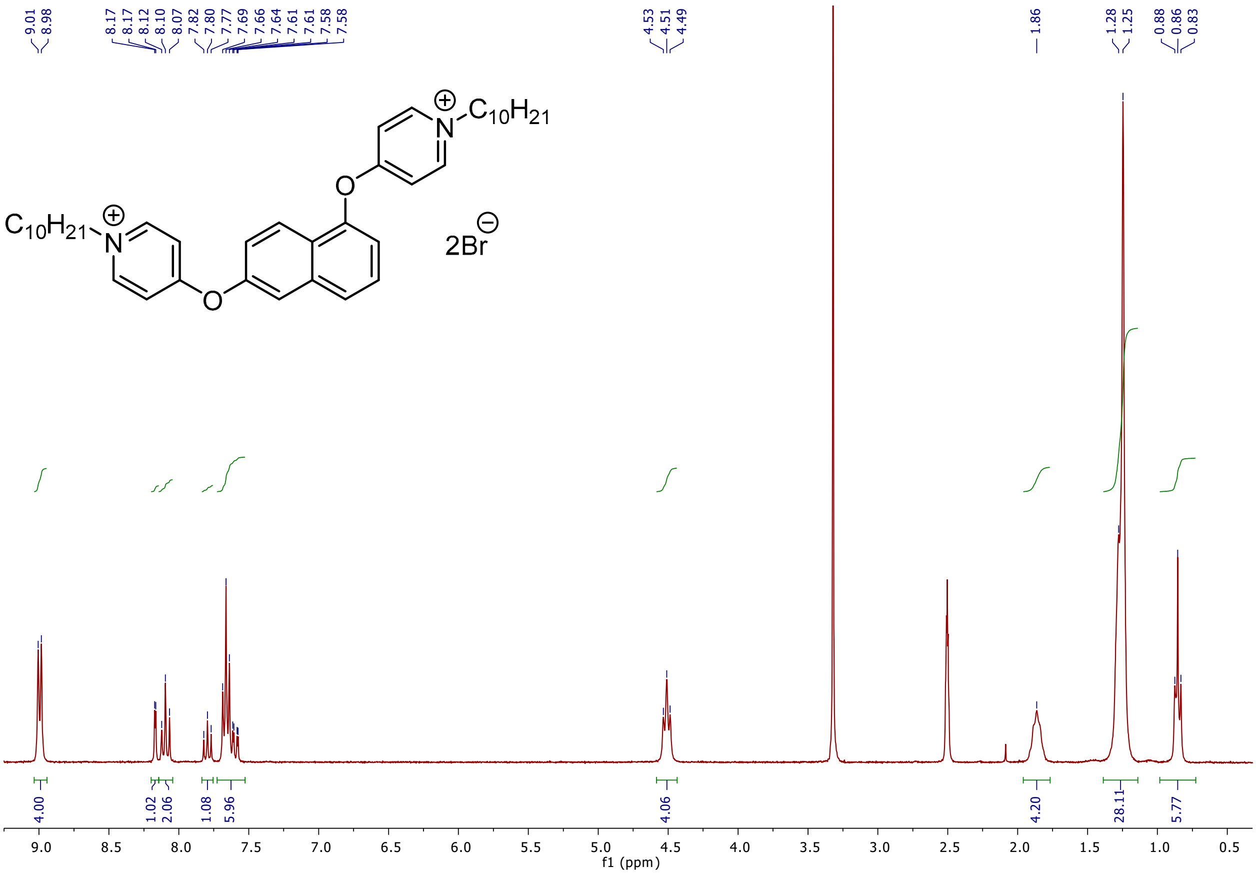

^{13}C NMR spectrum of **6e**

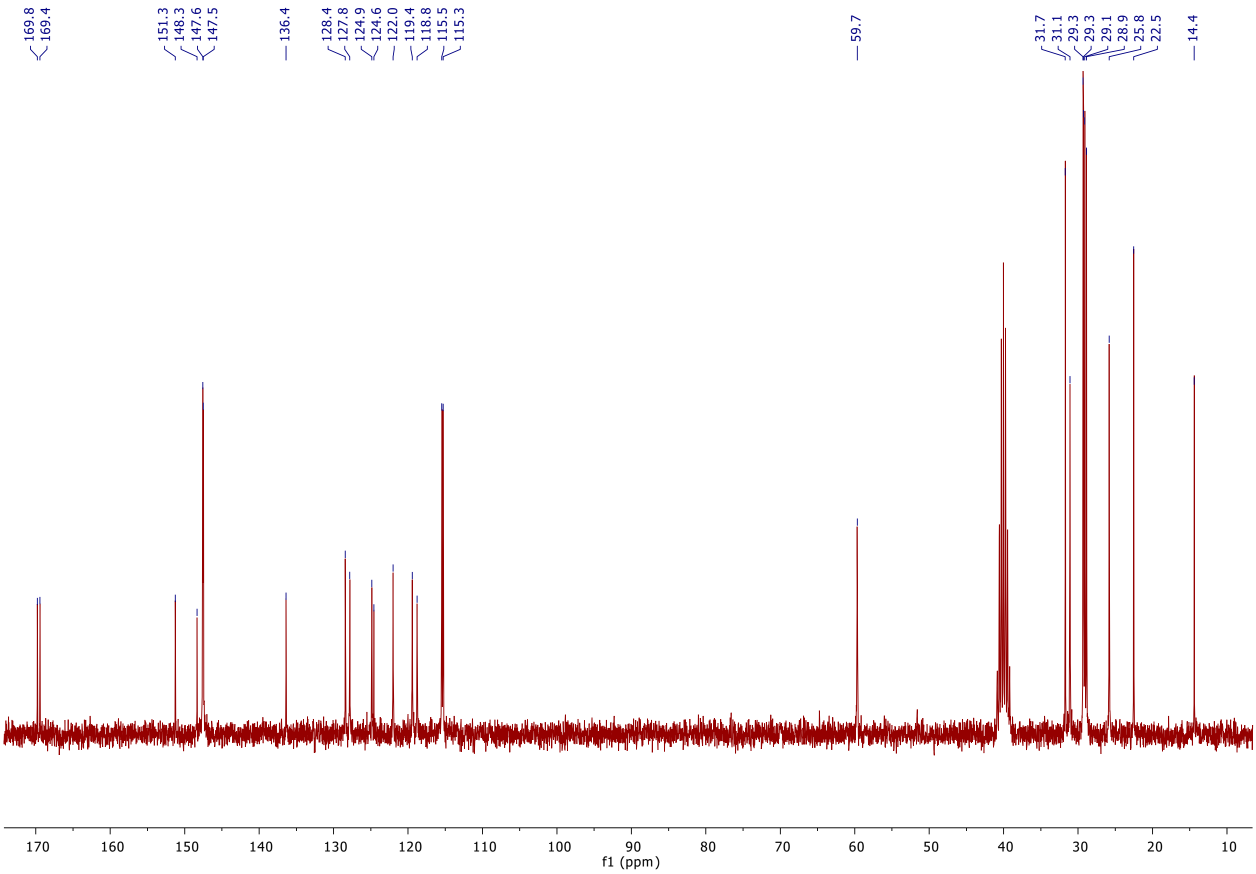

IR spectrum of **6e** (KBr)

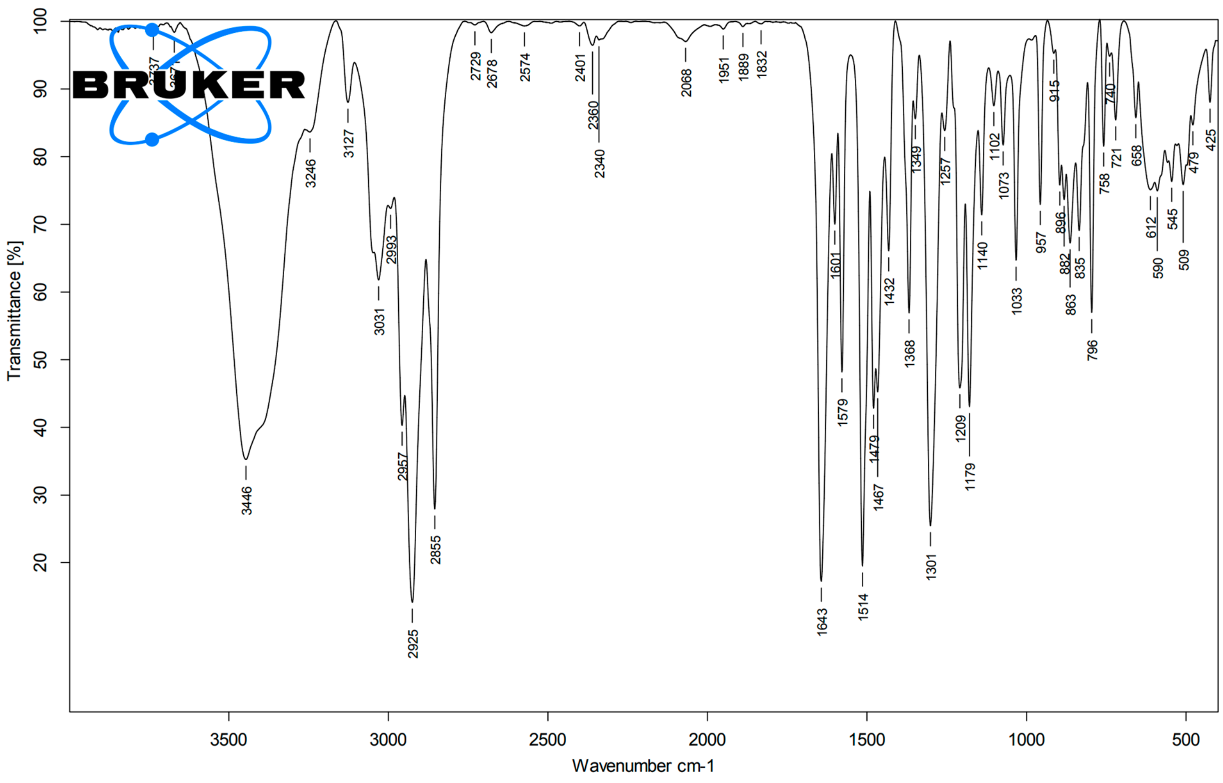

^{1}H NMR spectrum of 4,4'-(naphthalene-1,6-diylbis(oxy))bis(1-undecylpyridin-1-ium) dibromide (**6f**)

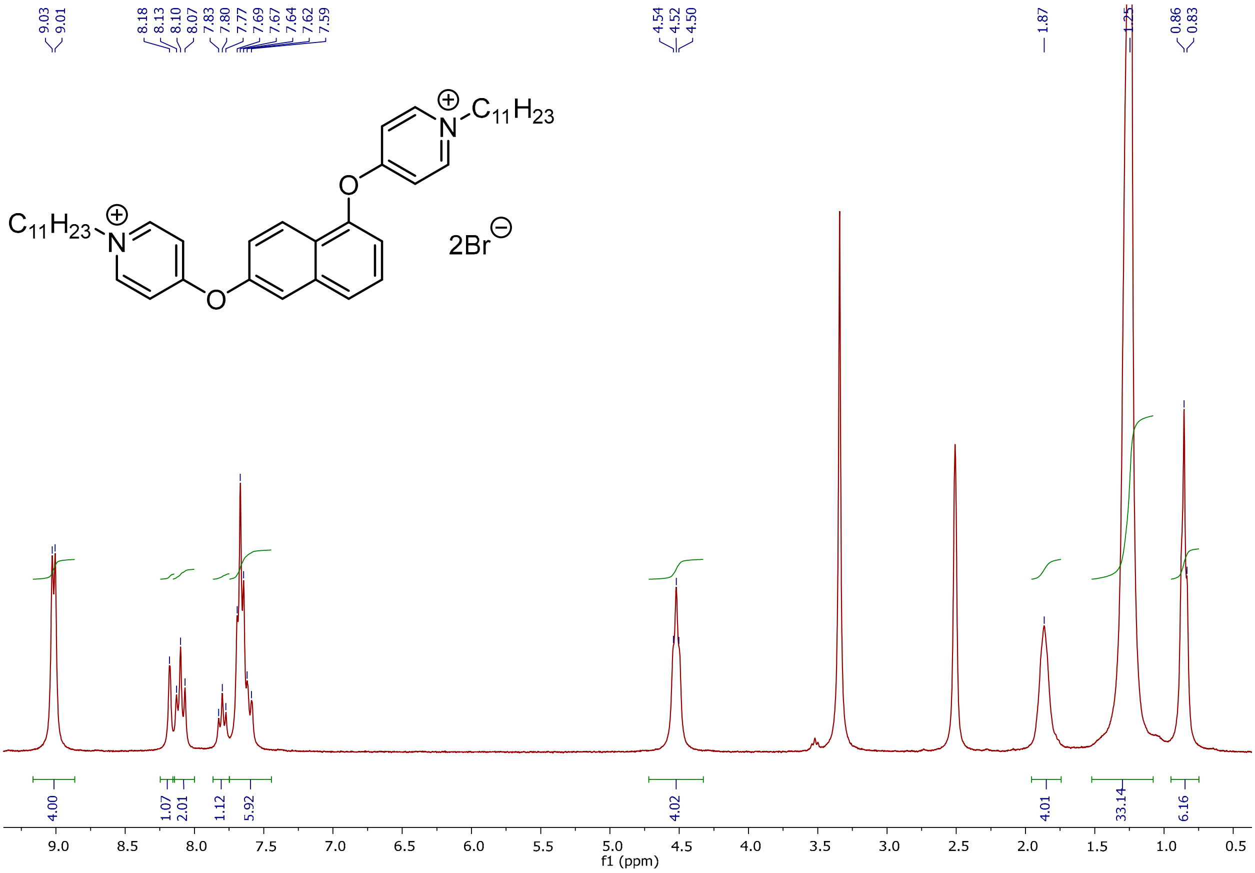

^{13}C NMR spectrum of **6f**

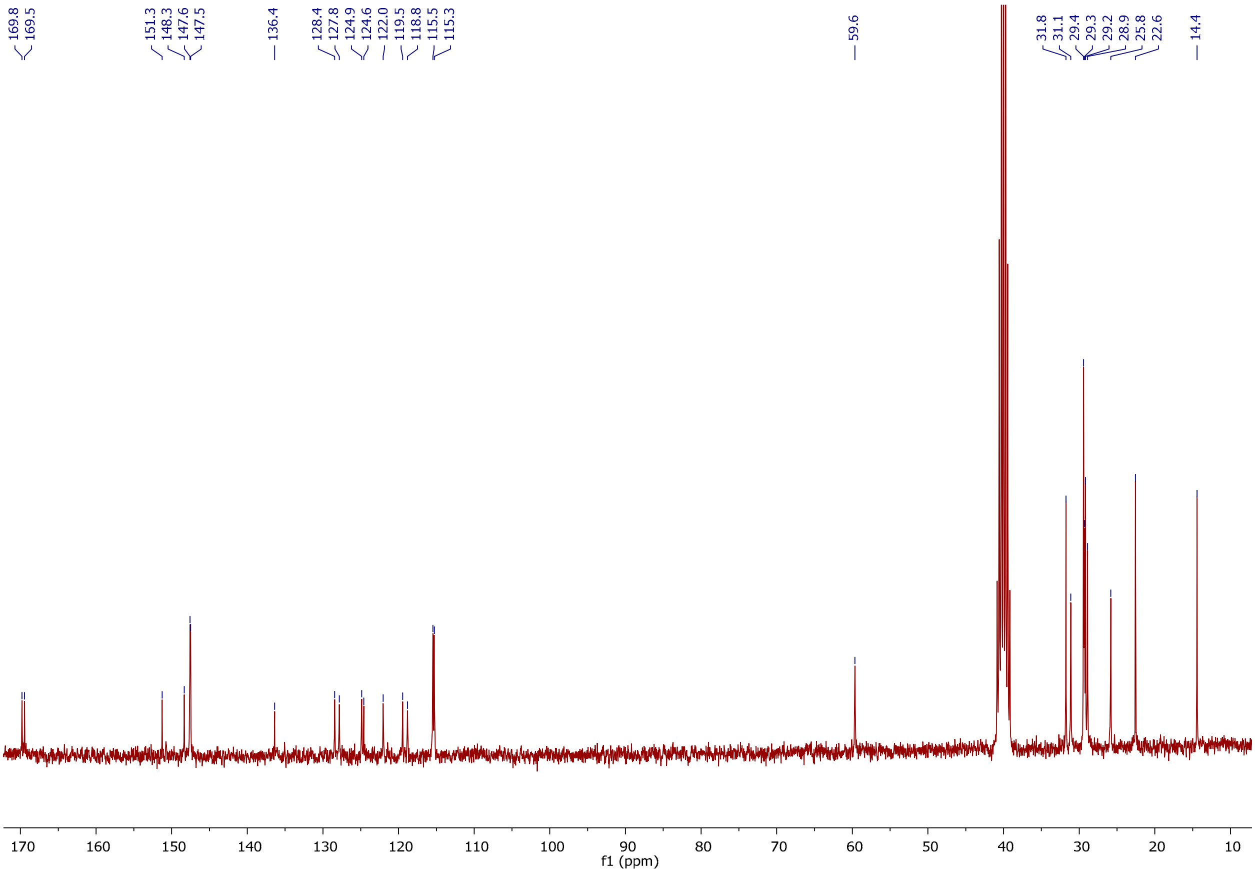

IR spectrum of **6f** (KBr)

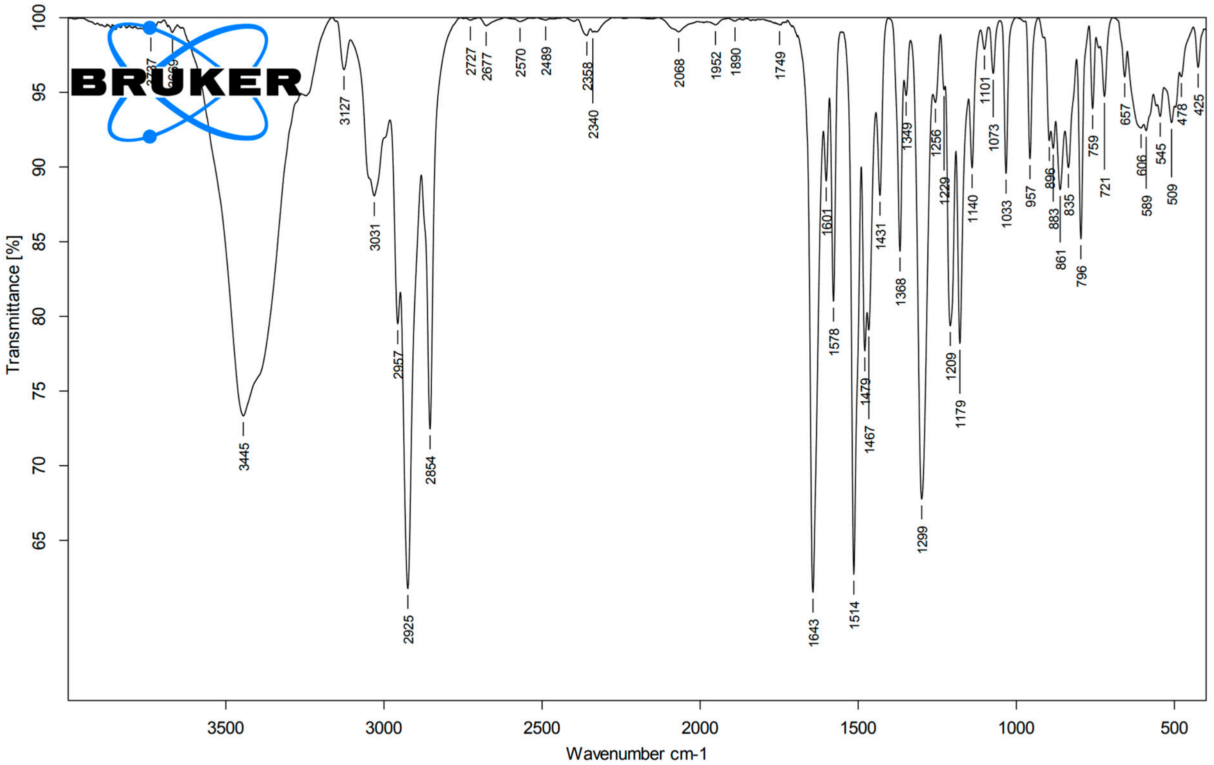

^{1}H NMR spectrum of 4,4'-(naphthalene-1,6-diylbis(oxy))bis(1-dodecylpyridin-1-ium) dibromide **(6g)**

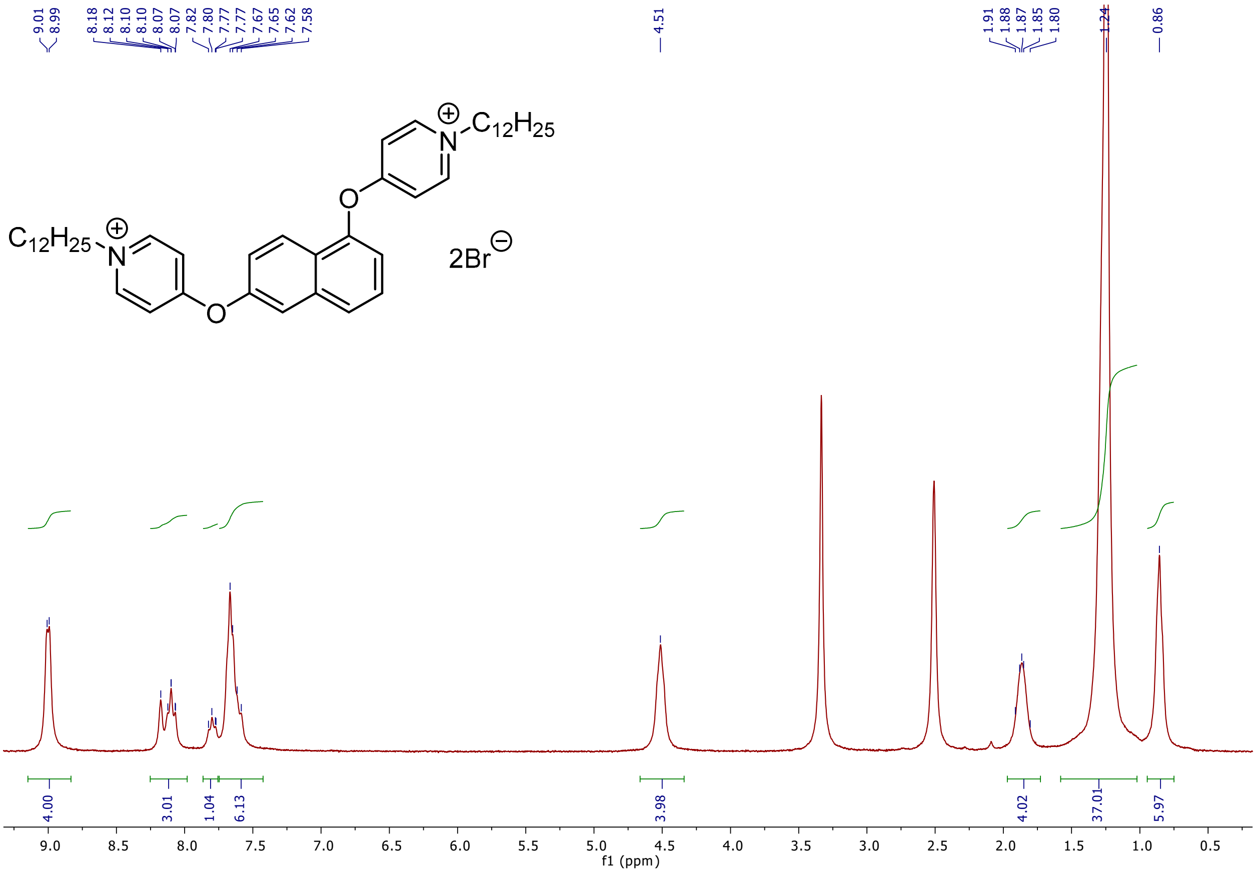

^{13}C NMR spectrum of **6g**

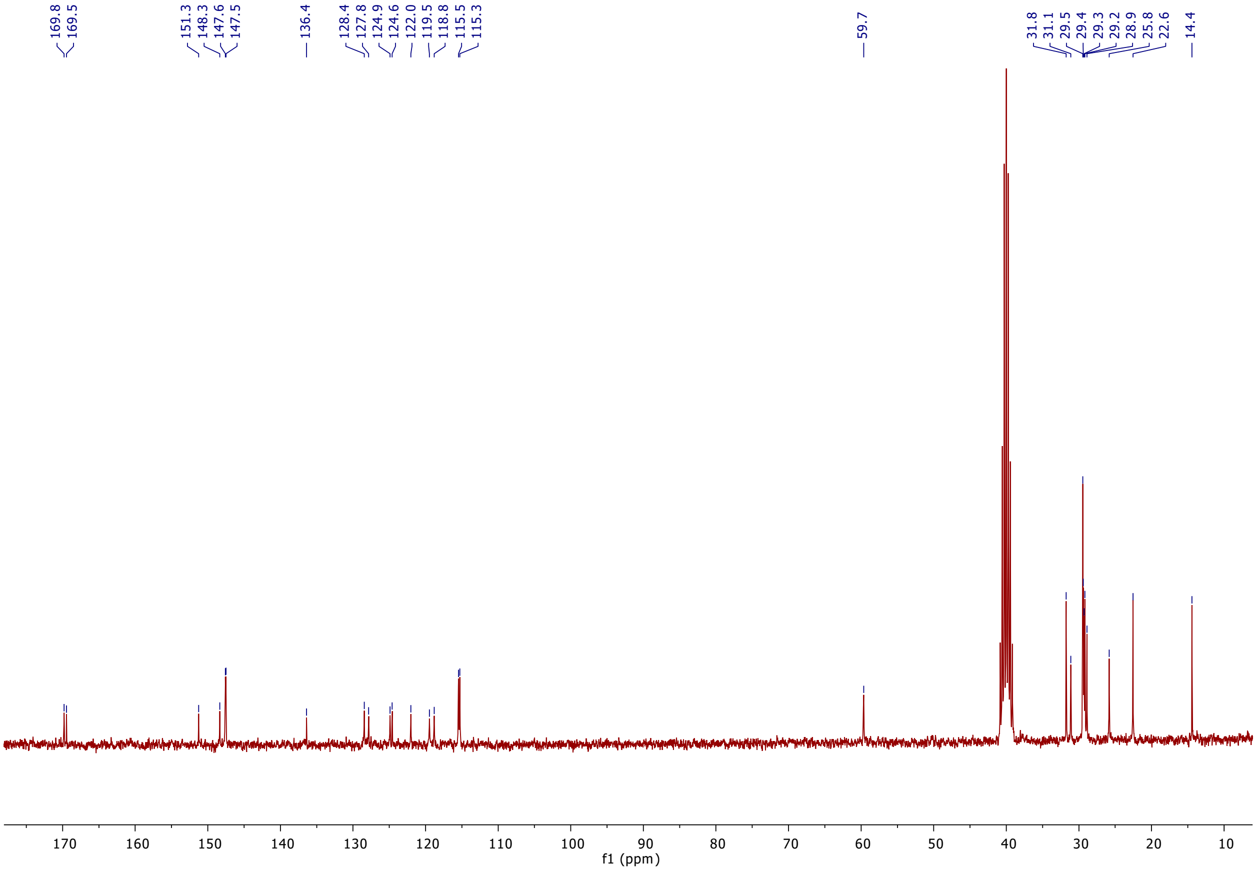

IR spectrum of **6g** (KBr)

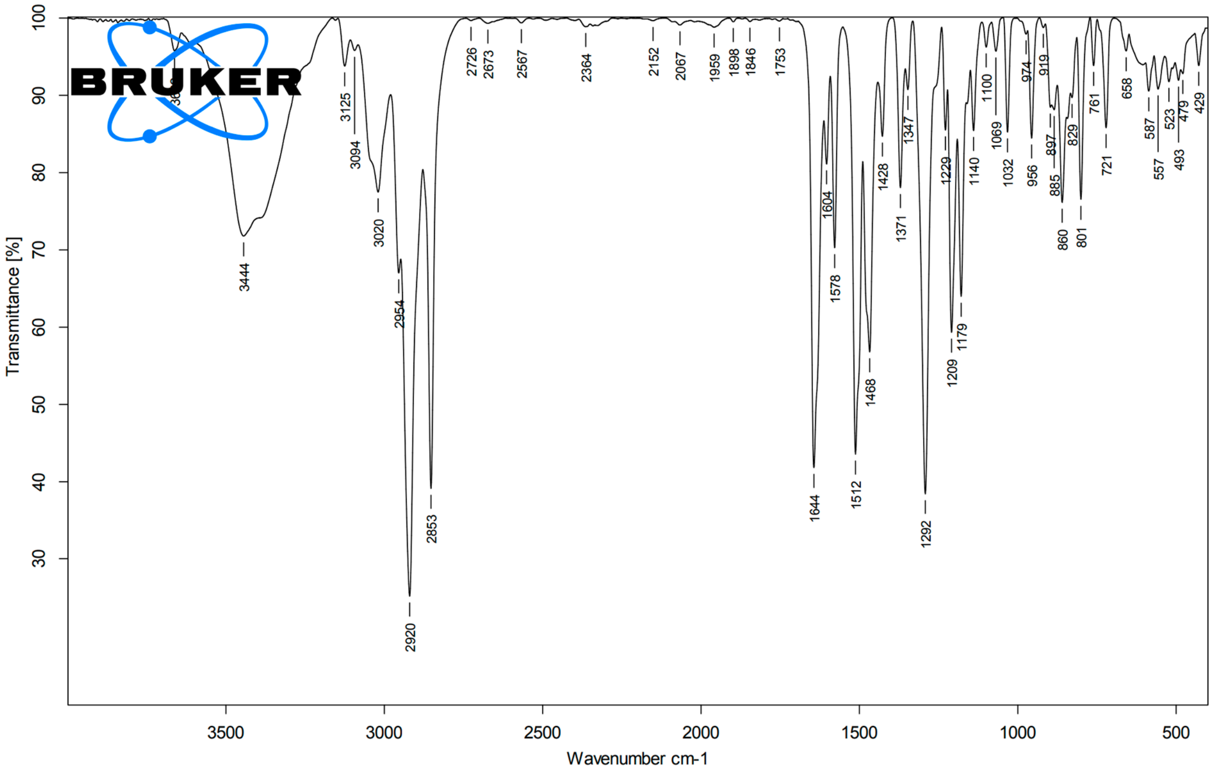

^{1}H and ^{13}C NMR spectra of bis-QACs (7a-f)

^{1}H NMR spectrum of 4,4'-(naphthalene-1,5-diylbis(oxy))bis(1-heptylpyridin-1-ium) dibromide (**7a**)

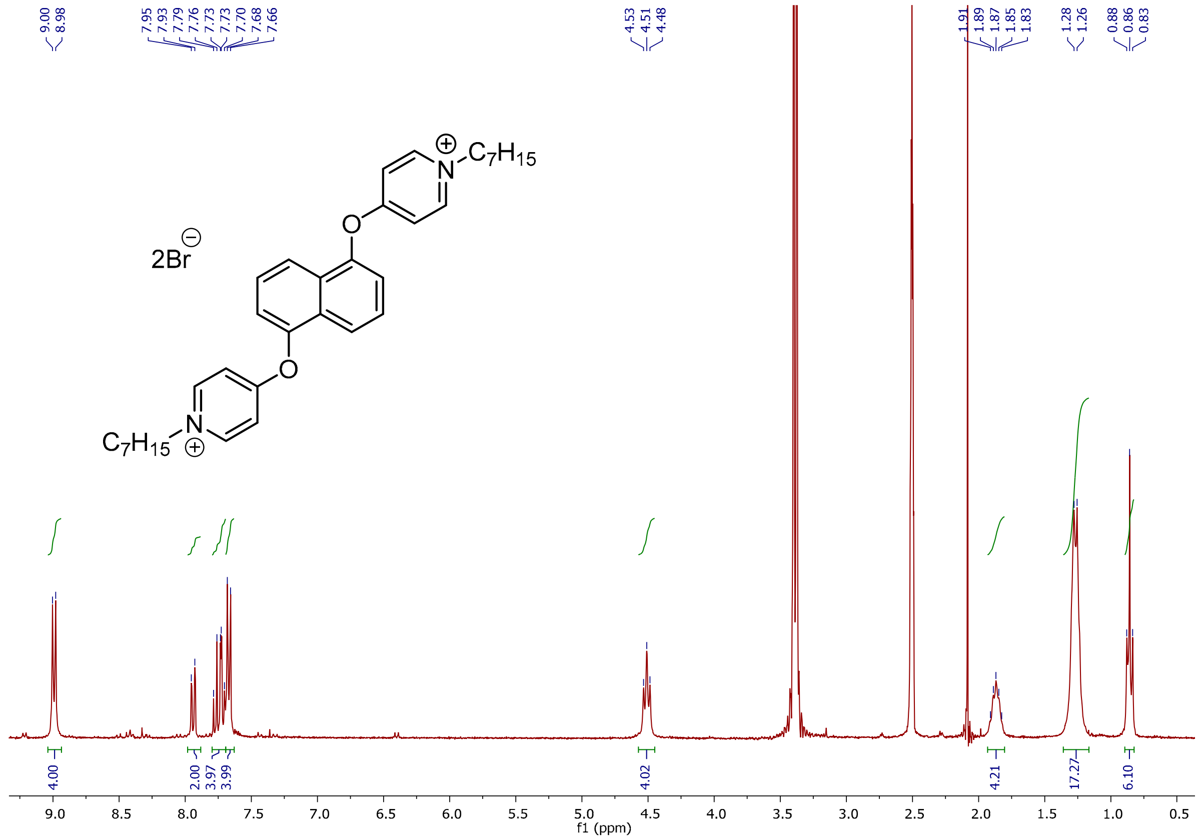

^{13}C NMR spectrum of **7a**

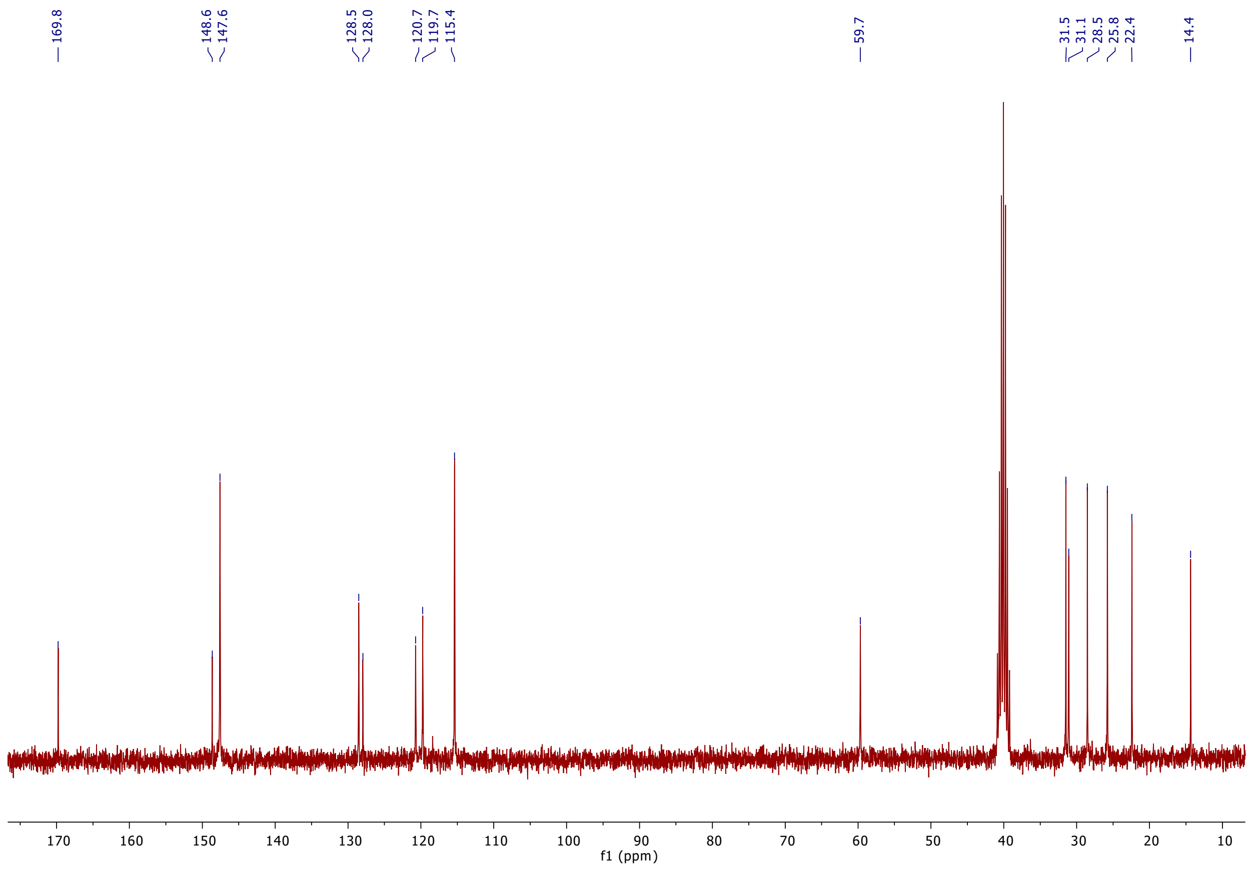

IR spectrum of **7a** (KBr)

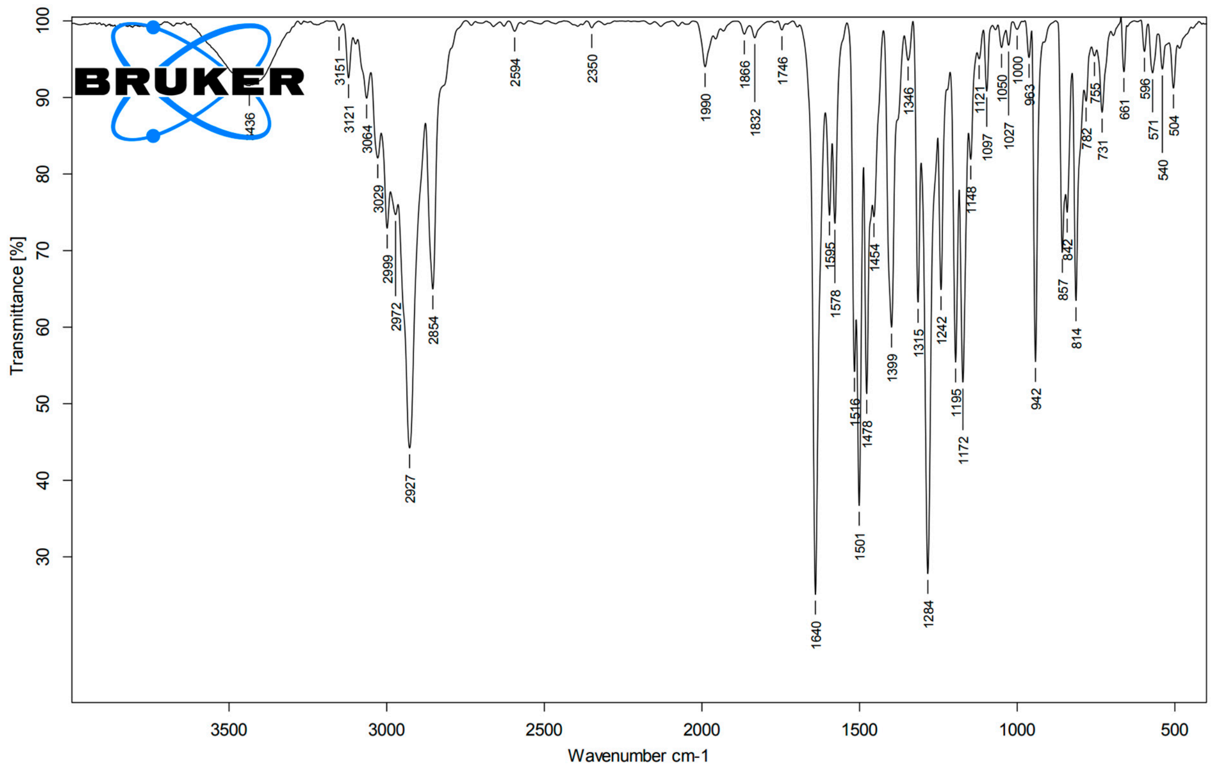

^{1}H NMR spectrum of 4,4'-(naphthalene-1,5-diylbis(oxy))bis(1-octylpyridin-1-ium) dibromide (**7b**)

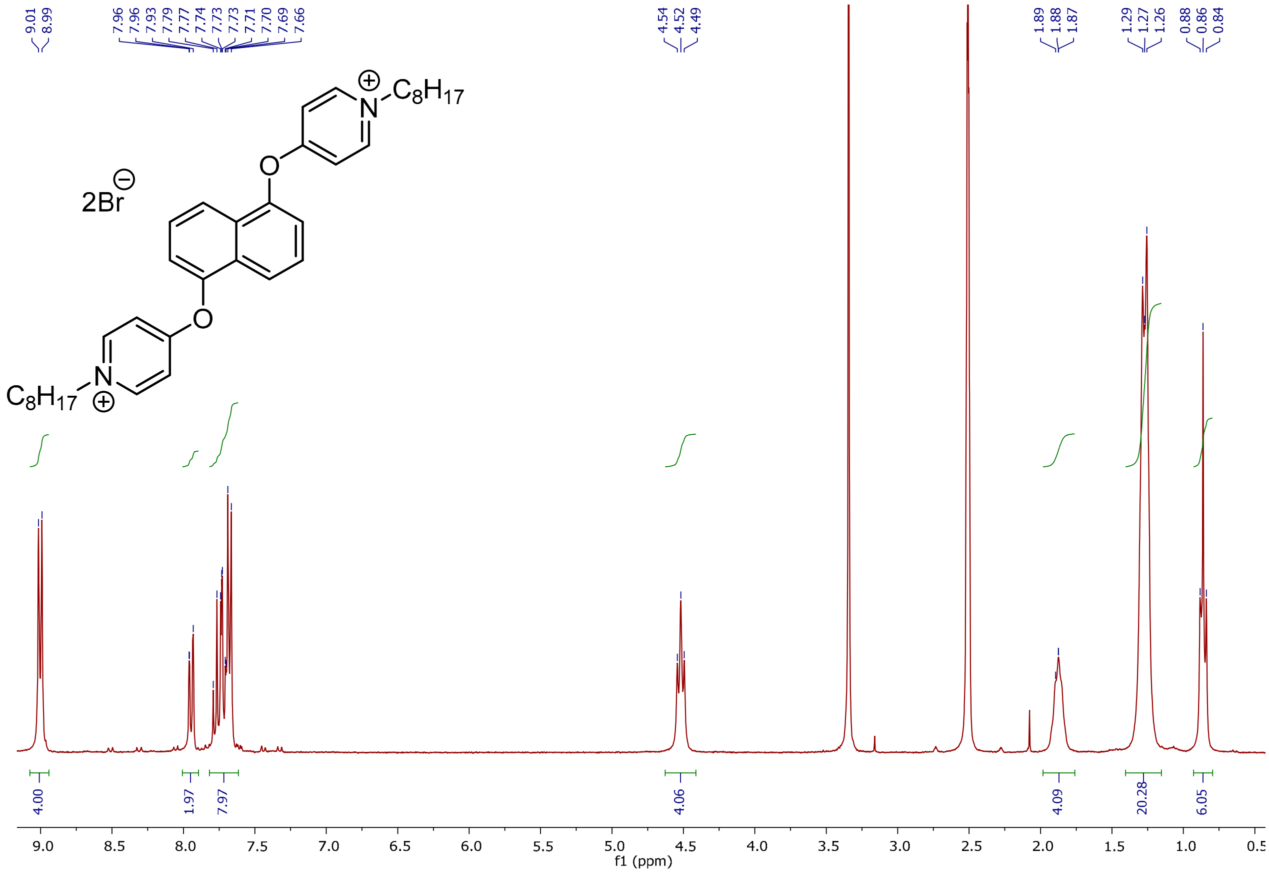

^{13}C NMR spectrum of **7b**

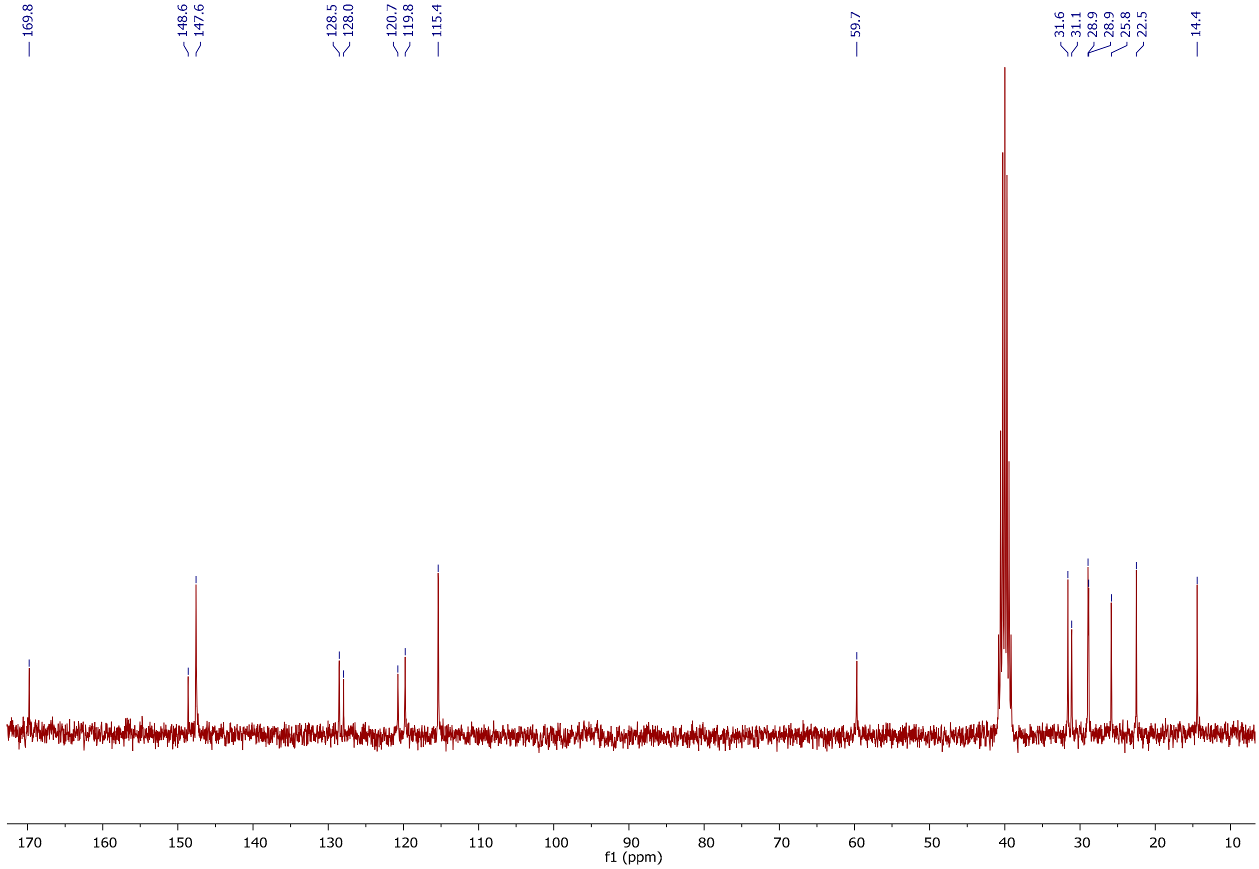

IR spectrum of **7b** (KBr)

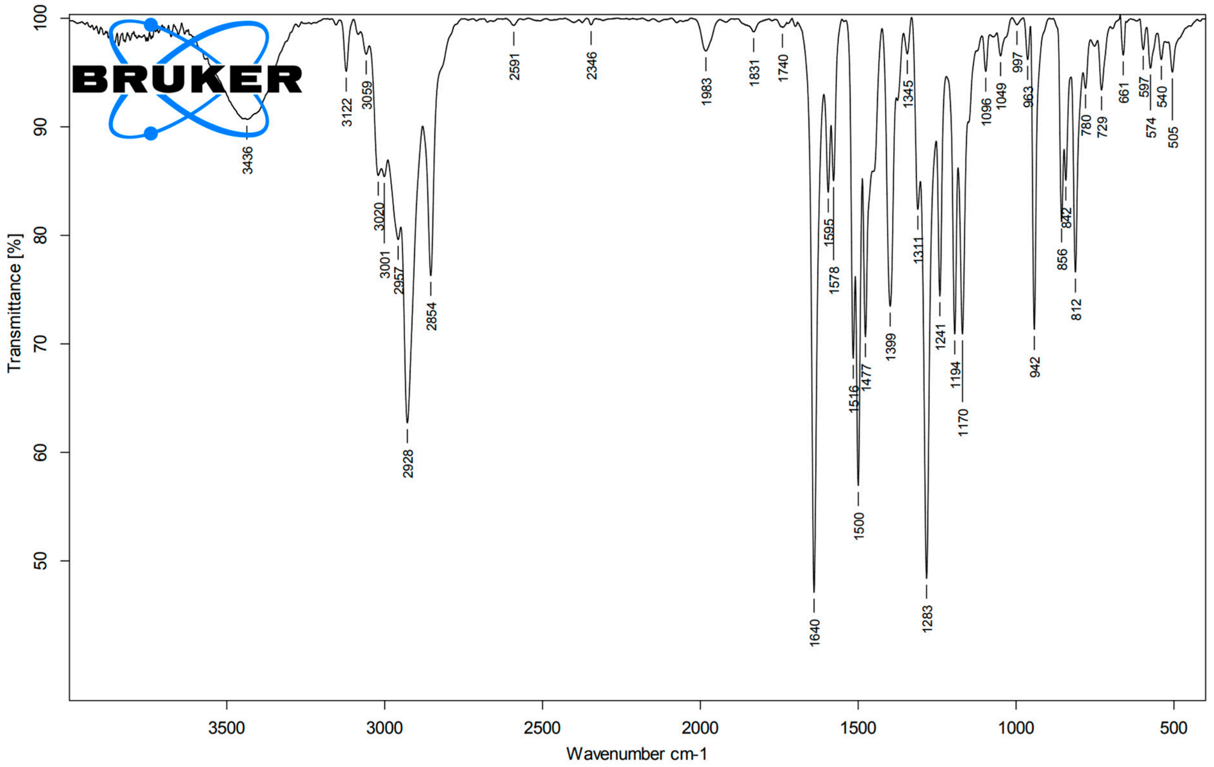

^{1}H NMR spectrum of 4,4'-(naphthalene-1,5-diylbis(oxy))bis(1-nonylpyridin-1-ium) dibromide (**7c**)

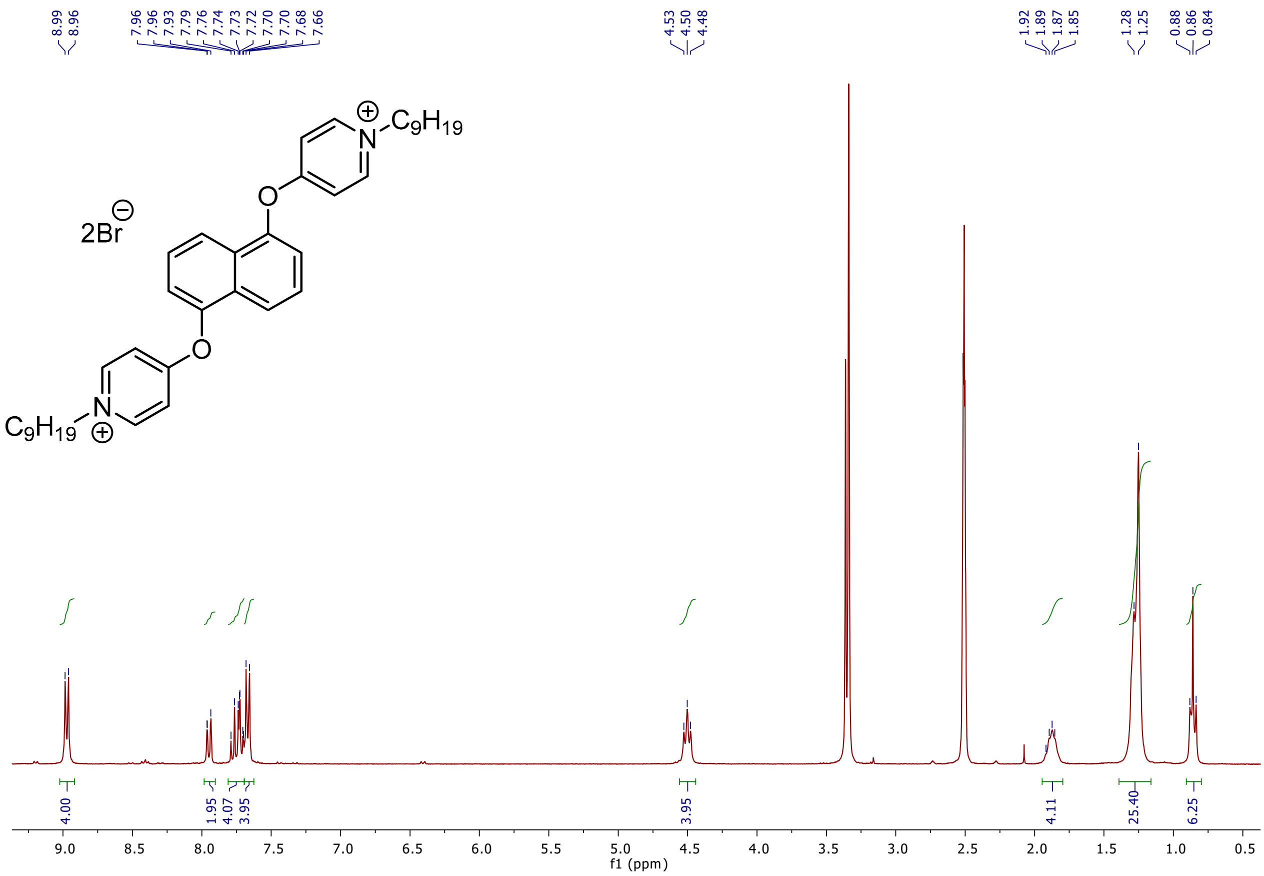

^{13}C NMR spectrum of **7c**

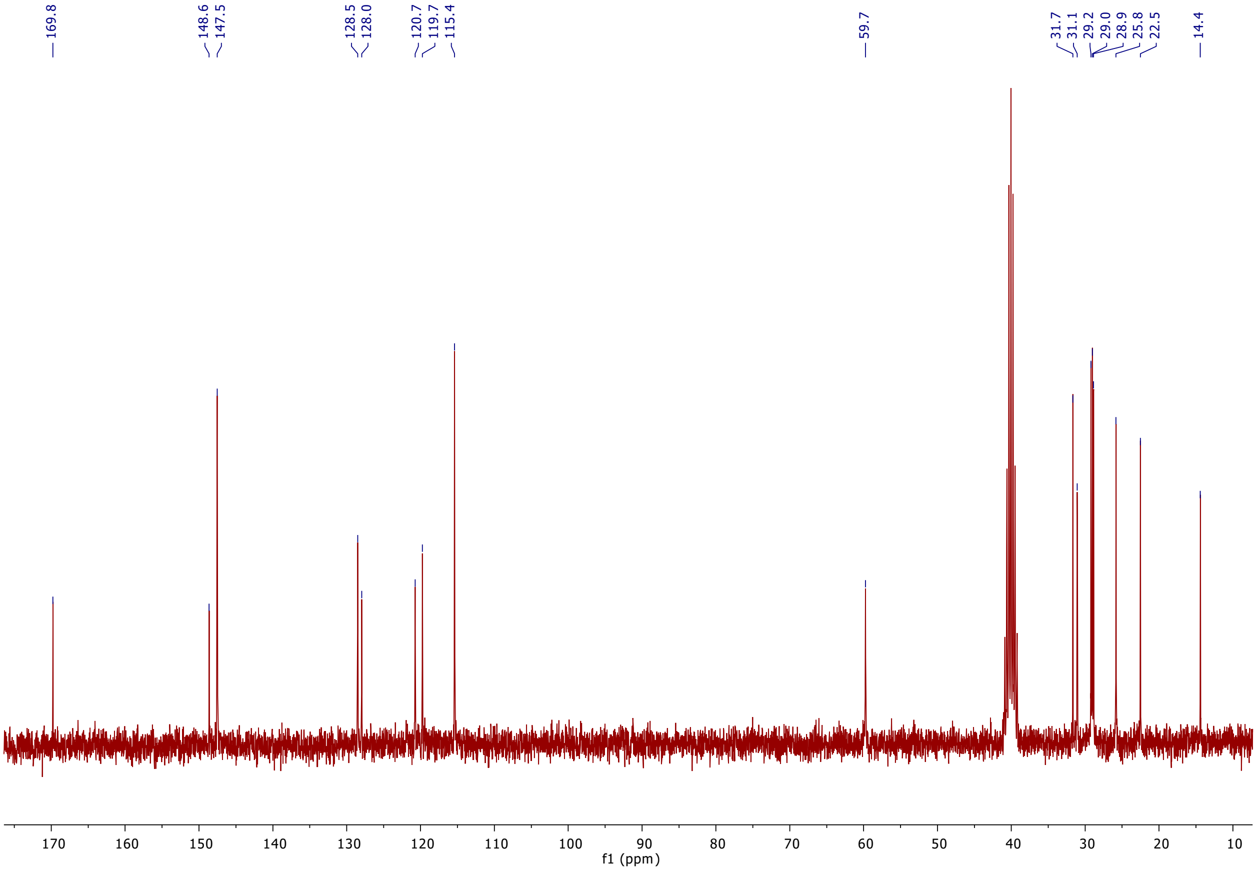

IR spectrum of **7c** (KBr)

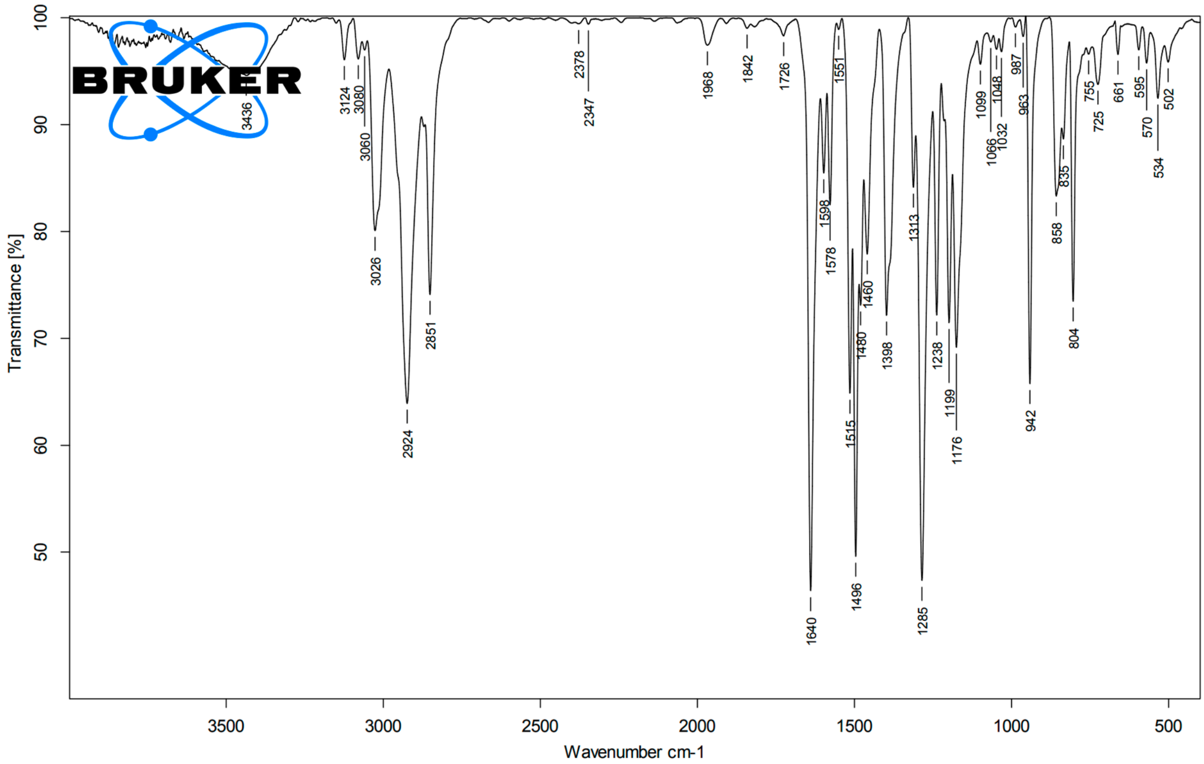

^{1}H NMR spectrum of 4,4'-(naphthalene-1,5-diylbis(oxy))bis(1-decylpyridin-1-ium) dibromide (**7d**)

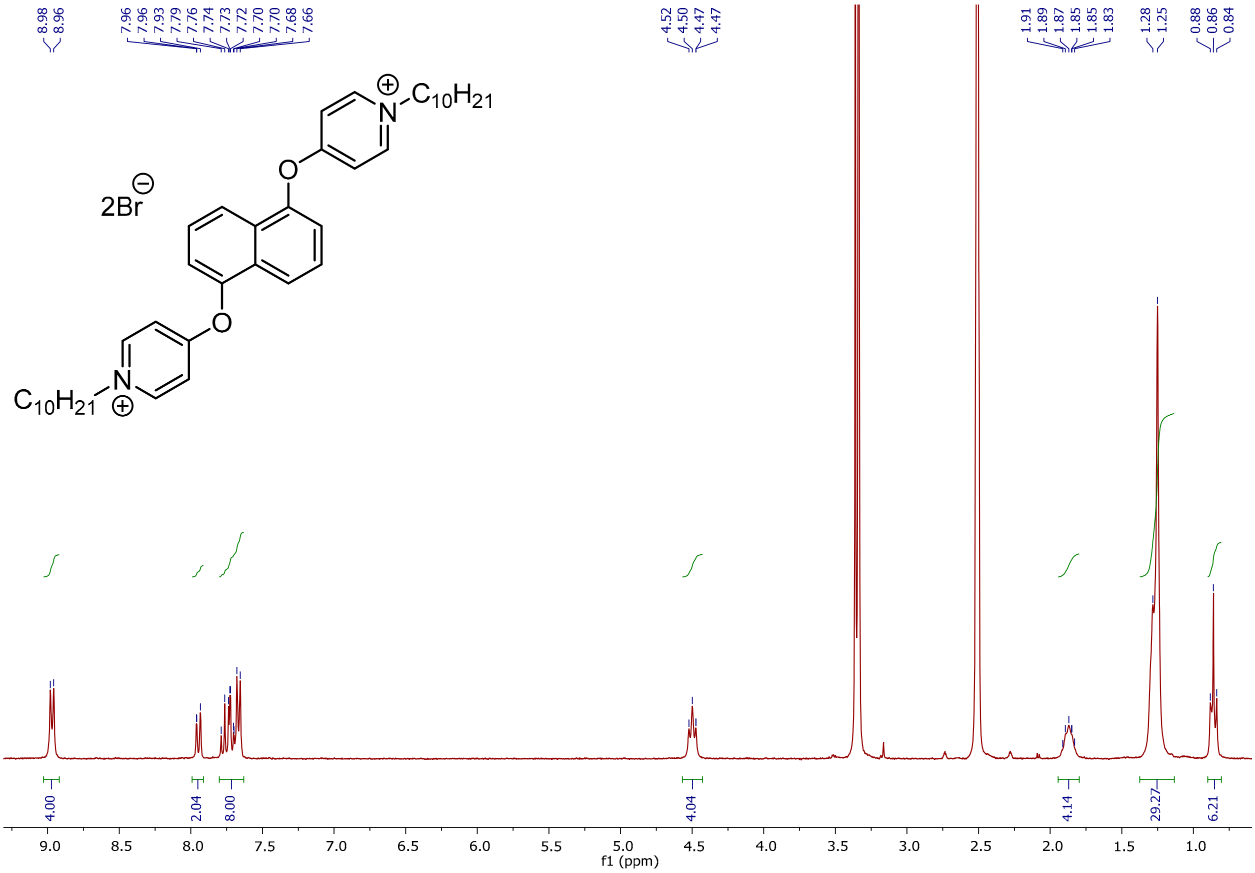

^{13}C NMR spectrum of **7d**

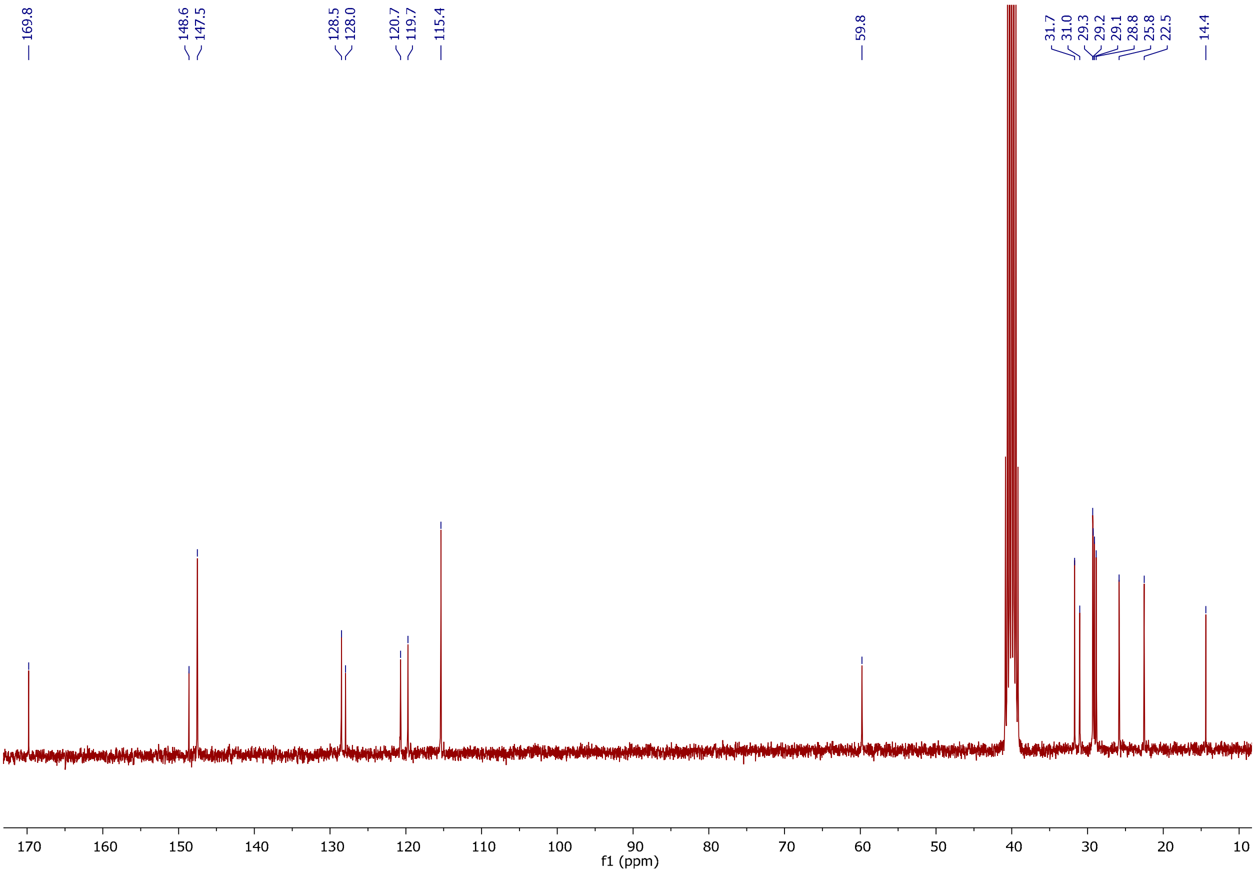

^{1}H NMR spectrum of 4,4'-(naphthalene-1,5-diylbis(oxy))bis(1-undecylpyridin-1-ium) dibromide (**7e**)

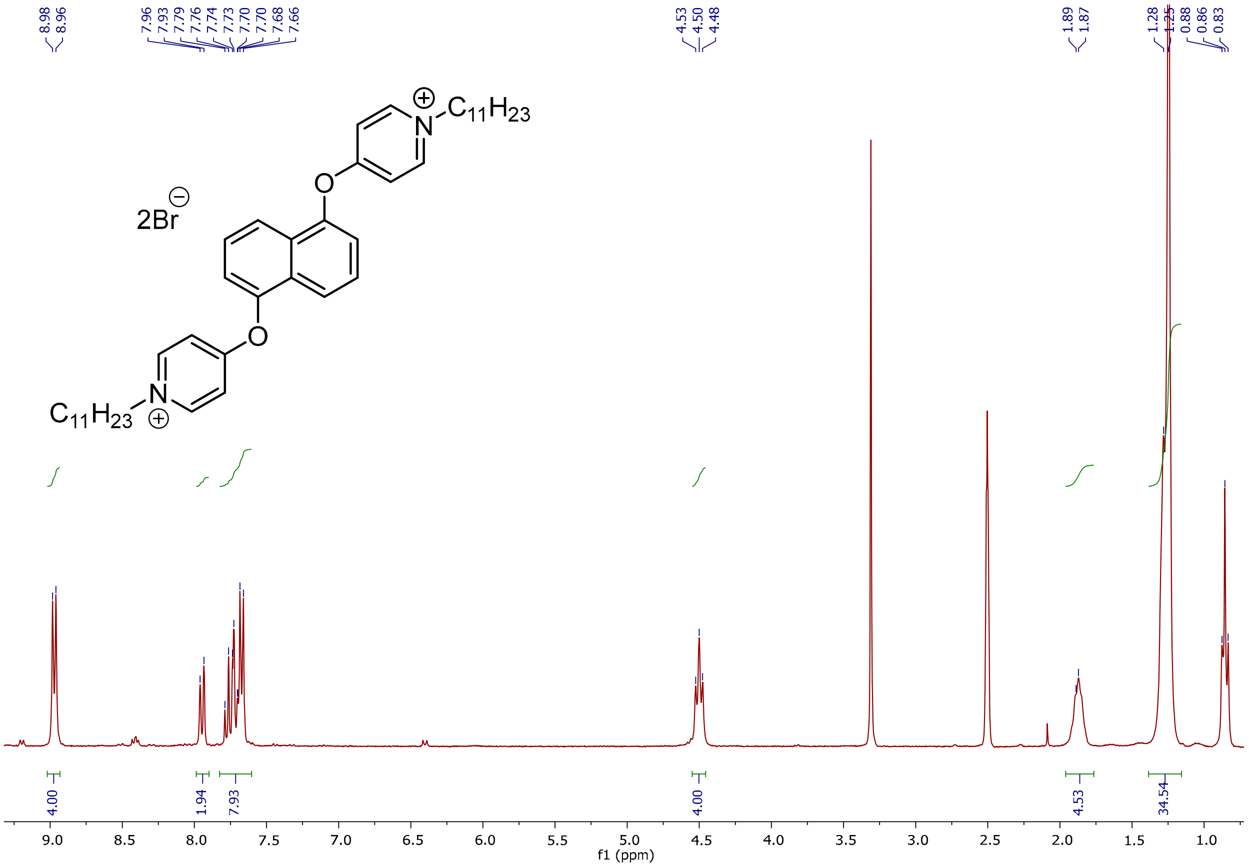

^{1}H NMR spectrum of 4,4'-(naphthalene-1,5-diylbis(oxy))bis(1-dodecylpyridin-1-ium) dibromide (**7f**)

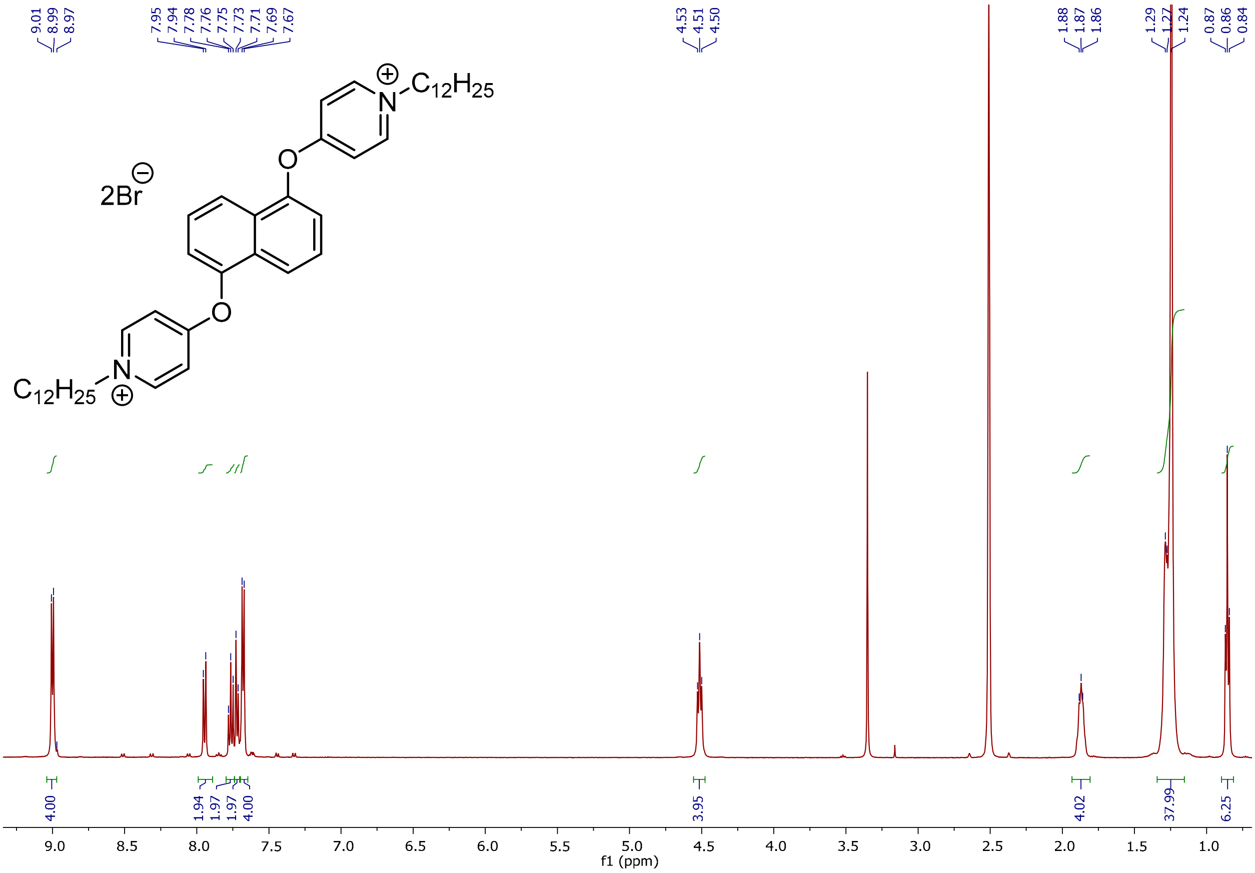

^{13}C NMR spectrum of **7f**

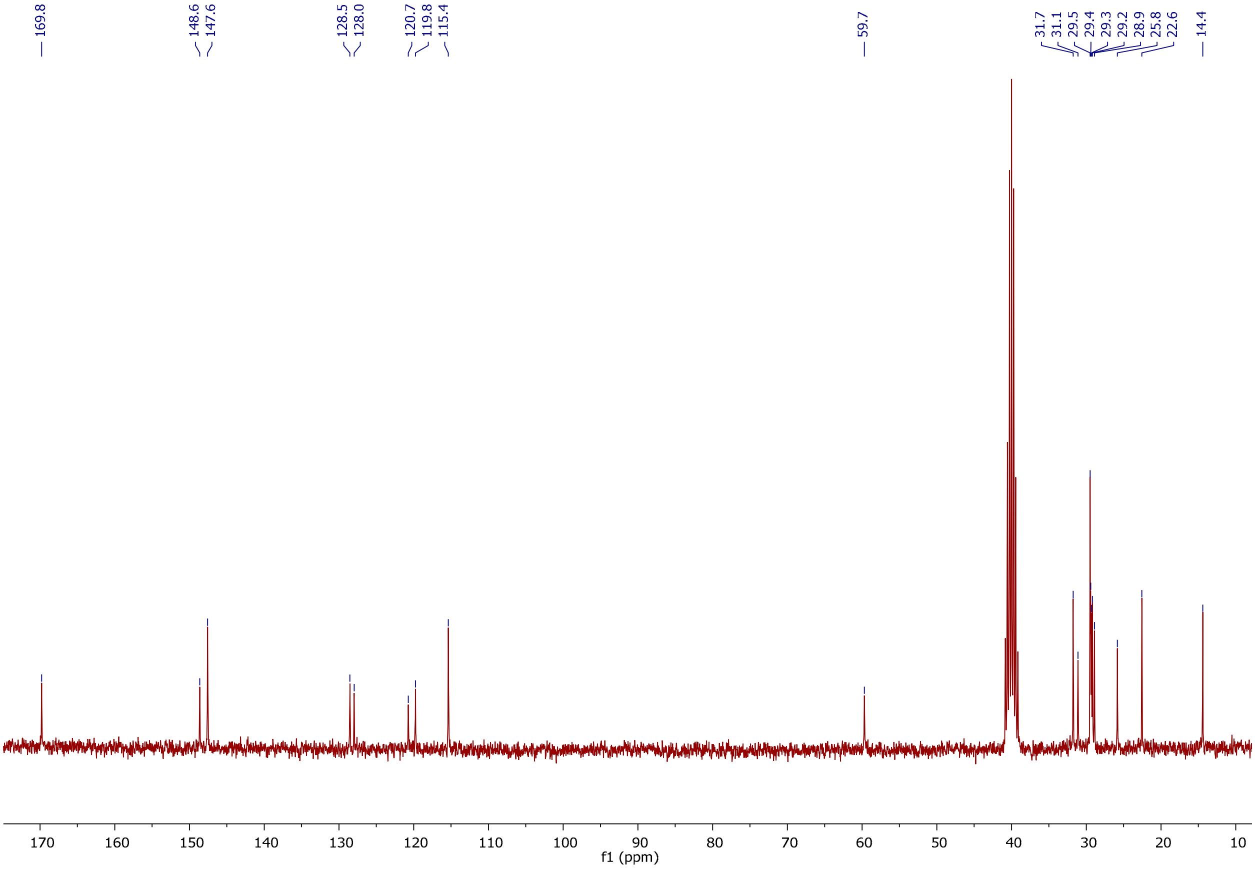

IR spectrum of **7f** (KBr)

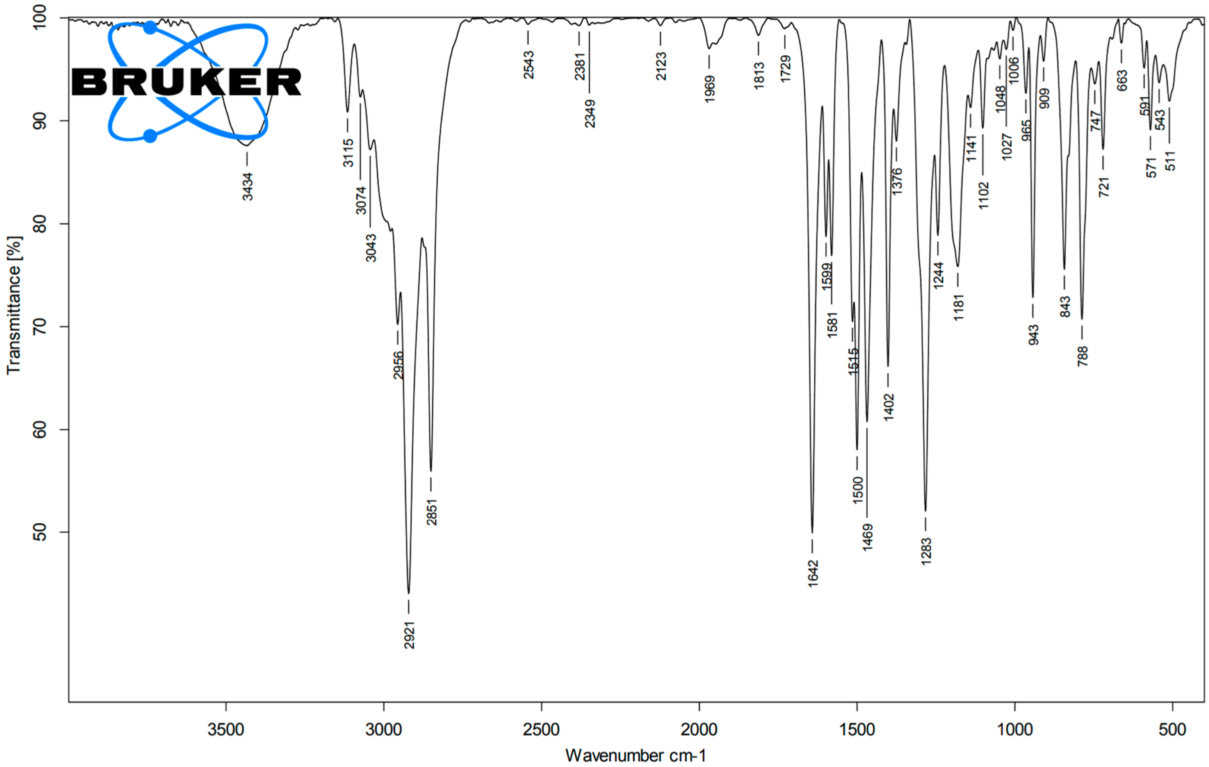

HRMS spectra of bis-QACs.

HRMS spectrum of **5a**

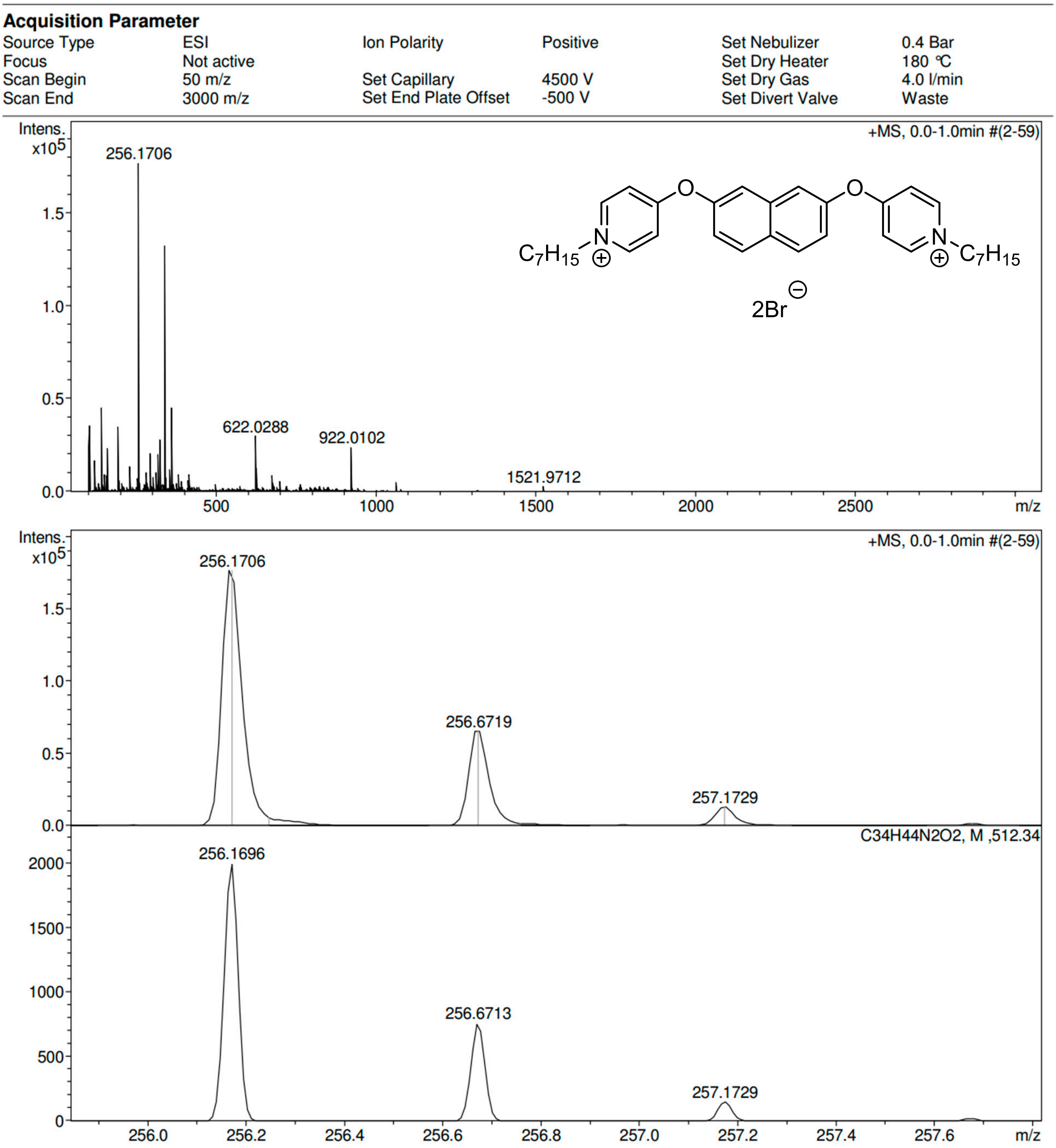

HRMS spectrum of **5b**

Acquisition Parameter

Source Type	ESI	Ion Polarity	Positive			Set Nebulizer	0.4 Bar
Focus	Not active					Set Dry Heater	180 °C
Scan Begin	50 m/z	Set Capillary	4500 V			Set Dry Gas	4.0 l/min
Scan End	3000 m/z	Set End Plate Offset	-500 V			Set Divert Valve	Waste

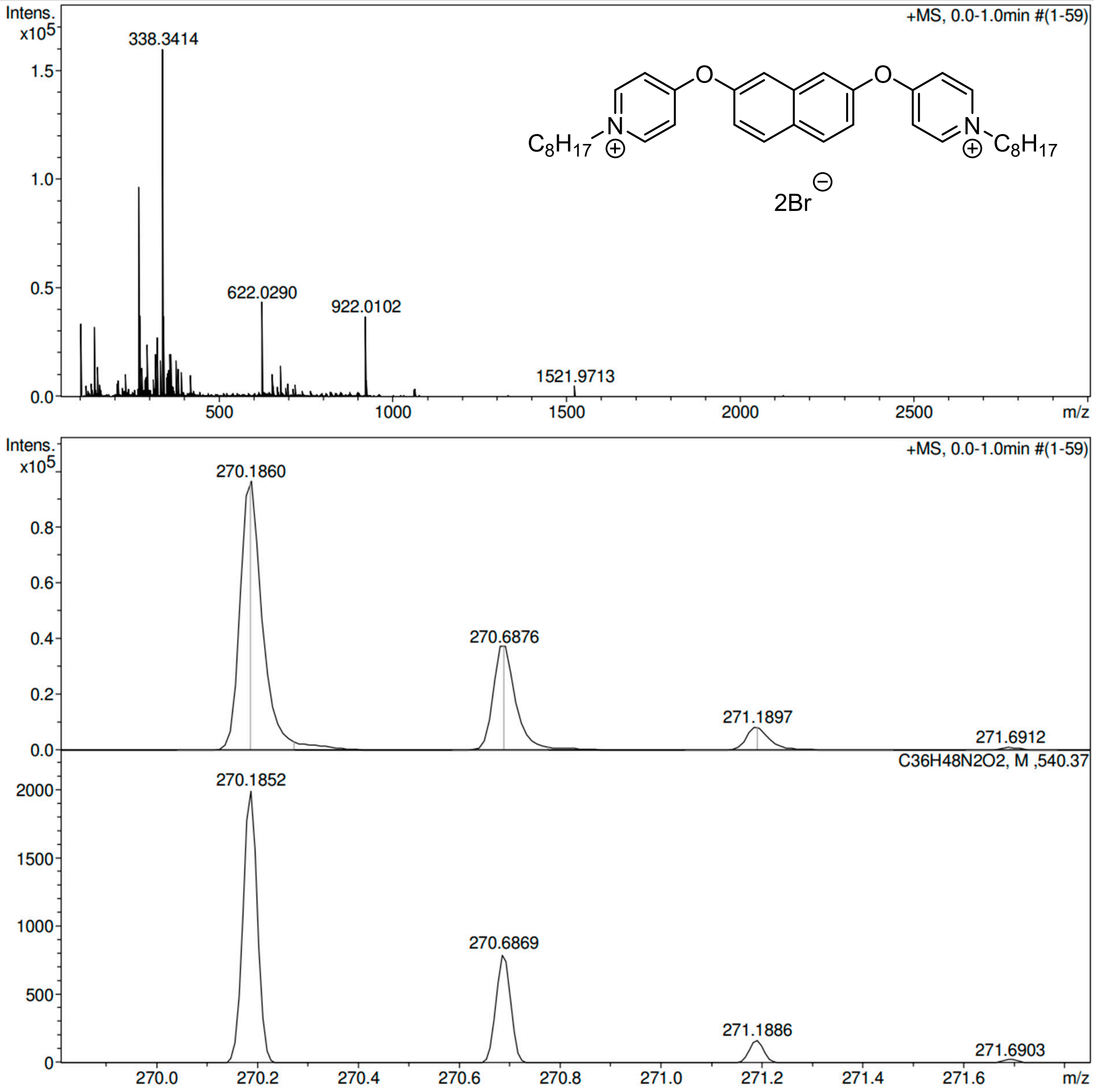

HRMS spectrum of **5d**

Acquisition Parameter

Source Type	ESI	Ion Polarity	Positive	Set Nebulizer	0.4 Bar
Focus	Not active			Set Dry Heater	180 °C
Scan Begin	50 m/z	Set Capillary	4500 V	Set Dry Gas	4.0 l/min
Scan End	3000 m/z	Set End Plate Offset	-500 V	Set Divert Valve	Waste

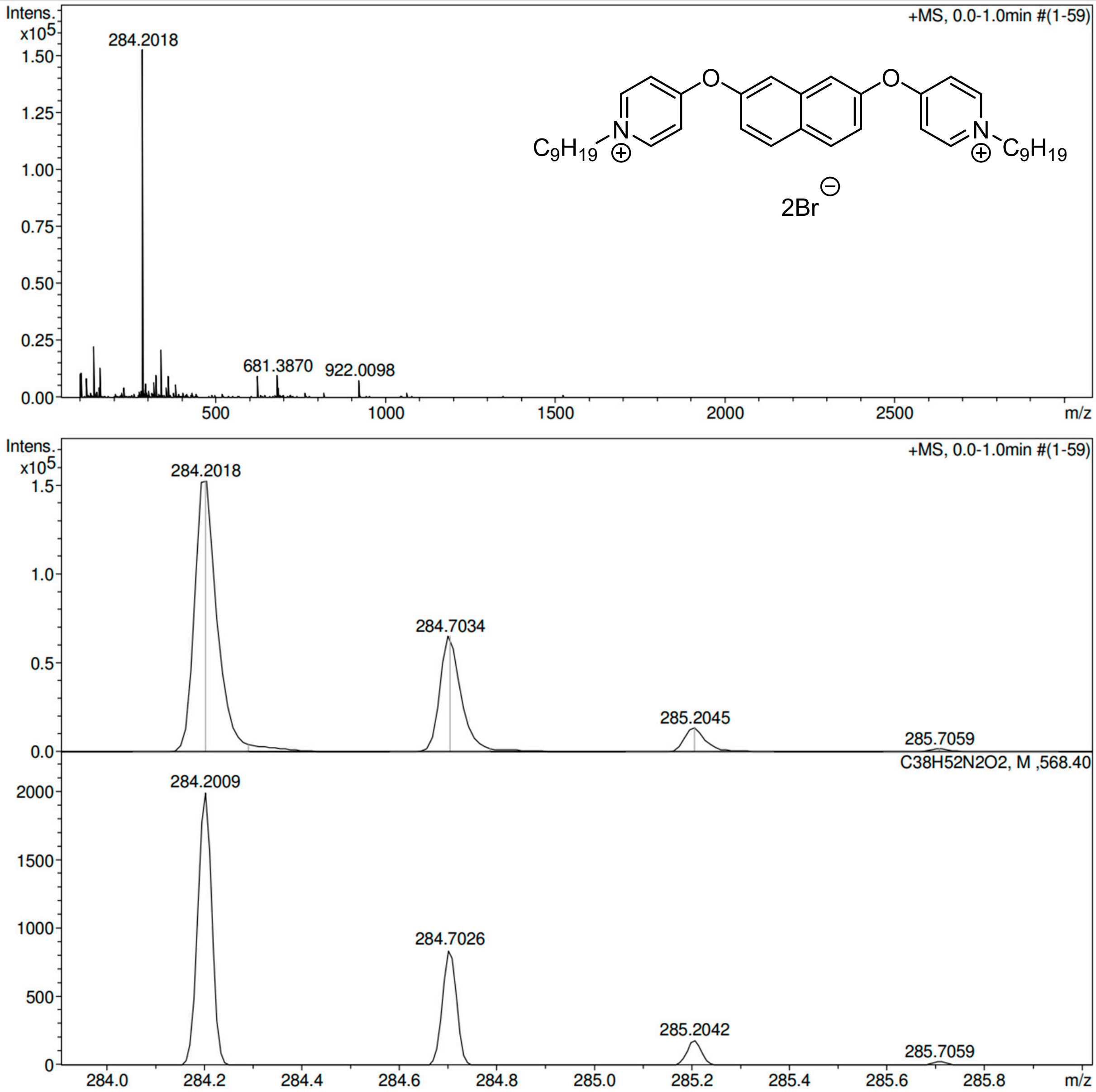

HRMS spectrum of **5e**

Acquisition Parameter

Source Type	ESI	Ion Polarity	Positive	Set Nebulizer	0.4 Bar
Focus	Not active			Set Dry Heater	180 °C
Scan Begin	50 m/z	Set Capillary	4500 V	Set Dry Gas	4.0 l/min
Scan End	3000 m/z	Set End Plate Offset	-500 V	Set Divert Valve	Waste

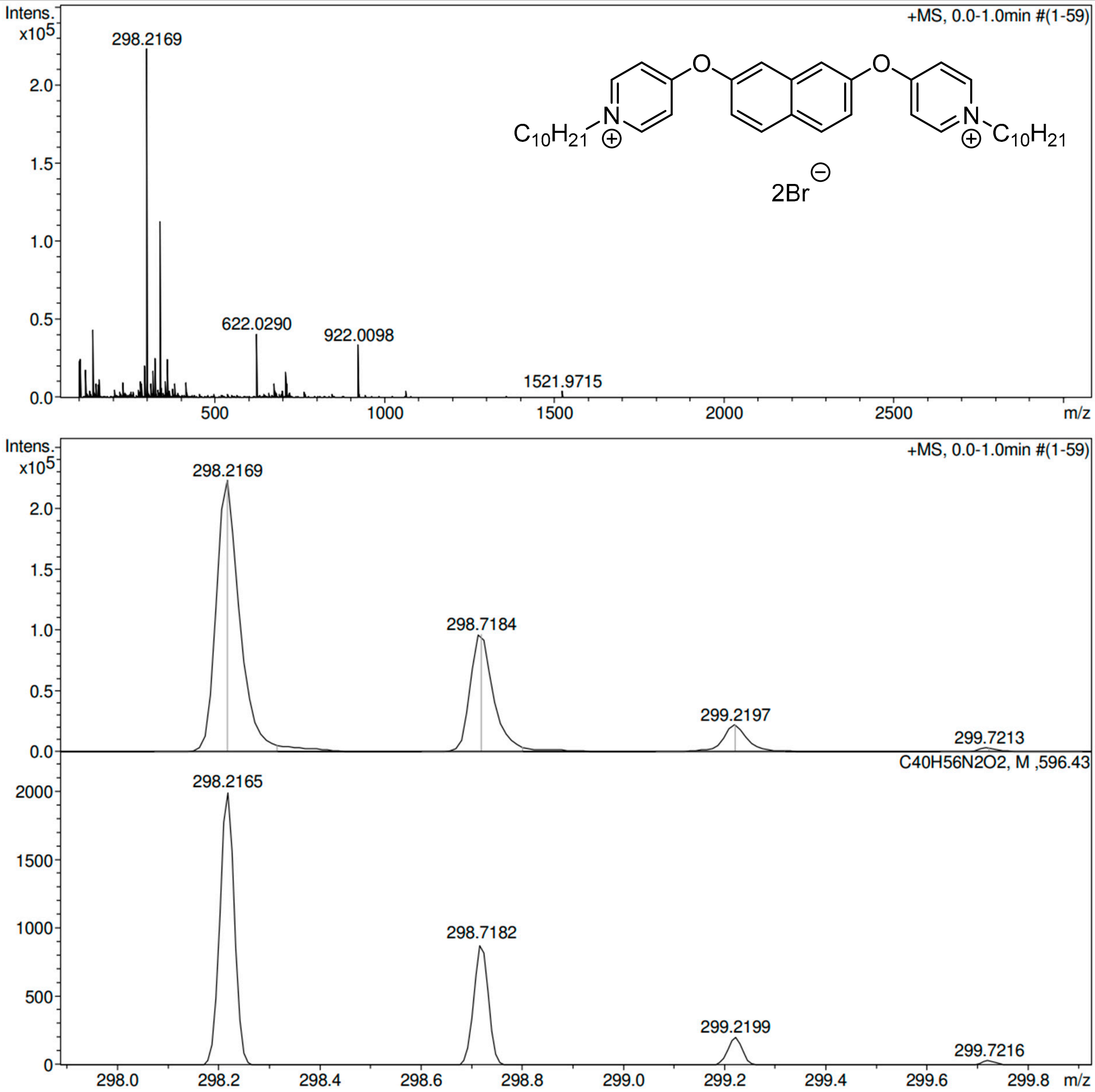

HRMS spectrum of **6a**

Acquisition Parameter

Source Type	ESI	Ion Polarity	Positive	Set Nebulizer	0.4 Bar
Focus	Not active			Set Dry Heater	180 °C
Scan Begin	50 m/z	Set Capillary	4500 V	Set Dry Gas	4.0 l/min
Scan End	3000 m/z	Set End Plate Offset	-500 V	Set Divert Valve	Waste

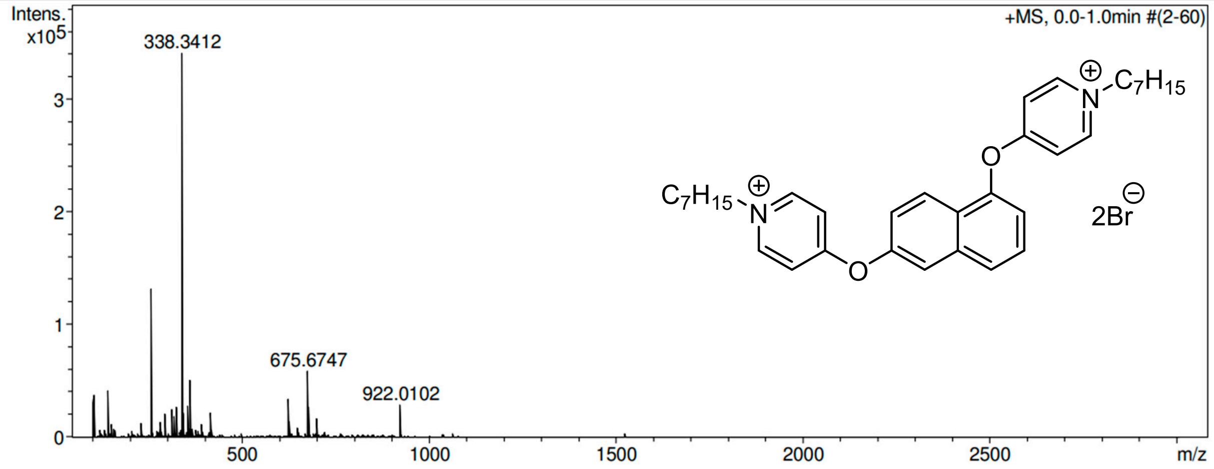

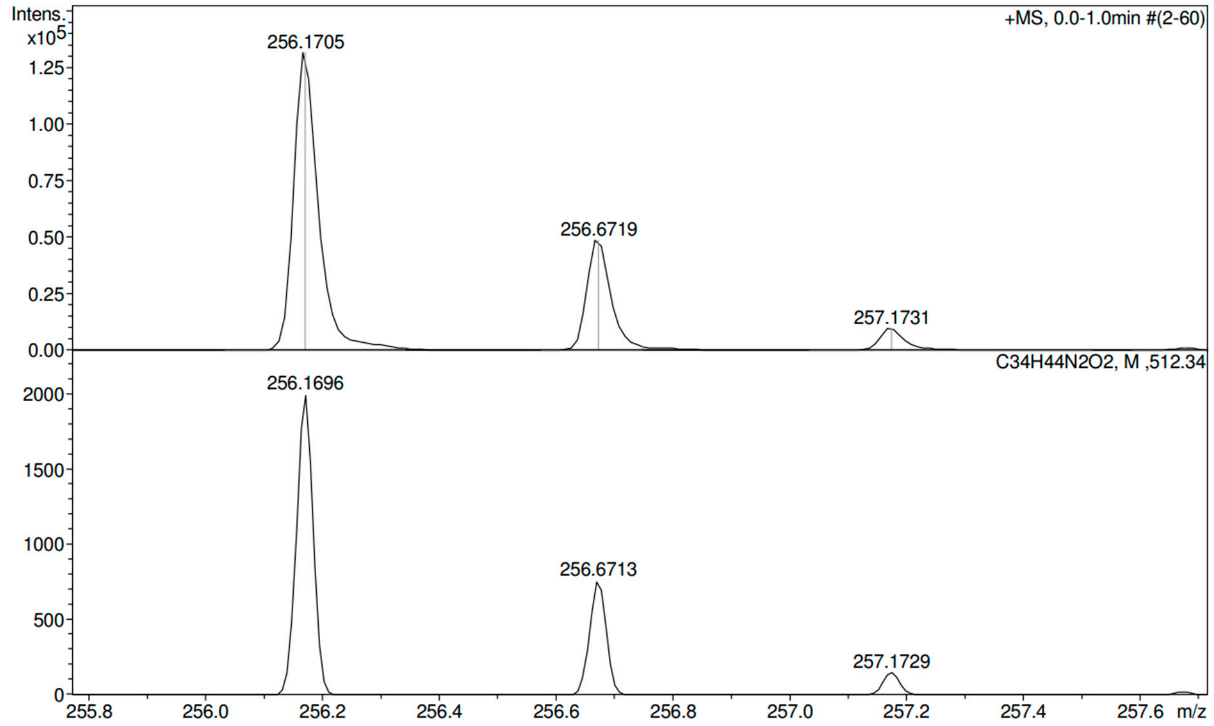

HRMS spectrum of **6b**

Acquisition Parameter

Source Type	ESI	Ion Polarity	Positive	Set Nebulizer	0.4 Bar
Focus	Not active			Set Dry Heater	180 °C
Scan Begin	50 m/z	Set Capillary	4500 V	Set Dry Gas	4.0 l/min
Scan End	3000 m/z	Set End Plate Offset	-500 V	Set Divert Valve	Waste

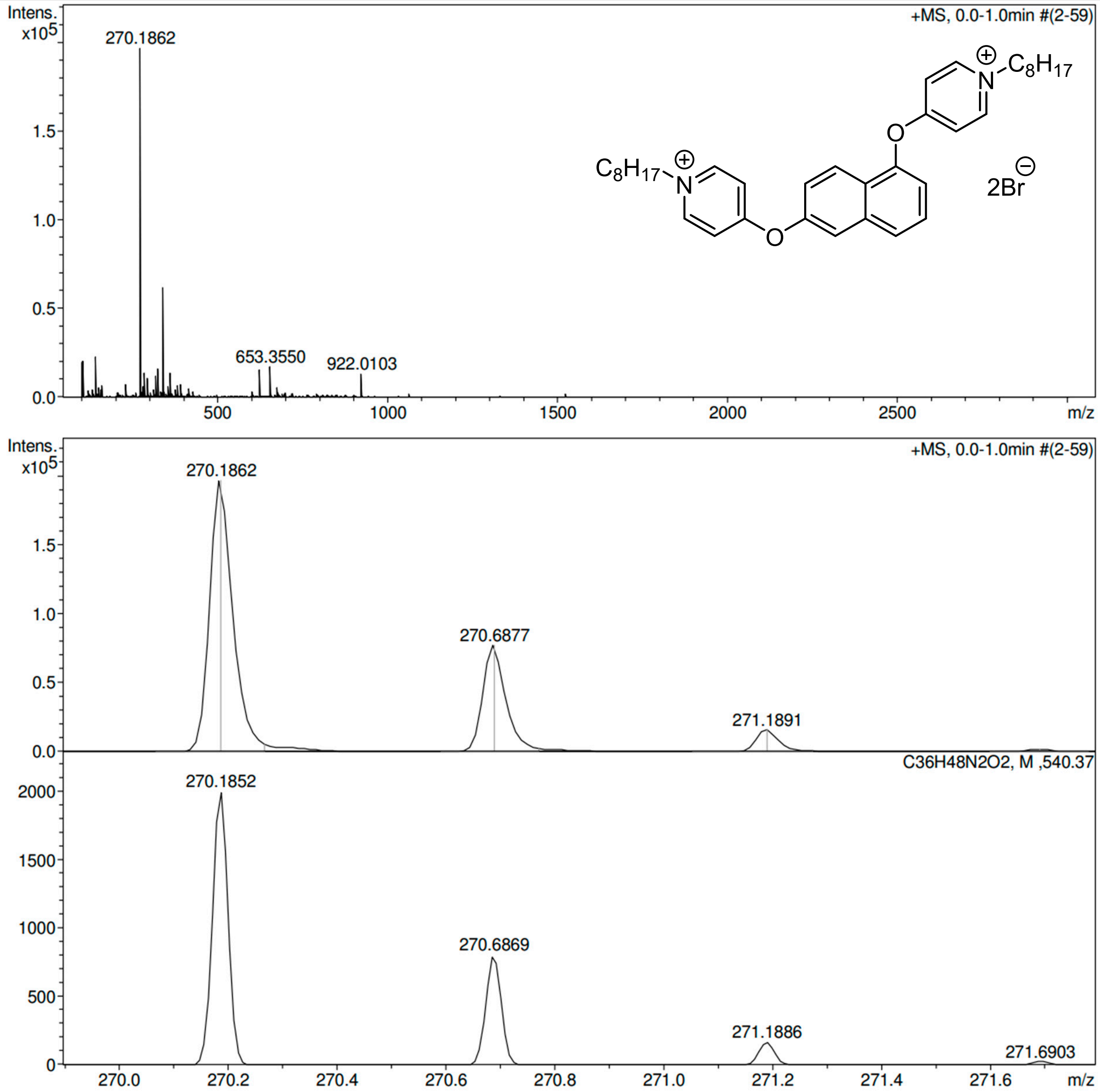

HRMS spectrum of **6d**

Acquisition Parameter

Source Type	ESI	Ion Polarity	Positive	Set Nebulizer	0.4 Bar
Focus	Not active			Set Dry Heater	180 °C
Scan Begin	50 m/z	Set Capillary	4500 V	Set Dry Gas	4.0 l/min
Scan End	3000 m/z	Set End Plate Offset	-500 V	Set Divert Valve	Waste

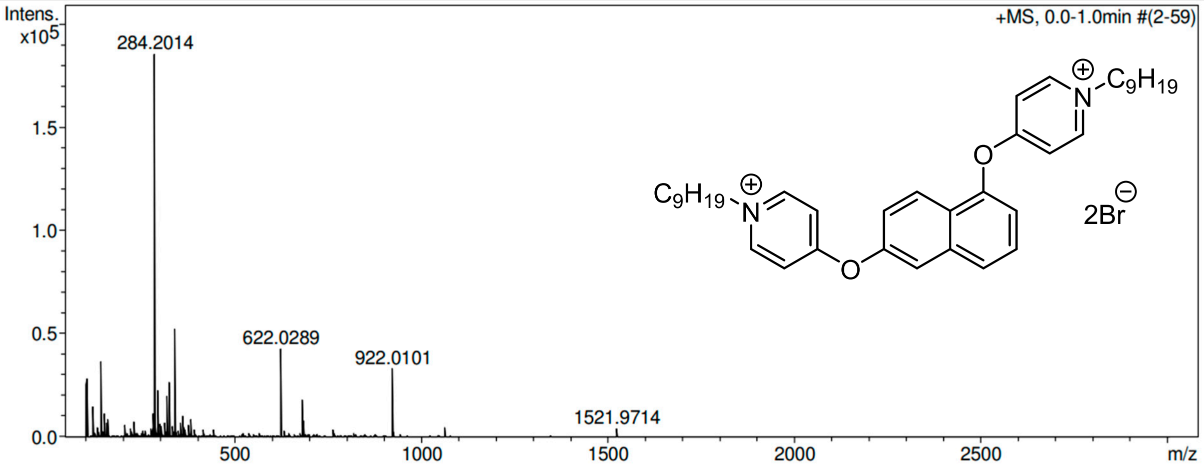

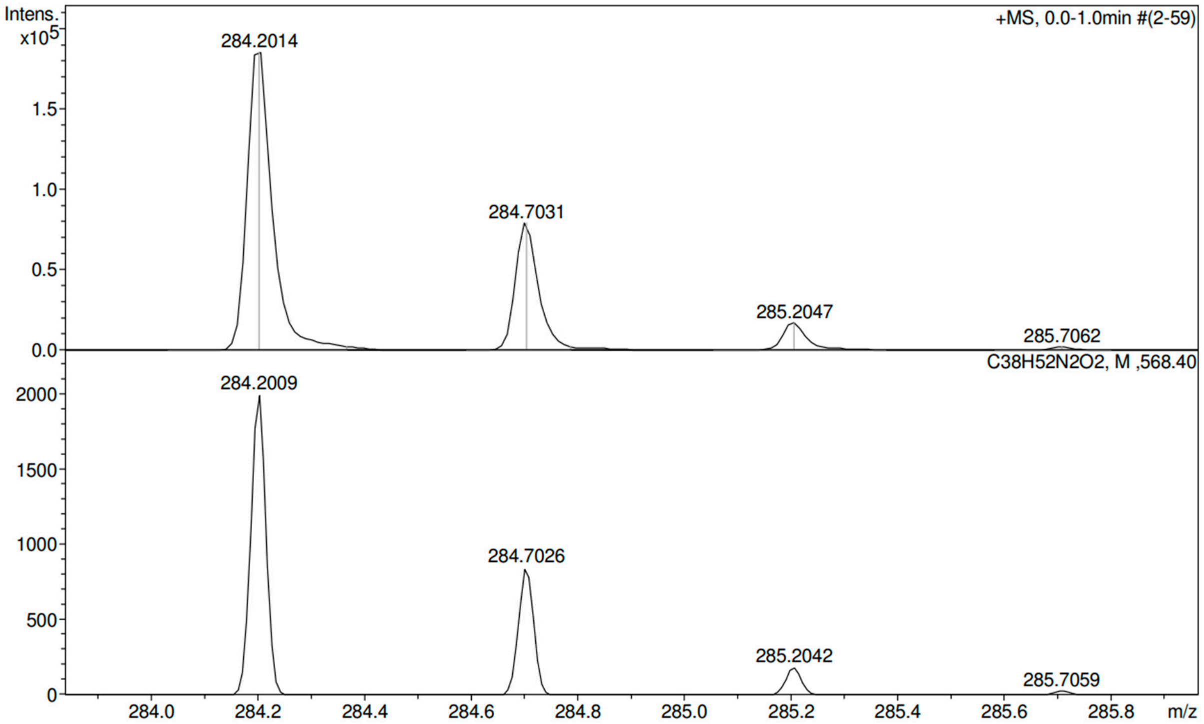

HRMS spectrum of **6e**

Acquisition Parameter

Source Type	ESI	Ion Polarity	Positive	Set Nebulizer	0.4 Bar
Focus	Not active			Set Dry Heater	180 °C
Scan Begin	50 m/z	Set Capillary	4500 V	Set Dry Gas	4.0 l/min
Scan End	3000 m/z	Set End Plate Offset	-500 V	Set Divert Valve	Waste

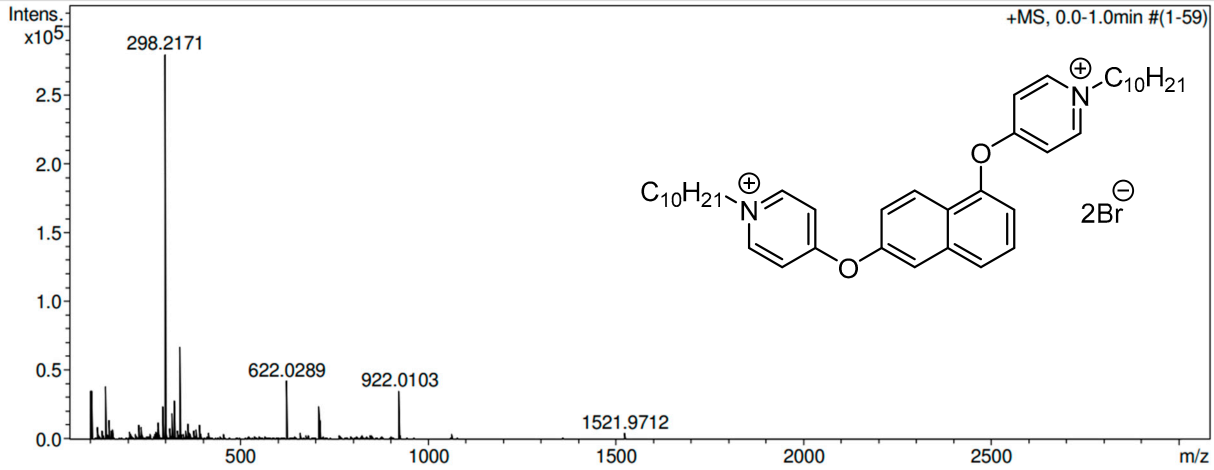

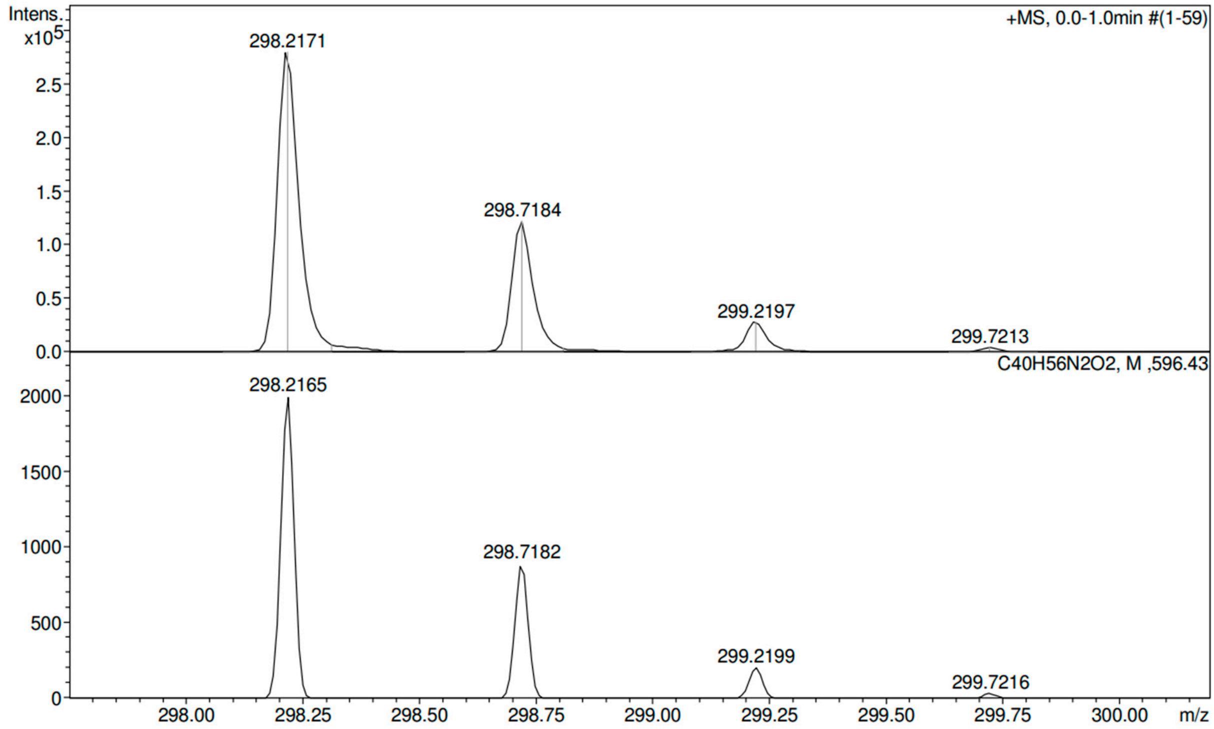

HRMS spectrum of **6f**

Acquisition Parameter

Source Type	ESI	Ion Polarity	Positive	Set Nebulizer	0.4 Bar
Focus	Not active			Set Dry Heater	180 °C
Scan Begin	50 m/z	Set Capillary	4500 V	Set Dry Gas	4.0 l/min
Scan End	3000 m/z	Set End Plate Offset	-500 V	Set Divert Valve	Waste

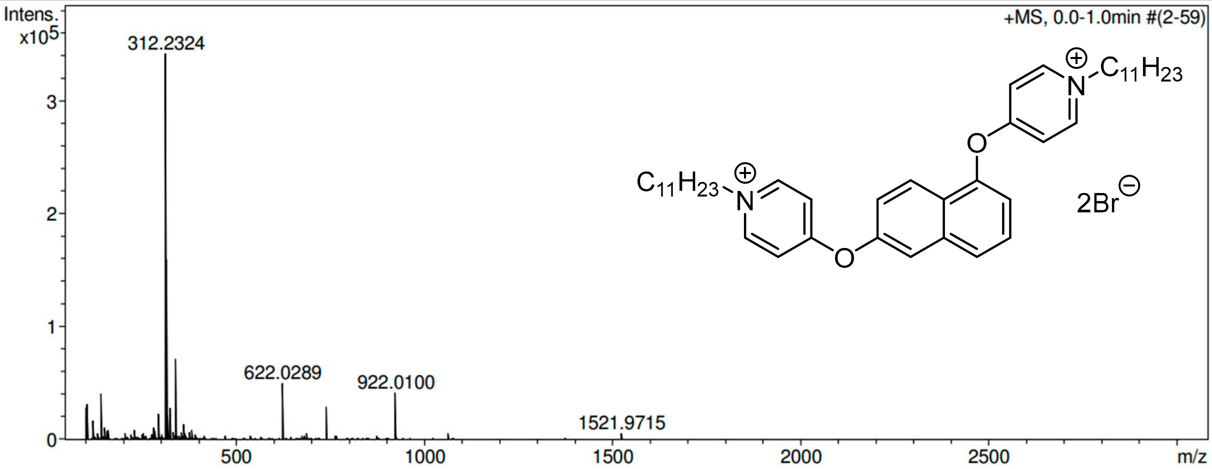

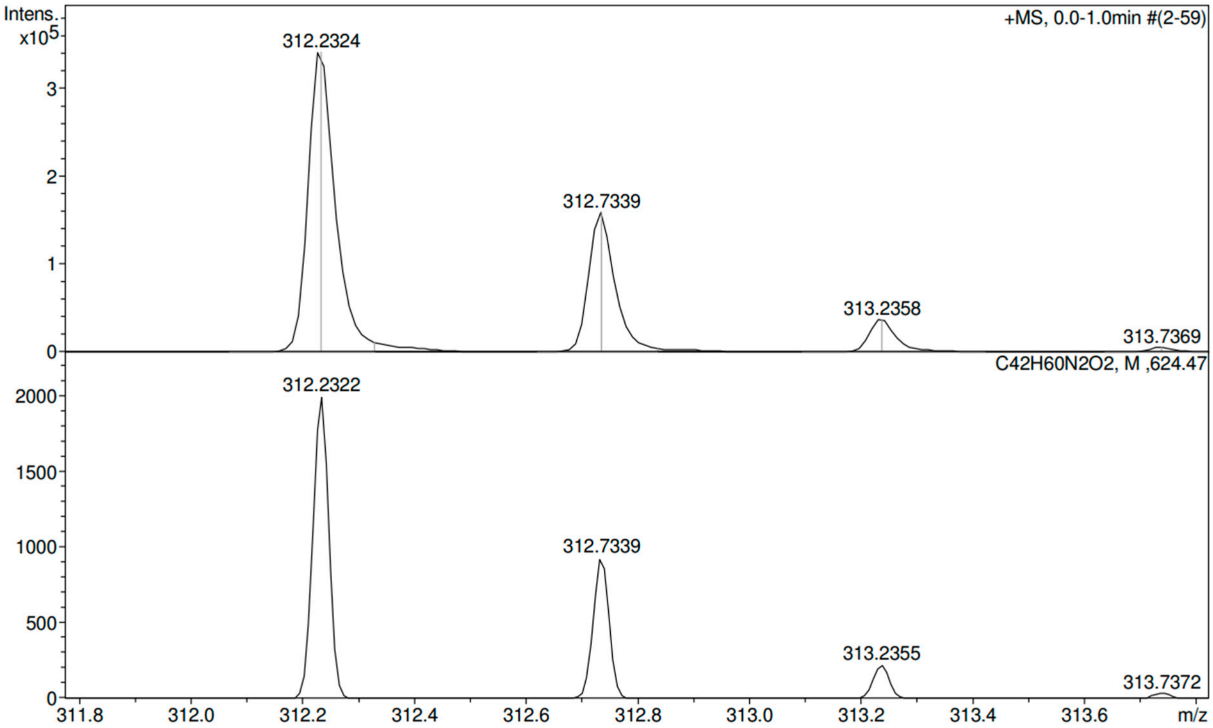

HRMS spectrum of **6g**

Acquisition Parameter

Source Type	ESI	Ion Polarity	Positive	Set Nebulizer	0.4 Bar
Focus	Not active			Set Dry Heater	180 °C
Scan Begin	50 m/z	Set Capillary	4500 V	Set Dry Gas	4.0 l/min
Scan End	3000 m/z	Set End Plate Offset	-500 V	Set Divert Valve	Waste

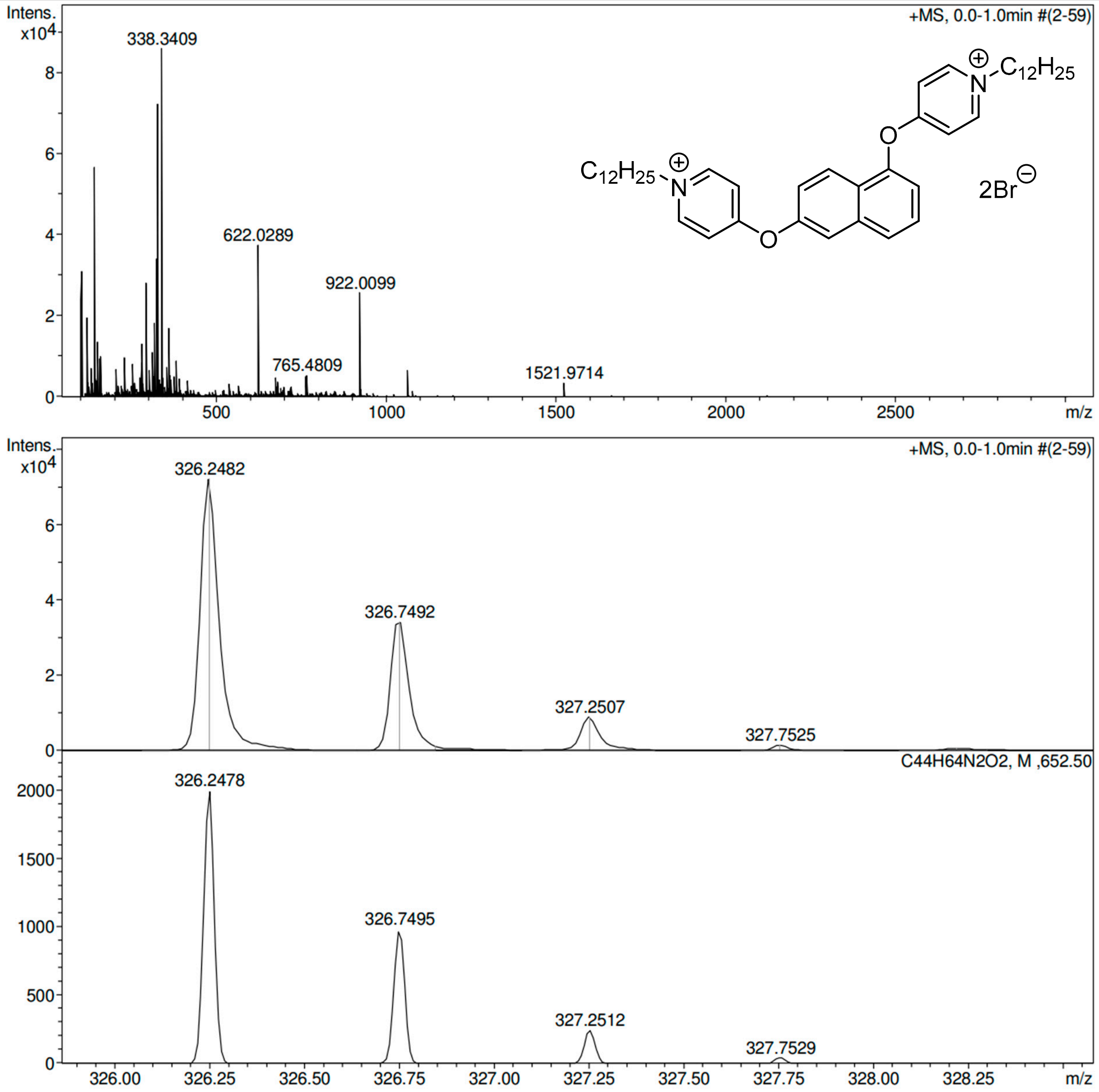

HRMS spectrum of **7a**

Acquisition Parameter

Source Type	ESI	Ion Polarity	Positive			Set Nebulizer	0.4 Bar
Focus	Not active					Set Dry Heater	180 °C
Scan Begin	50 m/z	Set Capillary	4500 V			Set Dry Gas	4.0 l/min
Scan End	3000 m/z	Set End Plate Offset	-500 V			Set Divert Valve	Waste

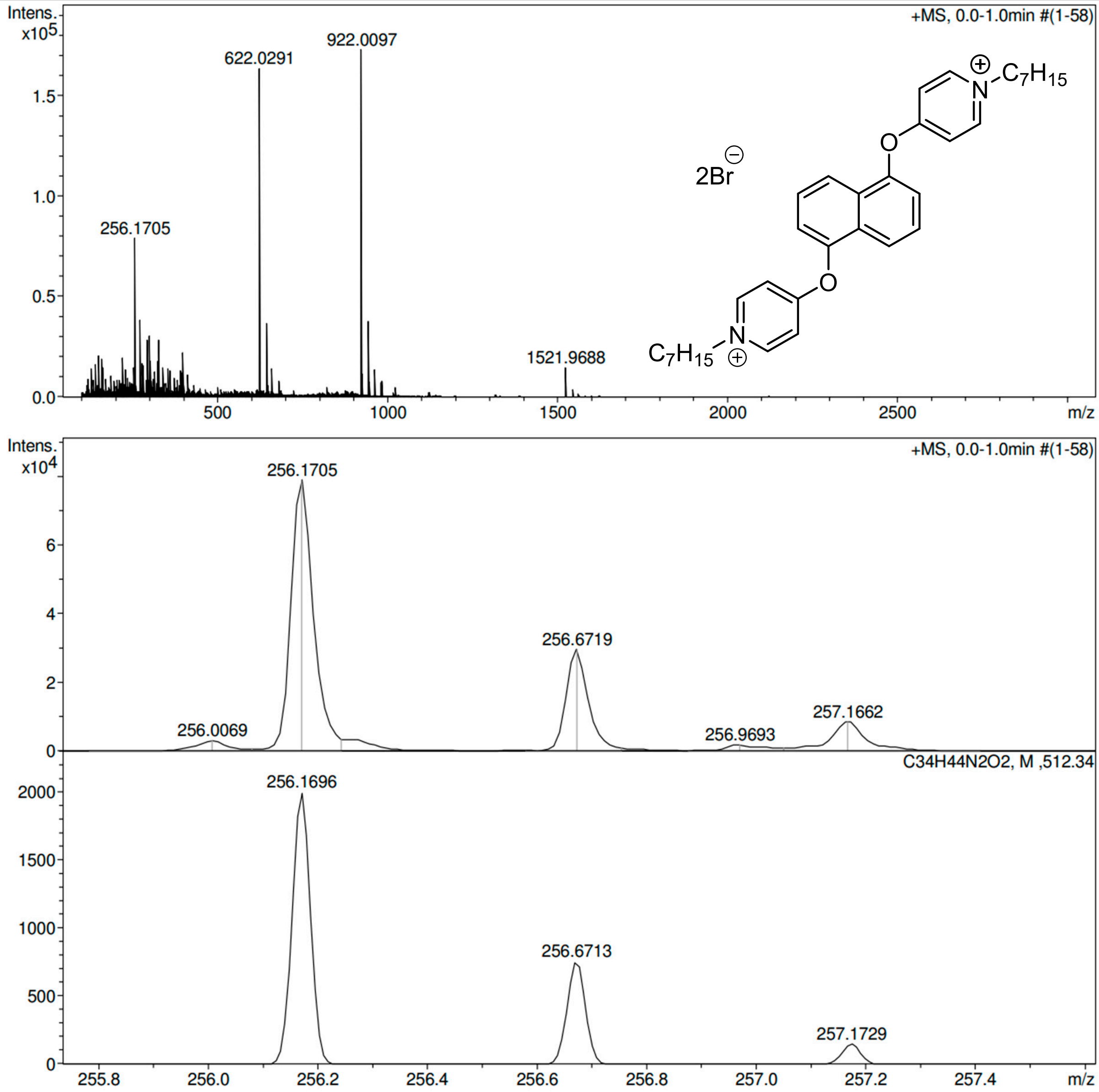

HRMS spectrum of **7b**

Acquisition Parameter

Source Type	ESI	Ion Polarity	Positive	Set Nebulizer	0.4 Bar
Focus	Not active			Set Dry Heater	180 °C
Scan Begin	50 m/z	Set Capillary	4500 V	Set Dry Gas	4.0 l/min
Scan End	3000 m/z	Set End Plate Offset	-500 V	Set Divert Valve	Waste

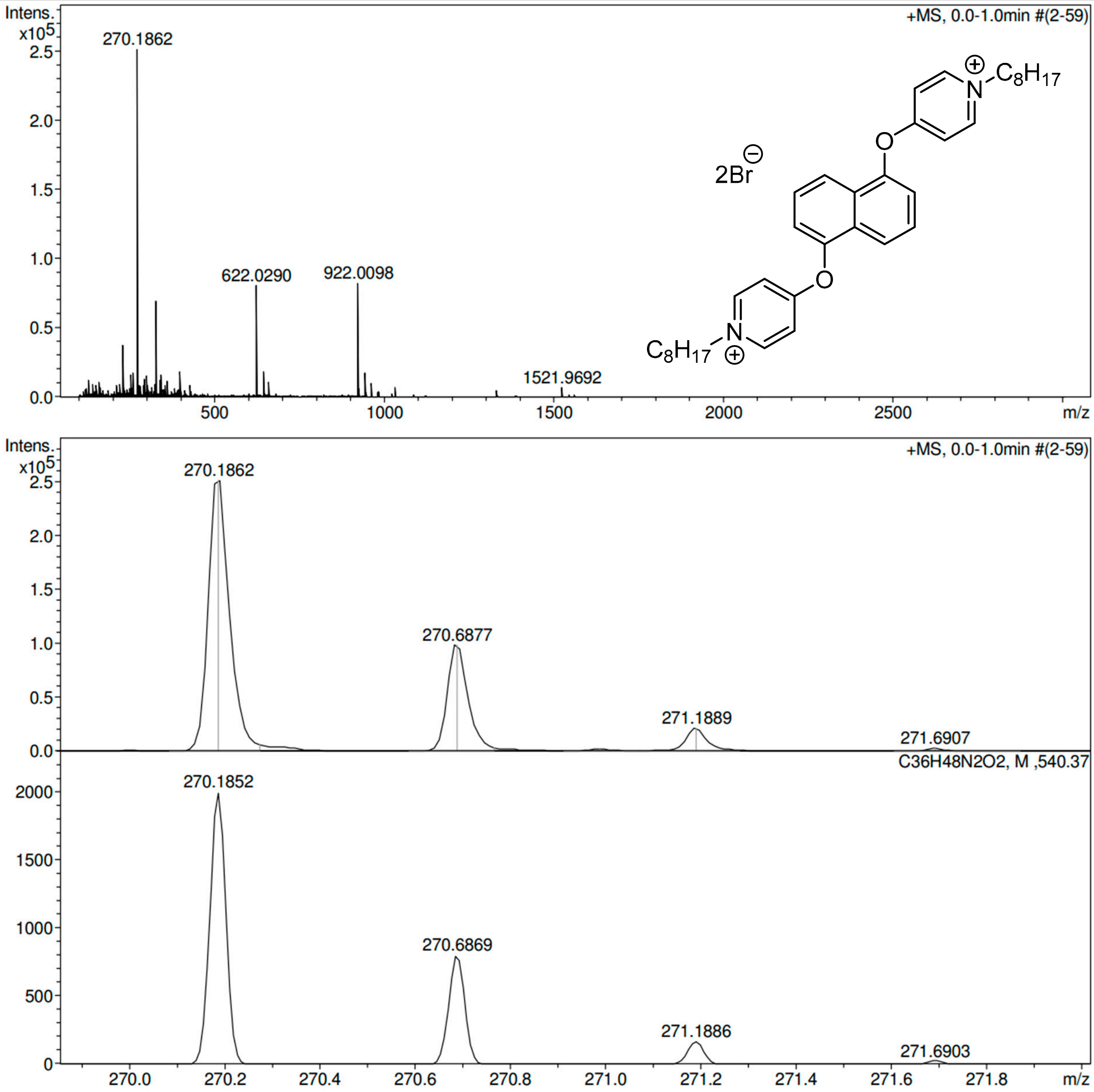

HRMS spectrum of **7c**

Acquisition Parameter

Source Type	ESI	Ion Polarity	Positive	Set Nebulizer	0.4 Bar
Focus	Not active			Set Dry Heater	180 °C
Scan Begin	50 m/z	Set Capillary	4500 V	Set Dry Gas	4.0 l/min
Scan End	3000 m/z	Set End Plate Offset	-500 V	Set Divert Valve	Waste

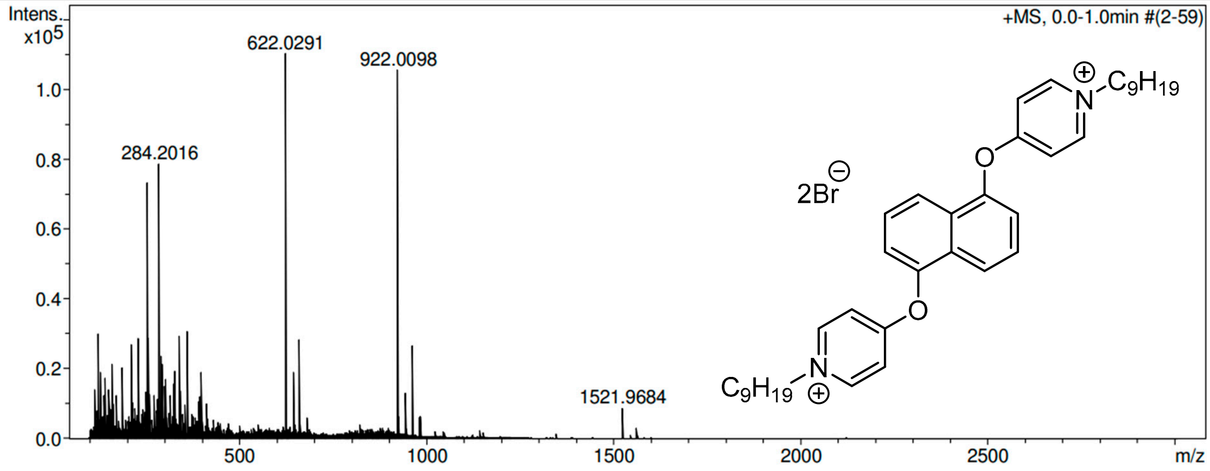

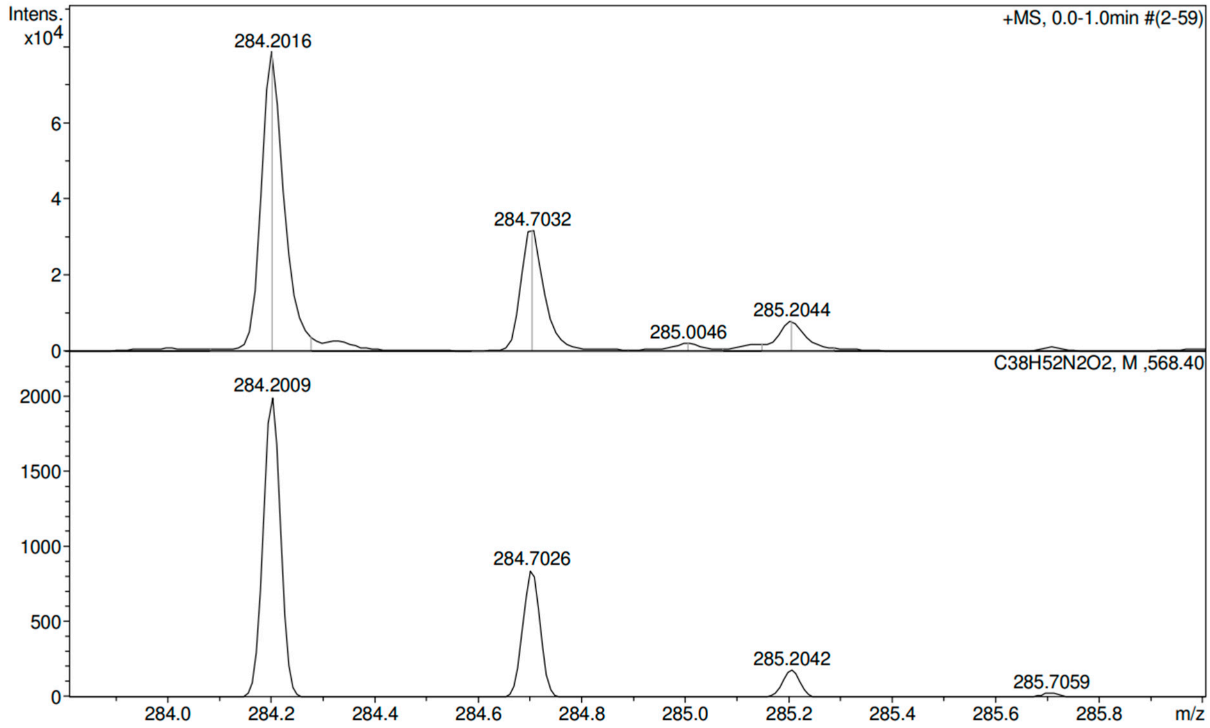

HRMS spectrum of **7f**

Acquisition Parameter

Source Type	ESI	Ion Polarity	Positive	Set Nebulizer	0.4 Bar
Focus	Not active			Set Dry Heater	180 °C
Scan Begin	50 m/z	Set Capillary	4500 V	Set Dry Gas	4.0 l/min
Scan End	3000 m/z	Set End Plate Offset	-500 V	Set Divert Valve	Waste

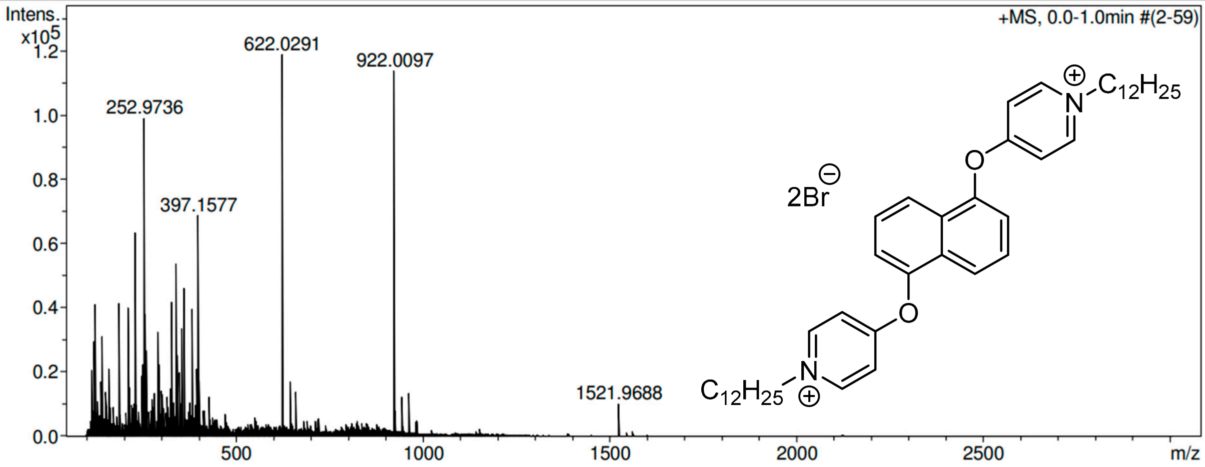

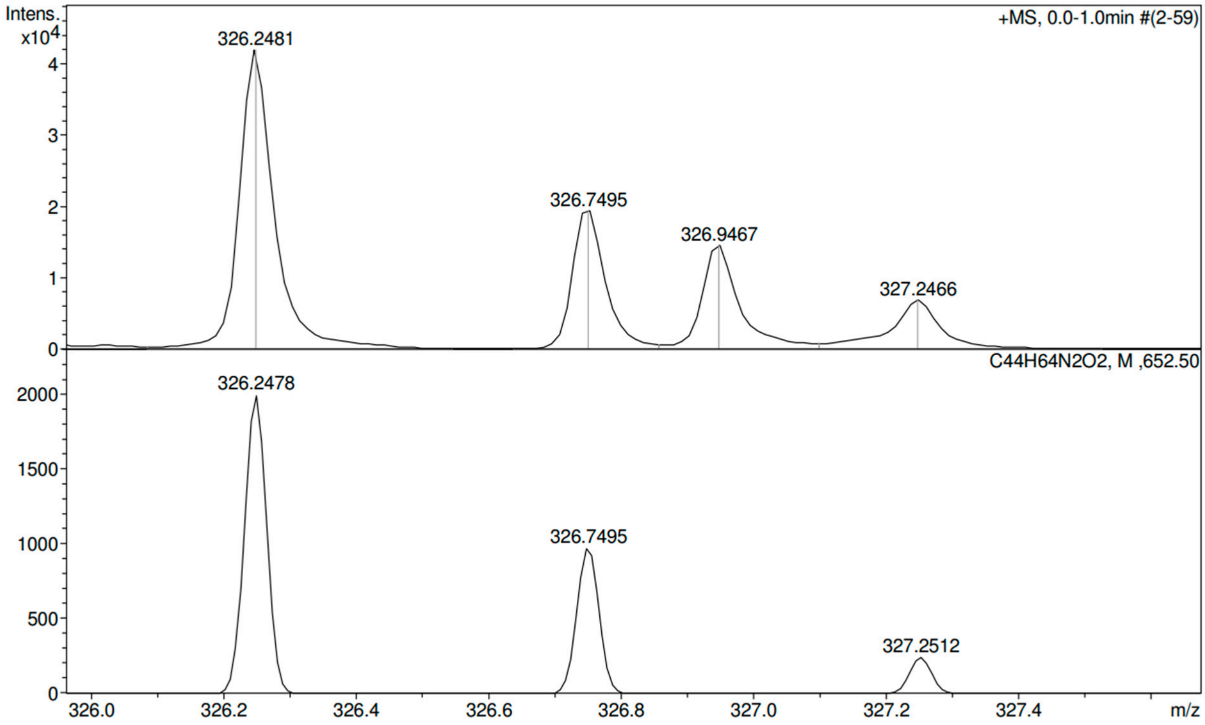

Figures and Tables

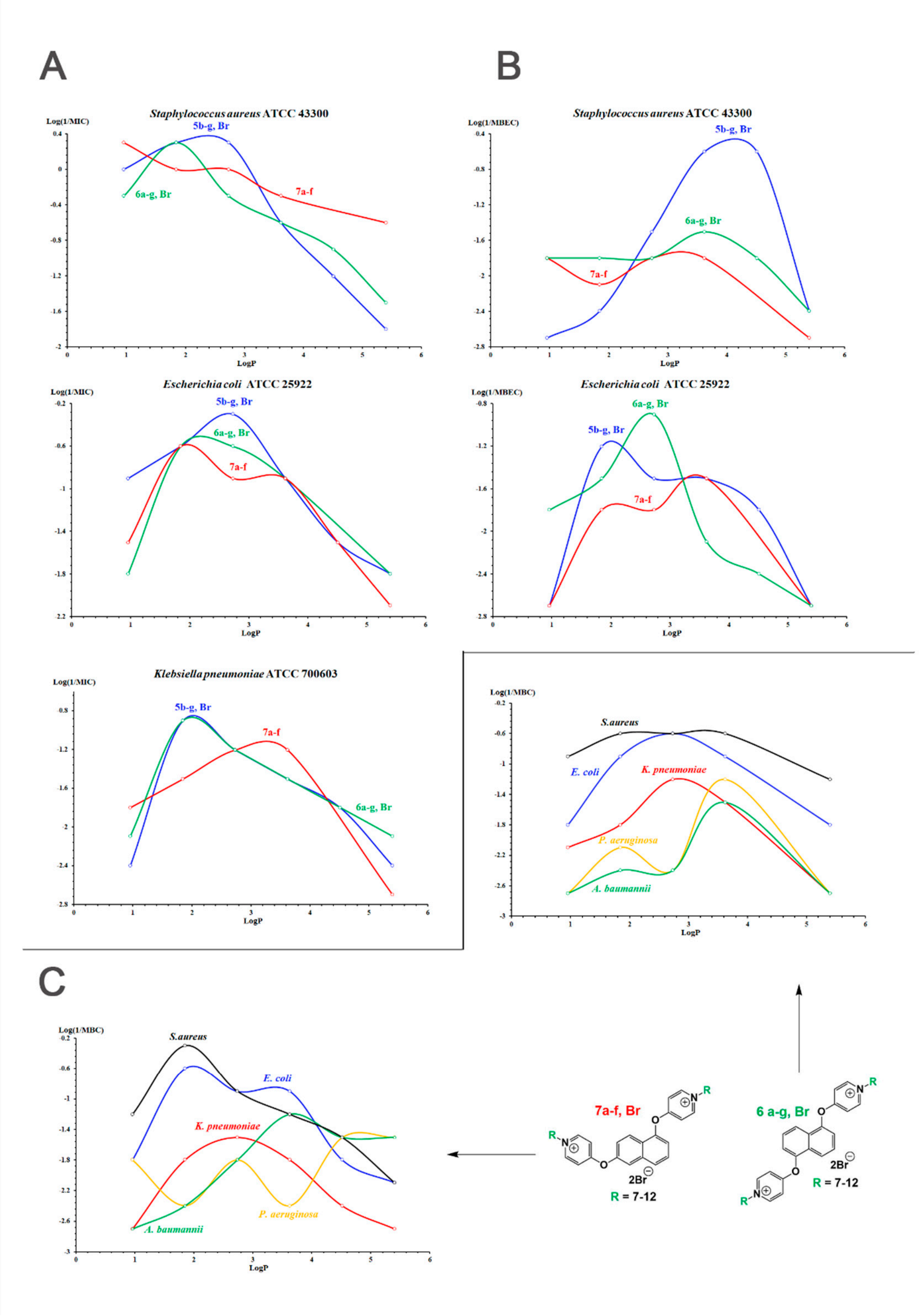

Figure S1. Lipophilicity-activity relationship of QACs. **(A)** The relationship between the calculated LogP and the bactericidal activity (LogMIC^{-1}) of bis-QACs **5-7** against planktonic gram-positive *S. aureus*, *E. coli* and *K. pneumoniae*; (B) The relationship between the calculated LogP and the biofilm inhibition activity (LogMBEC^{-1}) of bis-QACs **5-7** against gram-positive *S. aureus*, *E. coli* biofilms; (C) The relationship between the calculated LogP and the bactericidal activity (LogMBC^{-1}) of bis-QACs **6a-g, 7a-f** against all reference strains.

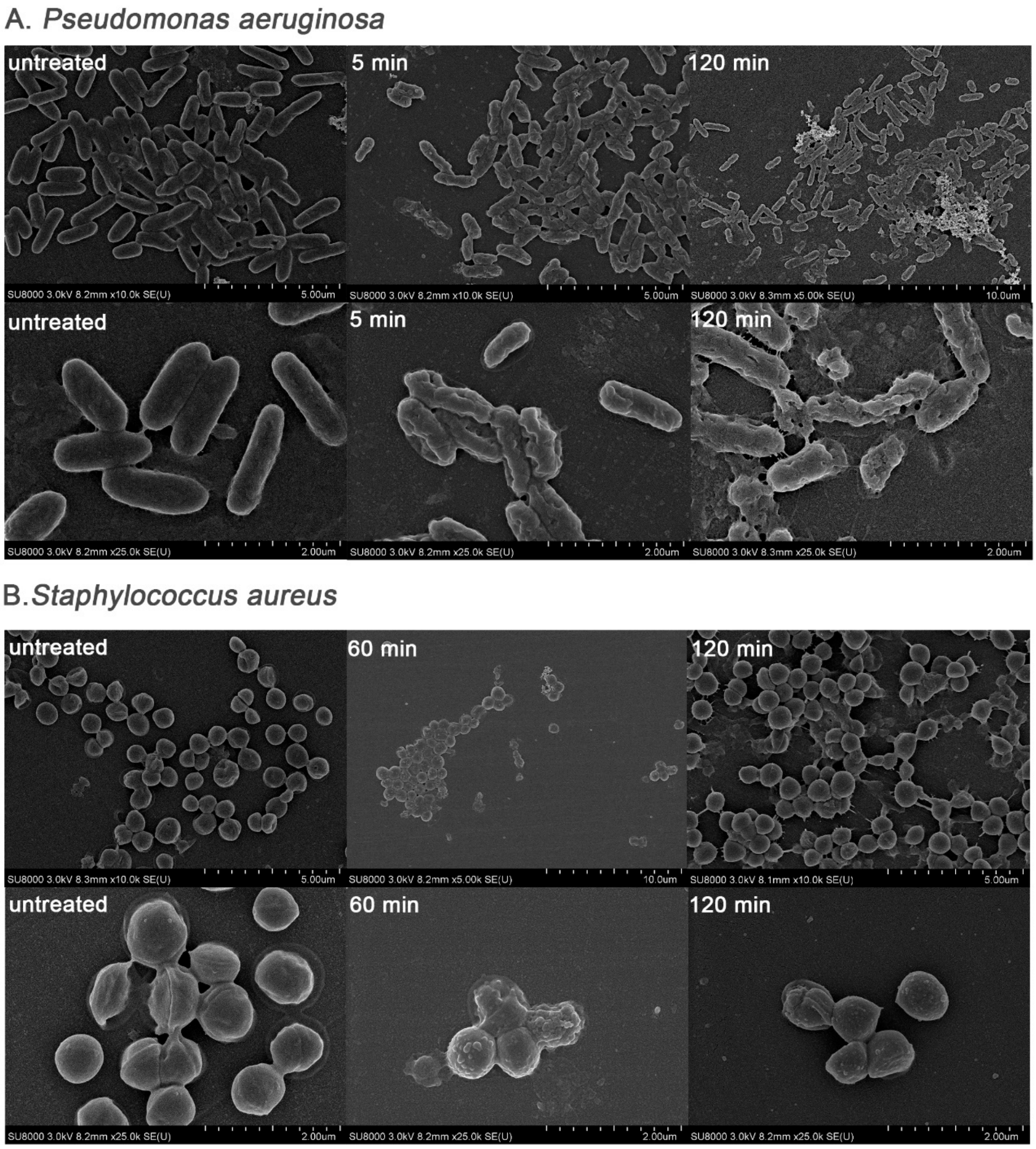

Figure S2. SEM images of *P. aeruginosa* ATCC 27853 (**A**) untreated with QAC, after 5 min and 120 min of treatment with **5d** and S. *aureus* ATCC 43300 (**B**) untreated with QAC, after 60 min and 120 min of treatment with **5d.**

Table S1. Antibacterial activity of bis-QACs **5-7** compared to commercial QACs.

Compounds	Reference strains					Clinical isolates				
	Sa	*Ec*	*Kp*	*Ab*	*Pa*	*Sa*	*Ec*	*Kp*	*Ab*	*Pa*
	Planktonic cells (MIC/MBC [mg/L])									
**5a**: 2,7-, n = 7, Br	1	8	250	>500	250	2	32	63	250	16
	8	32	500	>500	500	4	63	500	250	16
**5b**: 2,7-, n = 8, Br	0.5	4	8	63	32	0.5	4	16	125	63
	4	4	16	63	125	4	4	32	250	125
**5c**: 2,7-, n = 8, I	0.5	4	16	125	63	1	4	16	32	63
	4	4	16	125	250	4	4	32	63	63
**5d**: 2,7-, n = 9, Br	0.5	2	16	32	32	0.5	8	8	32	63
	4	2	32	63	63	4	8	16	63	63
**5e**: 2,7-, n = 10, Br	4	8	32	16	63	4	32	32	32	63
	16	8	32	32	250	16	125	63	32	125
**5f**: 2,7-, n = 11, Br	16	32	63	16	250	16	63	63	32	125
	32	32	250	16	500	63	>500	125	32	250
**5g**: 2,7-, n = 12, Br	63	32	250	16	500	32	125	500	125	>500
	125	63	500	32	>500	500	>500	500	>500	>500
**6a**: 1,6-, n = 7, Br	2	32	125	500	63	4	32	32	500	500
	16	63	500	500	63	4	32	500	500	500
**6b**: 1,6-, n = 8, Br	0.5	4	8	250	32	0.5	4	8	250	125
	2	4	63	250	250	4	16	32	500	250
**6c**: 1,6-, n = 8, I	0.5	4	125	125	125	0.5	8	32	63	125
	4	8	125	125	250	2	16	125	125	250
**6d**: 1,6-, n = 9, Br	2	8	16	63	32	2	8	32	125	63
	8	8	32	63	63	4	16	63	125	63
**6e**: 1,6-, n = 10, Br	4	8	32	8	125	4	8	32	32	63
	16	8	63	16	250	16	125	32	32	125
**6f**: 1,6-, n = 11, Br	8	32	63	16	16	8	63	63	63	63
	32	63	250	32	32	16	63	250	63	125
**6g**: 1,6-, n = 12, Br	32	125	125	16	32	16	63	125	63	250
	125	125	250	32	32	63	125	500	63	250
**7a**: 1,5-, n = 7, Br	0.5	63	63	250	500	4	63	125	250	500
	8	63	125	500	500	16	125	250	500	500
**7b**: 1,5-, n = 8, Br	1	4	32	125	63	1	8	32	63	125
	4	8	63	250	125	4	8	63	125	125
**7c**: 1,5-, n = 9, Br	1	4	16	63	63	2	8	63	63	63
	4	4	16	250	250	16	8	125	63	63
**7d**: 1,5-, n = 10, Br	2	8	16	32	8	4	8	63	32	8
	4	8	32	32	16	8	16	63	32	16
**7f**: 1,5-, n = 12, Br	4	63	>500	500	>500	16	125	>500	500	>500
	16	63	>500	500	>500	32	250	>500	500	>500
**BAC**	125	4	500	>500	>500	125	500	500	>500	>500
	250	8	500	>500	>500	500	>500	500	>500	>500
**MIR**	2	8	32	63	63	4	8	16	63	125
	8	8	32	63	125	16	8	32	250	125
**CPC**	4	8	63	32	500	2	8	8	16	32
	16	8	250	125	>500	16	63	16	32	125
**OCT**	0.5	0.5	4	32	8	0.5	2	2	32	32
	2	0.5	8	125	16	2	4	4	125	63

Note: *Ec* – *E. coli* ATCC 25922 (laboratory strain), *E. coli* B-3421/19 (clinical isolate); *Kp* – *K. pneumoniae* ATCC 70060 (laboratory strain), *K. pneumoniae* B-2523/18 (clinical isolate); *Sa* – *S. aureus* ATCC 43300 (laboratory strain), *S. aureus* B-8648 (clinical isolate); *Pa* – *P. aeruginosa* ATCC 27853 (laboratory strain), *P. aeruginosa* B-2099/18 (clinical isolate); *Ab* – *A. baumannii* ATCC 15308 (laboratory strain), *A. baumannii* B-2926/ 18 (clinical isolate MIC – minimum inhibitory concentration; MBC – minimum bactericidal concentration; color indicates the best result in series, color indicates one dilution difference with the best result, color indicates two dilution difference with the best result; red arrow indicates «cut-off» effect.

Table S2. Toxicological assay and selectivity indices of **5a-g** and **6a-g** against clinical isolates, compared to commercial QACs

Compounds	CC_{50}, mg/L (HEK-293T)	SI_{MIC}/SI_{MBC} (Clinical isolates)				
		Sa	*Ec*	*Kp*	*Ab*	*Pa*
**5a**: 2,7-, n = 7, Br	1,58	0,790	0,049	0,025	0,006	0,099
		0,395	0,025	0,003	0,006	0,099
**5b**: 2,7-, n = 8, Br	1,00	2,000	0,250	0,063	0,008	0,016
		0,250	0,250	0,031	0,004	0,008
**5d**: 2,7-, n = 9, Br	1,225	2,450	0,153	0,153	0,038	0,019
		0,306	0,153	0,077	0,019	0,019
**5e**: 2,7-, n = 10, Br	4,23	1,058	0,132	0,132	0,132	0,067
		0,264	0,034	0,067	0,132	0,034
**5f**: 2,7-, n = 11, Br	31,59	1,974	0,501	0,501	0,987	0,253
		0,501	0,063	0,253	0,987	0,126
**6a**: 1,6-, n = 7, Br	2,67	0,668	0,083	0,083	0,005	0,005
		0,668	0,083	0,005	0,005	0,005
**6b**: 1,6-, n = 8, Br	1,76	3,520	0,440	0,220	0,007	0,014
		0,440	0,110	0,055	0,004	0,007
**6c**: 1,6-, n = 8, I	1,85	3,700	0,231	0,058	0,029	0,015
		0,925	0,116	0,015	0,015	0,007
**6d**: 1,6-, n = 9, Br	1,255	0,628	0,157	0,039	0,010	0,020
		0,314	0,078	0,020	0,010	0,020
**6f**: 1,6-, n = 11, Br	15,98	1,998	0,254	0,254	0,254	0,254
		0,999	0,254	0,064	0,254	0,128
**6g**: 1,6-, n = 12, Br	52,49	3,281	0,833	0,420	0,833	0,210
		0,833	0,420	0,105	0,833	0,210
**BAC**	0,563	0,005	0,001	0,001	0,001	0,001
		0,001	0,001	0,001	0,001	0,001
**MIR**	1,296	0,324	0,162	0,081	0,021	0,010
		0,081	0,162	0,041	0,005	0,010
**CPC**	2,414	1,207	0,302	0,302	0,151	0,075
		0,151	0,038	0,151	0,075	0,019
**OCT**	0,372	0,744	0,186	0,186	0,012	0,012
		0,186	0,093	0,093	0,003	0,006

Note: CC_{50} – half-maximal cytotoxicity concentration; HEK-293T – human embryonic kidney cells 293T; *Ec* – *E. coli* ATCC 25922; *Kp* – *K. pneumoniae* ATCC 70060; *Sa* – *S. aureus* ATCC 43300 ; *Pa* – *P. aeruginosa* ATCC 27853 *Ab* – *A. baumannii* ATCC 15308; SI – selectivity index; MIC – minimum inhibitory concentration; MBC – minimum bactericidal concentration; color indicates the best SI in series, color indicates SI close to 1.

Table S3. Toxicological assay and selectivity indices of **5a-g** and **6a-g** against reference biofilms, compared to commercial QACs

Compounds	CC_{50}, mg/L (HEK-293T)	SI_{MBIC}/SI_{MBEC} (Reference biofilms)				
		Sa	*Ec*	*Kp*	*Ab*	*Pa*
**5a**: 2,7-, n = 7, Br	1.58	0.003<br>0.003	0.003<br>0.003	0.003<br>0.003	0.003<br>0.003	0.003<br>0.003
**5b**: 2,7-, n = 8, Br	1.00	0.004<br>0.004	0.125<br>0.063	0.016<br>0.004	0.004<br>0.002	0.004<br>0.002
**5d**: 2,7-, n = 9, Br	1.225	0.306<br>0.038	0.153<br>0.038	0.038<br>0.005	0.010<br>0.002	0.005<br>0.002
**5e**: 2,7-, n = 10, Br	4.23	2.115<br>1.058	0.264<br>0.132	0.132<br>0.008	0.017<br>0.008	0.017<br>0.008
**5f**: 2,7-, n = 11, Br	31.59	15.795<br>7.898	0.987<br>0.501	0.501<br>0.063	0.253<br>0.063	0.063<br>0.063
**6a**: 1,6-, n = 7, Br	2.67	0.668<br>0.042	0.042<br>0.042	0.005<br>0.005	0.005<br>0.005	0.005<br>0.005
**6b**: 1,6-, n = 8, Br	1.76	0.220<br>0.028	0.055<br>0.055	0.028<br>0.004	0.004<br>0.004	0.004<br>0.004
**6c**: 1,6-, n = 8, I	1.85	0.463<br>0.029	0.231<br>0.116	0.029<br>0.007	0.007<br>0.004	0.007<br>0.004
**6d**: 1,6-, n = 9, Br	1.255	0.157<br>0.020	0.157<br>0.157	0.020<br>0.003	0.020<br>0.005	0.003<br>0.003
**6f**: 1,6-, n = 11, Br	15.98	0.999<br>0.254	0.254<br>0.064	0.128<br>0.032	0.254<br>0.032	0.064<br>0.032
**6g**: 1,6-, n = 12, Br	52.49	1.640<br>0.210	0.105<br>0.105	0.105<br>0.105	0.105<br>0.105	0.105<br>0.105
**BAC**	0.563	0.001<br>0.001	0.002<br>0.002	0.001<br>0.001	0.001<br>0.001	0.001<br>0.001
**MIR**	1.296	0.081<br>0.041	0.041<br>0.041	0.010<br>0.003	0.021<br>0.005	0.003<br>0.003
**CPC**	2.414	0.151<br>0.038	0.151<br>0.038	0.038<br>0.010	0.010<br>0.005	0.005<br>0.005
**OCT**	0.372	0.093<br>0.047	0.047<br>0.023	0.023<br>0.006	0.001<br>0.001	0.003<br>0.001

Note: CC_{50} – half-maximal cytotoxicity concentration; HEK-293T – human embryonic kidney cells 293T; *Ec* – *E. coli* ATCC 25922; *Kp* – *K. pneumoniae* ATCC 70060; *Sa* – *S. aureus* ATCC 43300 ; *Pa* – *P. aeruginosa* ATCC 27853 *Ab* – *A. baumannii* ATCC 15308; SI – selectivity index); MBIC - minimum biofilm inhibitory concentration, MBEC - minimum biofilm eradication concentration; color indicates the best SIs in series, color indicates SI close to 1.

Table S4. Toxicological assay and selectivity indices of **5a-g** and **6a-g** against clinical biofilms, compared to commercial QACs

Compounds	CC_{50}, mg/L (HEK-293T)	SI_{MBIC}/SI_{MBEC} (Clinical biofilms)				
		Sa	*Ec*	*Kp*	*Ab*	*Pa*
**5a**: 2,7-, n = 7, Br	1.58	0.003<br>0.003	0.003<br>0.003	0.003<br>0.003	0.003<br>0.003	0.003<br>0.003
**5b**: 2,7-, n = 8, Br	1.00	0.004<br>0.004	0.063<br>0.031	0.016<br>0.002	0.004<br>0.002	0.008<br>0.002
**5d**: 2,7-, n = 9, Br	1.225	0.306<br>0.077	0.077<br>0.019	0.019<br>0.005	0.005<br>0.002	0.005<br>0.002
**5e**: 2,7-, n = 10, Br	4.23	1.058<br>1.058	0.067<br>0.067	0.067<br>0.008	0.008<br>0.008	0.034<br>0.008
**5f**: 2,7-, n = 11, Br	31.59	15.795<br>7.898	0.126<br>0.063	0.253<br>0.063	0.063<br>0.063	0.063<br>0.063
**6a**: 1,6-, n = 7, Br	2.67	0.668<br>0.334	0.083<br>0.083	0.005<br>0.005	0.005<br>0.005	0.005<br>0.005
**6b**: 1,6-, n = 8, Br	1.76	0.440<br>0.055	0.028<br>0.028	0.014<br>0.004	0.004<br>0.004	0.004<br>0.004
**6c**: 1,6-, n = 8, I	1.85	0.231<br>0.029	0.231<br>0.058	0.029<br>0.007	0.007<br>0.004	0.007<br>0.004
**6d**: 1,6-, n = 9, Br	1.255	0.314<br>0.157	0.157<br>0.078	0.020<br>0.010	0.020<br>0.010	0.003<br>0.003
**6f**: 1,6-, n = 11, Br	15.98	1.998<br>0.999	0.128<br>0.064	0.128<br>0.032	0.128<br>0.032	0.032<br>0.032
**6g**: 1,6-, n = 12, Br	52.49	1.640<br>0.420	0.105<br>0.105	0.105<br>0.105	0.105<br>0.105	0.105<br>0.105
**BAC**	0.563	0.001<br>0.001	0.001<br>0.001	0.001<br>0.001	0.001<br>0.001	0.001<br>0.001
**MIR**	1.296	0.081<br>0.041	0.021<br>0.005	0.021<br>0.003	0.021<br>0.005	0.003<br>0.003
**CPC**	2.414	0.302<br>0.075	0.019<br>0.010	0.038<br>0.005	0.010<br>0.005	0.005<br>0.005
**OCT**	0.372	0.093<br>0.047	0.023<br>0.003	0.023<br>0.003	0.012<br>0.001	0.001<br>0.001

Note: CC_{50} – half-maximal cytotoxicity concentration; HEK-293T – human embryonic kidney cells 293T; *Ec – E. coli* ATCC 25922; *Kp – K. pneumoniae* ATCC 70060; *Sa – S. aureus* ATCC 43300 ; *Pa – P. aeruginosa* ATCC 27853 *Ab – A. baumannii* ATCC 15308; SI – selectivity index;); MBIC - minimum biofilm inhibitory concentration, MBEC - minimum biofilm eradication concentration; color indicates the best SIs in series, color indicates SI close to 1.

Table S5. Hemolytic activity and selectivity indices of **5d** and **6d** against clinical isolates, compared to commercial QACs

Compounds	HC_{50}, mg/L (hRBCs)	SI_{MIC}/SI_{MBC} (Clinical isolates)				
		Sa	*Ec*	*Kp*	*Ab*	*Pa*
**5d**: 2,7-, n = 9, Br	36.12	72.240	4.515	4.515	1.129	0.573
		9.030	4.515	2.258	0.573	0.573
**6d**: 1,6-, n = 9, Br	36.45	18.225	4.556	1.139	0.292	0.579
		9.113	2.278	0.579	0.292	0.579
**BAC**	37.33	0.299	0.075	0.075	0.075	0.075
		0.075	0.075	0.075	0.075	0.075
**MIR**	21.78	5.445	2.723	1.361	0.346	0.174
		1.361	2.723	0.681	0.087	0.174
**OCT**	17.97	35.940	8.985	8.985	0.562	0.562
		8.985	4.493	4.493	0.144	0.285

Note: HC_{50} – half-maximal hemolytic concentration; hRBCs – human red blood cells; *Ec* – *E. coli* ATCC 25922; *Kp* – *K. pneumoniae* ATCC 70060; *Sa* – *S. aureus* ATCC 43300; *Pa* – *P. aeruginosa* ATCC 27853 *Ab* – *A. baumannii* ATCC 15308; SI – selectivity index; MIC – minimum inhibitory concentration; MBC – minimum bactericidal concentration; color indicates the best SIs in series higher than 10, color indicates SIs in the 5 to 10 range, color indicates SIs in the 1 to 5 range.

Table S6. Hemolytic activity and selectivity indices of **5d** and **6d** against reference biofilms, compared to commercial QACs

Compounds	HC_{50}, mg/L (hRBCs)	SI_{MBIC}/SI_{MBEC} (Reference biofilms)				
		Sa	*Ec*	*Kp*	*Ab*	*Pa*
**5d**: 2,7-, n = 9, Br	36.12	9.030	4.515	1.129	0.289	0.144
		1.129	1.129	0.144	0.072	0.072
**6d**: 1,6-, n = 9, Br	36.45	4.556	4.556	0.579	0.579	0.073
		0.579	4.556	0.073	0.146	0.073
**BAC**	37.33	0.075	0.149	0.075	0.075	0.075
		0.075	0.149	0.075	0.075	0.075
**MIR**	21.78	1.361	0.681	0.174	0.346	0.044
		0.681	0.681	0.044	0.087	0.044
**OCT**	17.97	4.493	2.246	1.123	0.072	0.144
		2.246	1.123	0.285	0.072	0.036

Note: HC_{50} – half-maximal hemolytic concentration; hRBCs – human red blood cells; *Ec* – *E. coli* ATCC 25922; *Kp* – *K. pneumoniae* ATCC 70060; *Sa* – *S. aureus* ATCC 43300; *Pa* – *P. aeruginosa* ATCC 27853 *Ab* – *A. baumannii* ATCC 15308; SI – selectivity index;); MBIC - minimum biofilm inhibitory concentration, MBEC - minimum biofilm eradication concentration; color indicates the best SIs in series higher than 10, color indicates SIs in the 5 to 10 range, color indicates SIs in the 1 to 5 range.

Table S7. Hemolytic activity and selectivity indices of **5d** and **6d** against clinical biofilms, compared to commercial QACs

Compounds	HC_{50}, mg/L (hRBCs)	SI_{MBIC}/SI_{MBEC} (Clinical biofilms)				
		Sa	*Ec*	*Kp*	*Ab*	*Pa*
**5d**: 2,7-, n = 9, Br	36.12	9.030	2.258	0.573	0.144	0.144
		2.258	0.573	0.144	0.072	0.072
**6d**: 1,6-, n = 9, Br	36.45	9.113	4.556	0.579	0.579	0.073
		4.556	2.278	0.292	0.292	0.073
**BAC**	37.33	0.075	0.075	0.075	0.075	0.075
		0.075	0.075	0.075	0.075	0.075
**MIR**	21.78	1.361	0.346	0.346	0.346	0.044
		0.681	0.087	0.044	0.087	0.044
**OCT**	17.97	4.493	1.123	1.123	0.562	0.072
		2.246	0.144	0.144	0.072	0.072

Note: HC_{50} – half-maximal hemolytic concentration; hRBCs – human red blood cells; *Ec – E. coli* ATCC 25922; *Kp – K. pneumoniae* ATCC 70060; *Sa – S. aureus* ATCC 43300; *Pa – P. aeruginosa* ATCC 27853 *Ab – A. baumannii* ATCC 15308; SI – selectivity index;); MBIC - minimum biofilm inhibitory concentration, MBEC - minimum biofilm eradication concentration; color indicates the best SIs in series higher than 10, color indicates SIs in the 5 to 10 range, color indicates SIs in the 1 to 5 range.
